# Optimized Null Model for Protein Structure Networks

**DOI:** 10.1371/journal.pone.0005967

**Published:** 2009-06-26

**Authors:** Tijana Milenković, Ioannis Filippis, Michael Lappe, Nataša Pržulj

**Affiliations:** 1 Department of Computer Science, University of California Irvine, Irvine, California, United States of America; 2 Max Planck Institute for Molecular Genetics, Berlin, Germany; Center for Genomic Regulation, Spain

## Abstract

Much attention has recently been given to the statistical significance of topological features observed in biological networks. Here, we consider residue interaction graphs (RIGs) as network representations of protein structures with residues as nodes and inter-residue interactions as edges. Degree-preserving randomized models have been widely used for this purpose in biomolecular networks. However, such a single summary statistic of a network may not be detailed enough to capture the complex topological characteristics of protein structures and their network counterparts. Here, we investigate a variety of topological properties of RIGs to find a well fitting network null model for them. The RIGs are derived from a structurally diverse protein data set at various distance cut-offs and for different groups of interacting atoms. We compare the network structure of RIGs to several random graph models. We show that 3-dimensional geometric random graphs, that model spatial relationships between objects, provide the best fit to RIGs. We investigate the relationship between the strength of the fit and various protein structural features. We show that the fit depends on protein size, structural class, and thermostability, but not on quaternary structure. We apply our model to the identification of significantly over-represented structural building blocks, i.e., network motifs, in protein structure networks. As expected, choosing geometric graphs as a null model results in the most specific identification of motifs. Our geometric random graph model may facilitate further graph-based studies of protein conformation space and have important implications for protein structure comparison and prediction. The choice of a well-fitting null model is crucial for finding structural motifs that play an important role in protein folding, stability and function. To our knowledge, this is the first study that addresses the challenge of finding an optimized null model for RIGs, by comparing various RIG definitions against a series of network models.

## Introduction

### Background and motivation

Network-based analyses of protein structures have received much attention in recent years. In such a framework, protein structures are modeled as “residue interaction graphs” (RIGs) where nodes represent amino acid residues and edges describe pair-wise contacts between residues. A contact between two residues is defined if the distance between any pair of their heavy atoms is within a specified distance cut-off. Several studies have related network topological properties, such as network centrality, to protein folding and binding mechanisms, as well as to protein stability and function. For example, betweenness-centrality, the number of shortest paths that pass through a node, can be utilized to identify key residues that act as nucleation centers in protein folding [1], or that are involved in protein-protein interactions [2]. On the other hand, closeness-centrality, the average shortest path distance between a node and all the other nodes in the network, suggests critical residues for protein function [3], [4] and viable circular permutants [5]. Conserved residues that are responsible for maintaining a low average shortest path length have been shown to be important for allosteric communication [6], while conserved clusters of residues or highly connected residues have been associated to protein stability [7], [8]. Moreover, protein folding kinetics are topology-dependent. It has been shown that contact order, the average sequence separation over all contacting residues, as well as the number of non-local contact clusters in residue interaction graphs (RIGs) correlate well with folding rates [9], [10]. The probability of a given conformation to fold has also been linked to network topology [11]. Non-network-topology-based, but related approaches model the geometry of amino acid packings by a random packing of hard spheres from condensed matter physics [12], or the geometry of the packing around individual residues by the regular lattice model for sphere packing [13].

Modeling biological networks is of crucial importance for any computational study of these networks. Only a well-fitting network model that precisely reproduces the network structure and laws through which the network has emerged can enable us to understand and replicate the underlying biological processes. A good null model can be used to guide biological experiments in a time- and cost-optimal way and to predict the structure and behavior of a system. Since incorrect models lead to incorrect predictions, it is vital to have as accurate a model as possible.

Thus far, graph null models that take into account the network size and the overall degree distribution have been formulated in the field of protein-protein interaction networks [14], [15]. These random models were utilized as the reference state to identify interaction patterns that are over-represented in the experimentally observed networks [15] and to compare the behavior of certain topological properties [14]. It has been argued that such a realistic but simple approach for defining a null model might wrongly identify as significant the motifs that result from other topological features not taken into account by the null model [16].

Similarly, network analyses of protein structures have been mainly focusing on the degree distribution. It has been shown that the Poisson probability model best describes the degree distribution of RIGs [2], [17], [18]. However, when only long-range interactions are considered, an exponential distribution with a single-scale, fast decaying tail is observed. This distribution exhibits, to some extent, scale-free properties [17]. Characteristic path lengths and clustering coefficients of RIGs have been modeled by a variant of small-world networks [19]. However, a random rewiring of RIGs, that keeps the number of contacts of each residue fixed, affects the characteristic path length and clustering coefficient and thus such random networks lose the observed small-world character of RIGs [18]. Therefore, the choice of an appropriate network null model is of crucial importance when determining the statistical significance of network properties [18], [20]. Despite the fact that previous network analyses of RIGs have provided valuable insight, a null model that captures the network organization of protein structures has not been established. The only related work suggested that a coarser representation of protein structures, in which nodes correspond to secondary-structure elements, has the same network motifs as does a variant of geometric graphs [21].

### Our approach

Here, we address this important challenge of finding an appropriate null model for protein structure networks. Geometric random graphs model spatial relationships of objects: two objects that are close enough in space will interact, whereas two distant objects will not. For this reason, they are expected to mimic well the underlying nature of packed residues in a protein. Motivated by this premise, we assess the fit of this model to protein structure networks and demonstrate that it indeed fits RIGs better than any of the other analyzed network models. In addition to 3-dimensional geometric random graphs (“GEO-3D”) [22], we also used the following network models: Erdös-Rényi random graphs (“ER”) [23], random graphs with same degree distribution as the RIGs (“ER-DD”), Barabási-Albert type scale free networks (“SF-BA”) [24], and stickiness-index based networks (“STICKY”) [25] (see Methods).

Exact comparisons of large networks are computationally infeasible due to the underlying subgraph isomorphism problem, which is a problem of determining whether a graph contains a given subgraph, and which can not be solved in polynomial time [26]. Thus, to evaluate the fit of the data to the model networks, we compare the RIGs to the model networks with respect to easily computable *network properties*. We use GraphCrunch, our software tool for large network analyses and modeling [27], to model RIGs and to evaluate the fit of different models to the data. To overcome the limitations introduced by using a single network property (such as the degree distribution), we perform a fine-grained analysis of RIGs that is based on a emphmultitude of network properties (described in Methods). We use two highly-constraining graphlet-based measures of structural similarity between two networks, where *graphlets* are small substructures of large networks [28]: *relative graphlet frequency distance (RGF-distance)* [28] and *graphlet degree distribution agreement (GDD-agreement)* [29]. Additionally, we use five standard network properties: the *degree distribution*, the *clustering coefficient*, the *clustering spectrum*, the *average network diameter*, and the *spectrum of shortest path lengths*.

We perform systematic analysis on various RIG definitions. In addition to a series of distance cut-offs and the all-atom protein representation, we examine two more protein representations. First, we consider the sub-network that only takes *backbone* atoms into account and is dominated by short-range contacts reflecting secondary structure preferences. Second, we use the *side-chain-side-chain* interaction network that considers side-chain directionality and in which interacting residues are close in space but usually not close in sequence (see Section “Data Sets”).

Overall, the main question we address here is determining which random graph model keeps the observed topological characteristics of RIGs. We demonstrate that geometric random graphs provide an excellent fit to RIGs of all fold-types and contact definitions considered in this study. Also, we examine how protein size, structural class, protein thermostability, and quaternary structure affect the strength of the fit. We show that geometric random graphs capture the network organization of RIGs better for larger than for smaller proteins. Moreover, for proteins of the same size, the fit is better for proteins with low helical content. Furthermore, the tighter packing of the solvent accessible surface in thermostable proteins [30] leads to a worse fit. Additionally, we conclude that the quaternary association of proteins has no impact on the fit of geometric random graphs. Finally, to illustrate the importance of using an appropriate network null model, we perform network motif search in RIGs with respect to different random graph models. We show that it is important to choose GEO-3D model, to identify as specifically as possible subgraphs that are statistically significantly over- and under-represented. This might lead to unraveling of the important features of the protein structural space.

## Results and Discussion

### Data Sets

First, we analyze single chain RIGs for nine structurally diverse proteins: 1agd, 1fap, 1ho4, 1i1b, 1mjc, 1rbp, 1sha, 2acy and 3eca. We construct multiple RIGs as undirected, unweighted graphs for each of these proteins as follows. Two residues in a protein are considered to interact if any heavy atom of one residue is within the specified distance cut-off of any heavy atom of the other residue. We set distance cut-offs to range from 4.0 to 9.0 Å (Section S1.1). We examine various representations of residues, hereafter referred to as *contact types*. We denote by “BB” (“SC”) the RIGs that contain as edges only the residue pairs that have heavy backbone (side-chain) atoms within the given distance cut-off. We denote by “ALL” the most commonly used RIG representation, in which all heavy atoms of every residue are taken into account when determining residue interactions. Thus, in this data set, we analyze 513 RIGs for the nine proteins constructed for 19 distance cut-offs and the three contact types of “BB”, “SC”, and “ALL.” Henceforth, we refer to this data set as *Data Set 1*.

Next, to ensure that our results are applicable to a wide range of proteins, we analyze an additional data set of 1,272 RIGs corresponding to a non-redundant data set of 1,272 proteins, pre-compiled by the PISCES server [31]. These RIGs are constructed with the most commonly used “ALL” contact type and distance cut-off of 5 Å. Henceforth, we refer to this data set as *Data Set 2*. Proteins in this data set are of different size and they belong to four different protein structural classes according to the Structural Classification of Proteins (SCOP) [32] (Section S1.1): 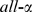 *proteins* (class “A”) consisting entirely of 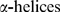, 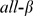 *proteins* (class “B”) consisting entirely of 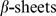, 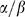 *proteins* (class “C”) consisting of alternating 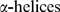 and 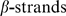 along the backbone with 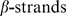 therefore being mostly *parallel*, and 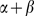 *proteins* (class “D”) consisting of 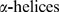 and 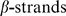 that occur separately along the backbone with 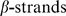 therefore being mostly *antiparallel*. Also, proteins in this data set have different quaternary structure and are of different oligomerization order, as predicted by the Protein Interfaces, Surfaces and Assemblies (PISA) server [33].

Finally, to examine the effect of RIG structure of thermostable proteins to the fit of GEO-3D, we analyze a high quality data set of 94 pairs of proteins where in each pair, one protein comes from *T. maritima*, a representative of thermophiles, and the other is its close homolog from a mesophilic species [34]. Thermophilic proteins are on average shorter and have higher average connectivities and clustering coefficients compared to mesophilic ones [34]. For these proteins, we construct RIGs using “ALL” contact type and distance cut-off of 4.5 Å; the same criteria was used by Robinson-Rechavi et al. [34]. We refer to this data set as *Data Set 3*. Details about the analyzed data sets can be found in Section S1.1.

In total, we analyze 1,973 RIGs corresponding to 1,469 proteins. Below, we first evaluate the fit of GEO-3D to these RIGs and then we analyze effects of protein size, class, thermostability, and quaternary structure to the fit of GEO.

### Topological Analysis

We find that all network properties offer support to the superiority of the GEO-3D network model to a large number of RIGs that correspond to a wide range of structurally diverse proteins, constructed using various contact types and a wide range of distance cut-offs. Given that RIGs considering only backbone or side-chain atoms as interacting sites are quite different from one another with respect to the set of interacting residues, the robustness of our result across various RIG definitions is quite surprising.

**Figure 1:**
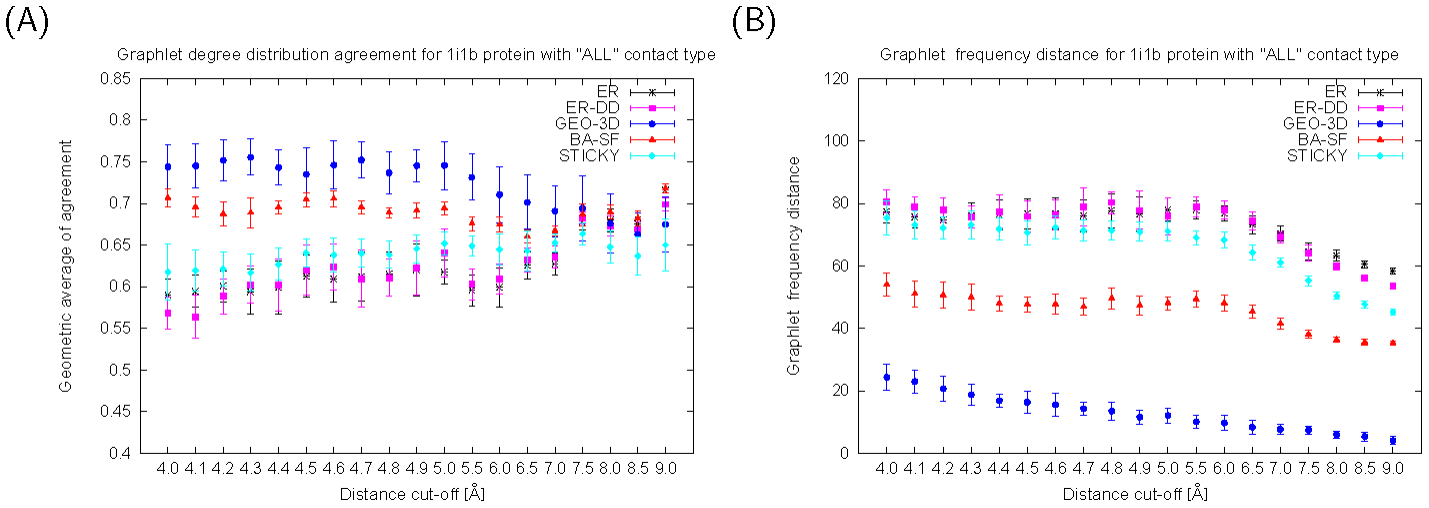
The fit of network models to RIGs corresponding to 1i1b protein. (A) GDD-agreements and (B) RGF-distances between model networks (ER, ER-DD, GEO-3D, SF-BA, and STICKY) and RIGs corresponding to 1i1b protein, that are constructed for “ALL” contact type and a series of distance cut-offs between 4.0 and 9.0 Å. The larger the GDD-agreement in panel A, the better the fit. The smaller the RGF-distance in panel B, the better the fit.

For all of the RIGs in Data Set 1, RGF-distances and GDD-agreements between the RIGs and the model networks strongly favor geometric random graphs (Figure 1 and Figure S1.1 to Figure S1.9). Based on RGF-distances (with minimal exceptions described below), the fit of the GEO-3D model is the best for all nine proteins, all three contact types and all of the distance cut-offs; the exceptions are the lowest distance cut-offs ([4.0–4.2]) Å for “SC” contact type for four out of nine proteins only. GDD-agreement favors GEO-3D model for all proteins, all three contact types, and all of the distance cut-offs lower than about 6.5 Å. When larger distance cut-offs are used to construct RIGs, RIGs become well-fitted by Erdös-Rényi (ER) random graphs, while it is widely believed that ER random graphs are not a good model for any real-world networks. We regard an overlap of the fit of GEO-3D with ER random graphs in all nine proteins to be the discriminative factor when suggesting the distance cut-off threshold of 6.5 Å, since we observed it for most of the proteins that we analyzed (Figure 1 A and Figure S1.1 to Figure S1.9 A–C). Since choosing an optimal distance cut-off is an important problem in network-based analyses of protein structures, our results imply that distance cut-offs lower than 6.5 Å might need to be used for RIG construction. Note however that a data set larger than nine proteins might need to be analyzed before such a generalization could be made.

The magnitude of GDD-agreement between RIGs and GEO-3D graphs seems to be related to protein size. The two smallest proteins, 1mjc and 1fap, have GDD-agreements of up to around 0.7, while the largest protein, 3eca, has much higher GDD-agreements of up to 0.85. Following this observation, we analyze below the effect of protein size on the strength of the fit of GEO-3D to RIGs in more detail. Moreover, the RGF-distances between the RIGs and the geometric random graphs are usually higher (meaning worse fit) for “SC” networks compared to networks of other contact types. Since side-chains are more mobile compared to the rigid backbone [35], we expect that “SC” networks form more complex interaction patterns compared to networks that contain backbone interactions. There is also a general trend that RGF-distance decreases with increasing distance cut-off, independent of the network model. Equivalently, GDD-agreement increases as the distance cut-off increases for most of the models. Since both the smaller RGF-distance and the larger GDD-agreement indicate improved fit of the network model to RIG, these observations might suggest that for higher distance cut-offs, graphlets of higher order are needed to improve the quality of the fit to the data.

We also examine the fit of the network models to the RIGs with respect to five standard network properties. Illustrations showing Pearson's correlation coefficients between the degree distributions of the 513 RIGs constructed for the nine proteins and the corresponding model networks are presented in Figure S1.10 to Figure S1.18. ER and GEO-3D models both have Poisson degree distributions and thus are tied in that they both reproduce well the degree distributions of all of the 513 RIGs. ER-DD networks have exactly the same degree distribution as the data by construction and thus they reproduce this property perfectly. Similarly, STICKY model networks are constrained to have the expected degree distribution of real networks (see [25] for details). Only BA-SF networks have power-law degree distributions by construction and thus are expected to have worse fit to RIGs with respect to this property. Indeed, all of these are observed for all of the 513 RIGs that we analyzed (Figure S1.10 to Figure S1.18).

Also, GEO-3D model networks reproduce well the clustering spectra of the RIGs for distance cut-offs smaller than 8 Å (Figure S1.10 to Figure S1.18). Similarly, the average clustering coefficients of almost all of the 513 RIGs are generally best reproduced by GEO-3D networks (Figure S1.19 to Figure S1.27). There exist very few exceptions to this observation. For a very small number of distance cut-offs lower than 5.0 Å in the “SC” RIGs of five proteins, the clustering coefficients of BA-SF networks describe the best those of the corresponding RIGs. Interestingly, all small proteins with size less than 105 residues (1agd, 1fap, 1mjc, 1sha, 2acy) are included in the set of these five proteins. Also, we notice the trend that for all proteins and all contact types, the higher the cut-off, the better the fit of clustering coefficient between the GEO-3D model and the data. The average diameters of all RIGs are best reproduced by the GEO-3D networks for all distance cut-offs of “BB” and “ALL” contact types (Figure S1.19 to Figure S1.27). The same is true for almost all of the RIGs of “SC” contact type; only for the lowest distance cut-offs of several proteins, ER and ER-DD models provide a better fit. Note also that for these “SC” RIGs of low distance cut-offs, the diameters of the RIGs are close to being within one standard deviation of the average diameters of GEO-3D networks. Finally, GEO-3D model provides the best fit to RIGs with respect to shortest path length spectra. This is true for all nine proteins, all three contact types, and all 19 distance cut-offs with the exception of the lowest distance cut-offs for “SC” contact type (Figure S1.28 to Figure S1.36).

**Figure 2:**
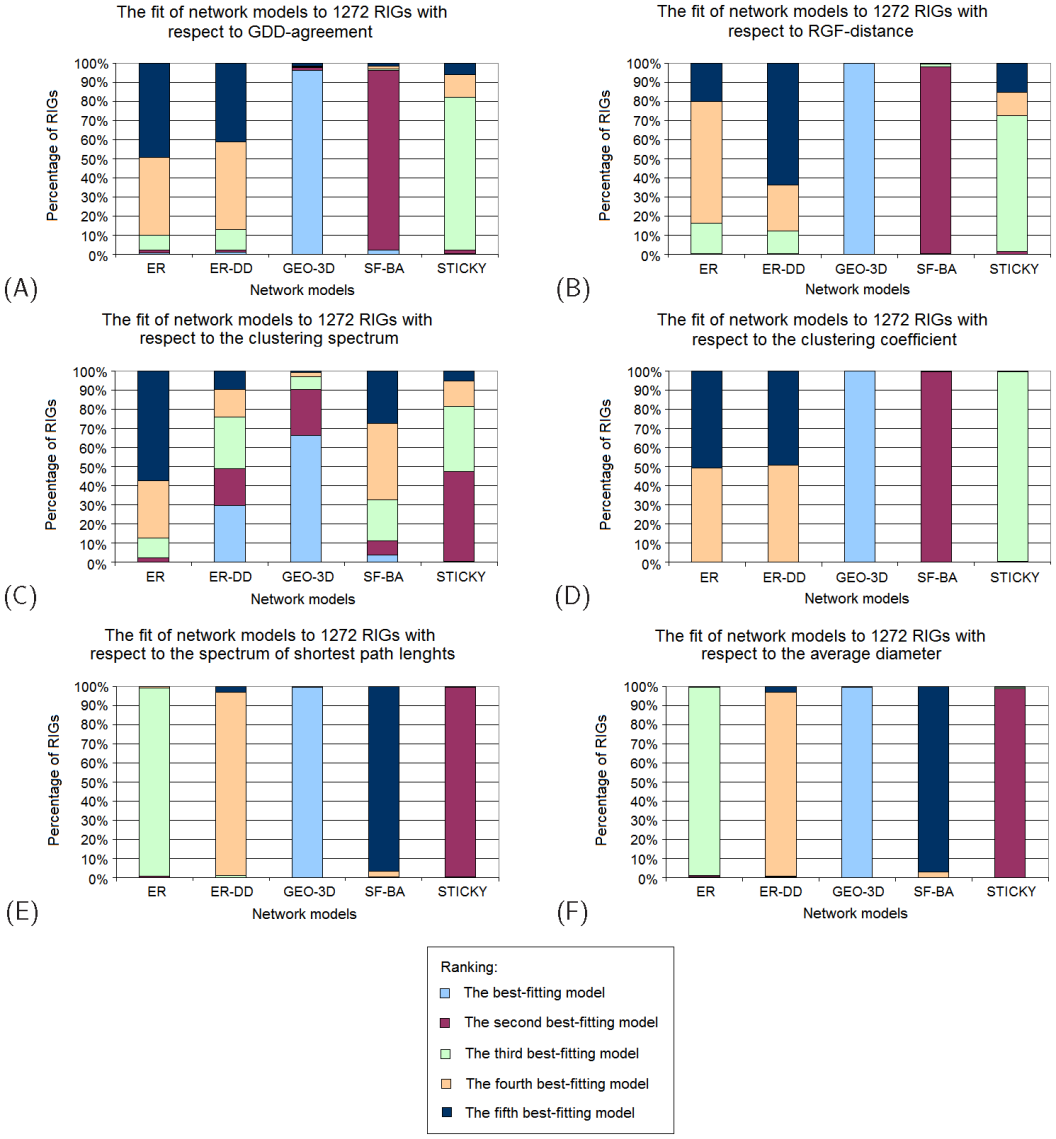
The fit of network models to RIGs in Data Set 2. The ranking of five network models (ER, ER-DD, GEO-3D, SF-BA, and STICKY) for 1,272 RIGs corresponding to the 1,272 proteins, constructed with “ALL” contact type and distance cut-off of 5 Å. The ranking is based on: (A) GDD-agreements between RIGs and model networks, (B) RGF-distances between RIGs and model networks, (C) agreements between clustering spectra of RIGs and model networks, (D) agreements between clustering coefficients of RIGs and model networks, (E) agreements between spectra of shortest path lengths of RIGs and model networks, and (F) agreements between average diameters of RIGs and model networks.

To examine the fit of model networks to RIGs corresponding to a larger number of proteins, we analyze Data Set 2 (described in Section “Data Sets”). We summarize the results of the fit of each of the five network models to these 1,272 RIGs with respect to each of the above described network properties, by measuring the percentage of RIGs for which a given network model is the best-fitting null model for a given property, the percentage of RIGs for which a given network model is the second best-fitting null model for a given property, etc. (Figure 2). GEO-3D is the best-fitting null model for almost all RIGs with respect to all network properties except for the degree distribution where the fit is as described for Data Set 1 above.

Similar results are obtained for all RIGs in Data set 3: GEO-3D is the best-fitting null model for almost all RIGs corresponding to both mesophilic (Figure S1.37) and thermophilic proteins (Figure S1.38). This is true for all network properties, with the exception of the degree distribution, which behaves as explained above.

From the above analyses, we conclude that GEO-3D graphs are the best-fitting null model for various graph representations of protein structures. This result is encouraging, since GEO-3D graphs model spatial relationships of objects, and therefore, they are expected to mimic well the underlying nature of packed residues in proteins. Furthermore, our result concurs with previous studies focusing on degree distributions and small-world characters of protein structure networks [2], [17], [18].

### The quality of the fit of geometric random graph model

We first analyze whether the strength of the fit of GEO-3D to RIGs changes with RIG size. Here, we consider all 1,272 RIGs from Data Set 2. Our data points are network property values describing the agreement of RIGs of a given size and the GEO-3D model. We find that the fit of GEO-3D is strongly correlated with RIG size and that this correlation can be expressed as a power-law function. We find such function that fits the data in the least-squares sense, for each of the network properties. By quantifying the goodness of fit of such functions to the observed correlation data using R-Square measure, we observe that their fit is good for most network properties (Section S1.2.1 and Figure S1.39 to Figure S1.41).

Specifically, as protein size increases, the fit also noticeably increases with respect to GDD-agreement and degree-distribution. This trend is also observed, in a somewhat less pronounced way, with respect to RGF-distance. Surface residues are less well packed compared to buried residues, leading to a heterogeneous density distribution. However, for larger proteins, the percentage of buried residues, as well as the packing density of the solvent-exposed residues increase [36]. Therefore, for larger proteins, the degree distribution and the interaction patterns of the residues become more homogeneous, and thus, the network topology is better reproduced by the geometric random graphs. The fit of GEO-3D improves rapidly up to approximately 200 residues and then it slowly converges (Figure S1.39). This behavior has also been observed in the average protein packing as a function of the size and has been attributed to the size distribution of mono-domain proteins [36].

Average diameters of both RIGs and GEO-3D graphs increase with protein size, while clustering coefficients slightly decrease (Figure S1.39 E and F). Thus, the fit of GEO-3D to RIGs with respect to these properties is independent of protein size. Similarly, the fit of GEO-3D shows no correlation with protein size with respect to clustering spectrum and spectrum of shortest path lengths (Figure S1.39 D and G).

Second, we examine whether the strength of the fit of GEO-3D depends on the protein secondary structure. We analyze RIGs in Data Set 2 that belong to four structural classes described in Section “Data Sets.” We show that within each of the structural classes, there exists a strong correlation between the fit of GEO-3D and protein size with respect to most network properties (Figure S1.40 and Figure S1.41). We evaluate the statistical significance of the difference of the fit of GEO-3D across structural classes by performing ANOVA statistical test; low *p*-values indicate that the fit of GEO-3D to proteins of a given size belonging to the classes being compared is significantly different (Section S1.2.2). Since GDD-agreement is not only the most constraining network property, but also encompasses all other network properties [29], we perform this analysis with respect to GDD-agreement only. The difference in the fit is statistically significant over all class pairs (p-values<0.077) apart from A–C and B–D pairs (Section S1.2.2 and Figure S1.42 A). In class C proteins, the percentage of residues that are in 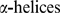 is higher than the percentage of residues that are in 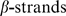 compared to class D proteins (Figure S1.43 A). That is, classes A and C have higher helical content than classes B and D, and therefore, they are structurally more similar to one another than to the remaining two classes. This further validates the correctness of our GEO-3D model, since it successfully distinguishes between structurally different classes.

Moreover, for larger proteins with more than 300–350 residues, the fit of GEO-3D is the highest for class B proteins, followed by class D, class C, and class A proteins (Figure S1.42 B). This implies that the fit of GEO-3D decreases with a decrease in 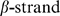 content in a protein. On the other hand, for smaller proteins with less than 300–350 residues, the fit of GEO-3D to class D proteins is higher than to class B proteins, even though they have lower 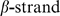 content compared to class B proteins. Nonetheless, this could be attributed to the higher percentage of non-regular secondary structural elements, being neither helix nor strand (e.g., loop) in class B proteins of small size (Figure S1.43 B). Additionally, we verify that class C proteins are more compact compared to proteins of equal size from other structural classes (Figure S1.44 B and C), confirming a previous result by Galzitskaya et al. [37].

Third, after verifying that GEO-3D is the best-fitting model for both mesophilic and thermophilic RIGs from Data Set 3 (Section “Topological Analysis”), we evaluate the effect of the structural features responsible for protein thermostability on the strength of the fit of GEO-3D. It has been shown that thermophilic proteins are on average shorter and have higher average connectivity and clustering coefficient compared to mesophilic ones [34]. We confirm this although we use different RIG definition (Table S1). Moreover, the increase in packing density is observed for highly connected residues [34] and for solvent-exposed ones [30]. We observe the difference in the fit of GEO-3D graphs to thermophilic and the corresponding mesophilic proteins (Figure S1.45). We examine the statistical significance of the difference using Student's pairwise *t*-test (Section S1.2.3). We demonstrate that the fit is significantly higher for mesophilic than for thermophilic proteins with respect to GDD-agreement, degree distribution, and clustering coefficient (Table S1). Thus, the tighter packing of the solvent accessible surface in thermostable proteins seems to lead to a worse fit. Additionally, consistent to our results described above, it is possible that higher fit of GEO-3D to mesophilic proteins is due to their larger size.

Similarly, we examine the effect of the quaternary structure to the fit of GEO-3D to RIGs. The network topology on the surface of a protein is expected to differ between monomers and multimers. Protein-protein interfaces tend to be more hydrophobic than the non-interface surface, while interface residues are more well packed [38]. We analyze 75 pairs of monomeric and multimeric proteins from Data Set 2. Proteins in each pair have equal size and belong to the same structural class, eliminating any bias due to these structural features. Although we show that monomers have significantly higher number of contacts per residue and lower average diameter compared to multimers, we observe no significant difference in the fit of GEO-3D between monomers and multimers, with respect of any of the network properties (Section S1.2.4 and Table S2).

### Application to Motif Detection

**Figure 3:**
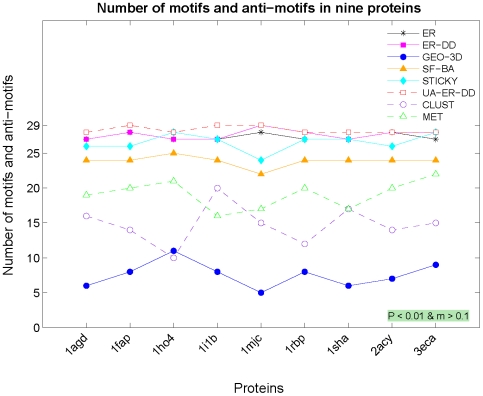
Motif counts for RIGs in Data Set 1 using different network null models. The total number of motifs and anti-motifs identified in nine RIGs corresponding to the nine proteins (1adg, 1fap, 1ho4, 1i1b, 1mjc, 1rbp, 1sha, 2acy, and 3eca), constructed with “ALL” contact type and 5.0 Å distance cut-off. The motifs and anti-motifs were identified with respect to the eight network models (ER, ER-DD, GEO-3D, BA-SF, STICKY, UA-ER-DD, CLUST, and MET). The threshold values used for motif selection are displayed within the colored textbox (*P*-value lower than 0.01 and *M*-factor greater than 0.1).

To illustrate the importance of the choice of the appropriate null model to a network-based analysis of protein structures, we examine the issue of identifying network motifs in RIGs. Since motifs (anti-motifs) are over-represented (under-represented) subgraphs that appear in a real-world network at frequencies that are much higher (lower) than those of their corresponding random graph models [15], motif discovery requires comparing real-world networks with model networks. Using an inadequate model may identify as over-represented (under-represented) subgraphs that otherwise would not have been identified as motifs (anti-motifs). For example, all non-geometric models that we analyzed, which preserve only some topological properties of RIGs, tend to identify as significantly (under-) over-represented *almost all* analyzed subgraphs (Figure 3). Therefore, it is questionable whether these non-discriminative network models could be used to accurately assess the statistical significance of subgraphs in RIGs that are relevant with respect to network structure. We show that among all analyzed models, GEO-3D model exhibits the highest “specificity” in the selection of network motifs: only 5–11 out of 29 subgraphs, depending on a protein, are identified as (anti-)motifs when GEO-3D graphs are used as the null model (Figure 3 and Figure S1.47). Since we have shown above that GEO-3D networks provide the best fit to RIGs, this is an additional validation that GEO-3D is an optimal null model for RIGs.

More specificaly, we use mfinder [39] to search for all undirected subgraphs on 3, 4, and 5 nodes (Figure S1.46) in RIGs corresponding to the nine proteins in Data Set 1. In addition to our five network models, we use the three standard models supported by mfinder (Section S1.3). In Figure S1.48 to Figure S1.56, we present the absolute Z-scores and M-factors (described in Section S1.3), i.e., the motif selection criteria, for all 3- to 5-node subgraphs in each of the nine RIGs, with respect to each of the eight network models. We do not attempt to relate the identified network motifs with protein 3-dimensional structure or function; the purpose of our analysis is to demonstrate that non-geometric null models, that do not fit well the RIG network structure, might wrongly identify numerous subgraphs as important with respect to network structure. Furthermore, we demonstrate that the results obtained by using a geometric network model can not be reproduced with other network models by simple adjustment of the motif selection criteria (Section S1.3).

### Comparison with Other Studies

Raghunathan and Jernigan focused on examining the packing of residues around each individual residue, by analyzing the distribution of the number and the regularities in the directions of sequentially non-adjacent residues around a central residue [13]. They found that the geometry of this packing around a residue conforms almost perfectly to the regular lattice model of dense packing of uniform spheres. Their conclusion holds for a single distance cut-off (6.5 Å ) and for “non-bonded,” i.e., sequentially non-adjacent, residue interactions only. The authors provide a limited evaluation, on four proteins only, of the fit of the “layered lattice” obtained by forming an array of repeated residue-centered lattice points in space to the overall protein structures. In contrast, we examine the fit of GEO-3D to 1,973 RIGs constructed for a set of 1,469 structurally different proteins using a series of distance cut-offs and RIG definitions. Furthermore, their study differs from ours in the following: when evaluating the fit of residue packing to the layered lattice, the authors simply compute RMS deviation between the corresponding pairs of actual residue positions in a protein and points in the lattice. Thus, they compute the distances between the corresponding points in the real-world 3-dimensional space. They do not compare the *structure* of the two networks (the RIG and the lattice) from the aspect of graph-theory. For our GEO-3D model and for RIGs, we do not care about the exact spatial positions of the nodes, but about their graph structure. Thus, GEO-3D graphs could be viewed as existing in an “abstract” (or “latent”) space and the comparison of the topologies of GEO-3D and RIG networks is based on network structural properties rather than on spatial distances between nodes.

Soyer et al. analyze the packing of residues in proteins by modeling protein structures with Voronoï tessellation (VT), i.e., by dividing the real-world 3-dimensional space occupied by a protein structure into a set of VT cells, one cell per residue [12]. The mean distances between amino acids in their study are in the 6.8–7.5 Å range. They observe that two of the characteristics of the VT cells, averaged over all 40 proteins that they analyze, are similar to those of random packing of hard spheres in condensed matter physics. The good fit of *random* hard sphere packing to protein structures could be explained by our finding that residue distances above 6.5 Å yield RIGs whose structure is consistent with the structure of Erdös-Rényi *random* graphs (see “Topological Analysis” section above). Similar to Raghunathan and Jernigan [13], Soyer et al. do not perform graph-theory-based network structural comparisons, which makes it hard to directly compare their study with ours.

Finally, Bartoli et al. explicitly use network-based approaches to examine RIGs [19]. However, they do not model RIGs in the same way we do: whereas we generate GEO-3D random networks using a generation process that does not require any knowledge about a real-world RIG other than its size, Bartoli et al. simply create randomized contact maps (i.e., RIGs). Thus, their “necklace” model is not a network model, but a RIG randomization strategy: in the “necklace” model, all residues on the backbone of a RIG are connected as on the thread of a necklace, and also non-backbone-adjacent residues interact with a probability proportional to their proximity on the thread (i.e., on the protein sequence). Thus, the necklace model is not 3-dimensional, since it is based on sequence distances between residues rather than on their spatial distances. Therefore, this model could be considered as a distorted version of 1-dimensional geometric random graph, or as a variant of the Watts-Strogatz small-world network [40]. Since proteins exist in 3-dimensional space, it is not to be expected that a 1-dimensional network model would provide a better fit to them than a 3-dimensional network model. The reported wellness of fit of the “necklace” model is likely due to the examination of only the clustering coefficients and characteristic path lengths averaged over all analyzed proteins. In contrast, we examine a much more comprehensive set of network properties and do not average them over all RIGs.

### Future Directions

Our geometric random graph null model may facilitate further graph-based studies of protein conformation. This analysis may also have important implications for protein structure comparison and prediction. For example, Contact Map Overlap (CMO) problem [41] measures protein structural similarity based on a graph alignment of contact maps; a contact map is simply the adjacency matrix of a RIG, while contact map similarity is the maximum number of common contacts that can be efficiently approximately computed. A correct random graph model could provide means of assessing the statistical significance of contact map similarity. Expectation scores (E-values) express the probability that the observed similarity could have arisen by chance. E-values for the traditional structural alignment are derived by using the background information, i.e., the distribution of alignment scores for random proteins that are structurally dissimilar to the query protein. Instead, given our null model, expectation scores can be defined based on the alignment of the query contact map against a random contact map generated according to our model.

Additionally, specific topologies of secondary structure elements or their parts have already been shown to constitute known structural or functional motifs, such as the helix-turn-helix motif found in DNA-binding proteins [42], or the catalytic triad of the serine proteases [43]. Our results may further facilitate the discovery of such important motifs from the network structure of RIGs, even in the absence of homologs. Instead of comparing the protein of interest against existing structures, it might be sufficient to compare the observed RIG against the randomized counterparts.

Finally, it would be interesting to investigate to which extent our analysis could contribute to reliable discriminatory functions that can distinguish near-native conformations from non-native ones. Graph properties of RIGs have been already utilized in this direction [44]. Similarly, the strength of the fit of geometric random graphs to the RIG of a predicted conformation might indicate how native-like the specific protein conformation is.

The null model proposed here is only topologically similar to protein structure networks. A possible area for improvement is to refine it based on additional biophysical properties. According to the model, nodes correspond to points in space distributed uniformly at random and without any preference. In reality, two residues prefer to be connected based on their sequence separation, their residue type, their secondary structure, or even their neighborhood. Moreover, the chain connectivity imposes constraints that are currently neglected. Thus, further refinements of the geometric model, that would incorporate these biological properties, are expected to yield an even better fitting null model for protein structure networks.

## Methods

### Network Models

For each RIG, we evaluate the fit of five different random graph models. The network models are implemented as follows. Erdös-Rényi random graphs (“ER”) are generated by using the LEDA [45] implementation of 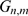, a random graph 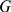 with 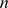 nodes and 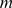 edges. These networks typically have small diameters, Poisson degree distributions, and low clustering coefficients. Random graphs with the same degree distribution as the data (“ER-DD”) are generated by using the “stubs method” (see section IV.B.1 of [46] for details). This model captures the degree distribution of a real-world network while leaving all other aspects as in ER model. Scale-free (“SF-BA”) networks are generated by using the Barabási-Albert preferential attachment model [24], in which newly added nodes preferentially attach to existing nodes with probabilities proportional to their degrees; this model results in networks with power-law degree distributions. Geometric random graphs (“GEO”) are defined as follows: nodes correspond to uniformly randomly distributed points in a metric space and edges are created between pairs of nodes if the corresponding points are close enough in the metric space according to some distance norm. A variant of geometric random graphs in this study (“GEO-3D”) uses 3-dimensional Euclidean boxes and the Euclidean distance norm. Finally, “stickiness network model” (“STICKY”) is based on stickiness indices, numbers that summarize node connectivities: the probability that there is an edge between two nodes in STICKY model networks is directly proportional to the stickiness indices of the nodes, i.e., to the degrees of their corresponding residues in real-world RIGs (see [25] for details). Networks produced by this model have expected degree distributions of real-world networks.

Model networks were generated and compared to RIGs using GraphCrunch [27]. For all random graph models, parameters are chosen in such way that each of the generated model networks that corresponds to a RIG has the same number of nodes and the number of edges within 1% of those in the RIG. We generated 30 networks per random graph model for each of the 1785 RIGs. Thus, in addition to analyzing 1785 RIGs, we also analyzed 5×30×1785 = 267,750 model networks corresponding to the RIGs and compared them to the RIGs (see Section “Network Comparisons”).

### Network Comparisons

RIGs are compared to the corresponding model networks with respect to two graphlet-based *local* and five standard *global* network properties.

#### Local Network Properties

We used the following two measures of local structural similarities between two networks: relative graphlet frequency distance (*RGF-distance*) [28] and graphlet degree distribution agreement (*GDD-agreement*) [29] (defined below). They are based on *graphlets*, small connected non-isomorphic induced subgraphs of large networks [28]. Graphlets differ from network motifs [15] since they must be *induced* subgraphs, whereas motifs are *partial* subgraphs. An induced subgraph must contain all edges between its nodes that are present in the large network, while a partial subgraph may contain only some of these edges. Moreover, graphlets do not need to be over-represented in the data when compared with “randomized” networks while motifs do. Since the number of graphlets on 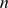 nodes increases exponentially with 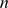, the RGF-distance and GDD-agreement computations are based on 2- to 5-node graphlets (see Figures 1 in [28] and [29], respectively).

*RGF-distance* compares the frequencies of the appearance of all 2- to 5-node graphlets in two networks. *GDD-agreement* generalizes the notion of the degree distribution to the spectrum of *graphlet degree distributions (GDDs)*. The degree distribution measures the number of nodes of degree 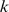, i.e., the number of nodes “touching” 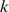 edges, for each value of 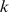, where an *edge* is the only graphlet with two nodes. GDDs generalize the degree distribution to other graphlets: they measure for each graphlet on 2 to 5 nodes, the number of nodes “touching” 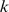 graphlets *at a particular node*. The node at which a graphlet is “touched” is relevant, because it is topologically important to distinguish between nodes “touching”, for example, a linear path on three nodes at an end-node or at the middle node. Thus, the “symmetries” between nodes of a graphlet need to be taken into account. This is summarized by 73 *automorphism orbits* for 2- to 5-node graphlets (see [29] for details). For each of the 73 orbits 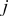, we measure the 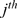 *GDD*, i.e., the distribution of the number of nodes “touching” the corresponding graphlet at orbit 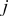 (thus, the degree distribution is the 1*^st^* GDD). We compare the 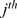 GDDs of two networks for each 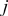 and combine the values of the comparisons into the GDD-agreement of two networks (see [29] for details); GDD-agreement is scaled to be between 0 and 1, where 1 means that the two networks are identical with respect to GDD-agreement. Since GDD-agreement encompasses the fit of each of the 73 GDDs of the networks being compared, it is a very strong measure of structural similarity between two networks.

#### Global Network Properties

We used the following global network properties: the degree distribution, the average clustering coefficient, the clustering spectrum, the average network diameter, and the spectrum of shortest path lengths. They are defined as follows. The *degree* of a node is the number of edges incident to the node. The *degree distribution*, *P(k)*, describes the probability that a node has degree 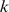. The *clustering coefficient* of node 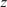 in a network, 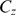, is the probability that two nodes 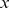 and 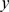 connected to the node 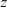 are themselves connected. The average of 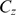 over all nodes 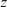 of a network is the *clustering coefficient*, *C*, of the network. The distribution of the average clustering coefficients of degree 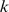 nodes is the *clustering spectrum*, *C(k)*. The smallest number of links that have to be traversed to get from node 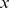 to node 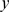 in a network is called the *distance* between nodes 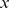 and 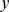 and a path through the network that achieves this distance is called the *shortest path* between 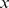 and 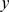. The average of shortest path lengths over all pairs of nodes in a network is called the *average network diameter*. The *spectrum of shortest path lengths* is the distribution of shortest path lengths between all pairs of nodes in a network.

## Supporting Information

Figure S1 Supplementary Figures
(3.29 MB PDF)

Section S1 Supplementary Text
(0.10 MB PDF)

Table S1 (0.02 MB PDF)

Table S2 (0.02 MB PDF)
