# Supplementary material for: Optimized Null Model for Protein Structure Networks
Source: PLoS One. 2009 Jun 26;4(6):e5967. doi: 10.1371/journal.pone.0005967 (PMC2699654; doi:10.1371/journal.pone.0005967)
Supplement: Figure S1 — Supplementary Figures (3.29 MB PDF) [file pone.0005967.s001.pdf]

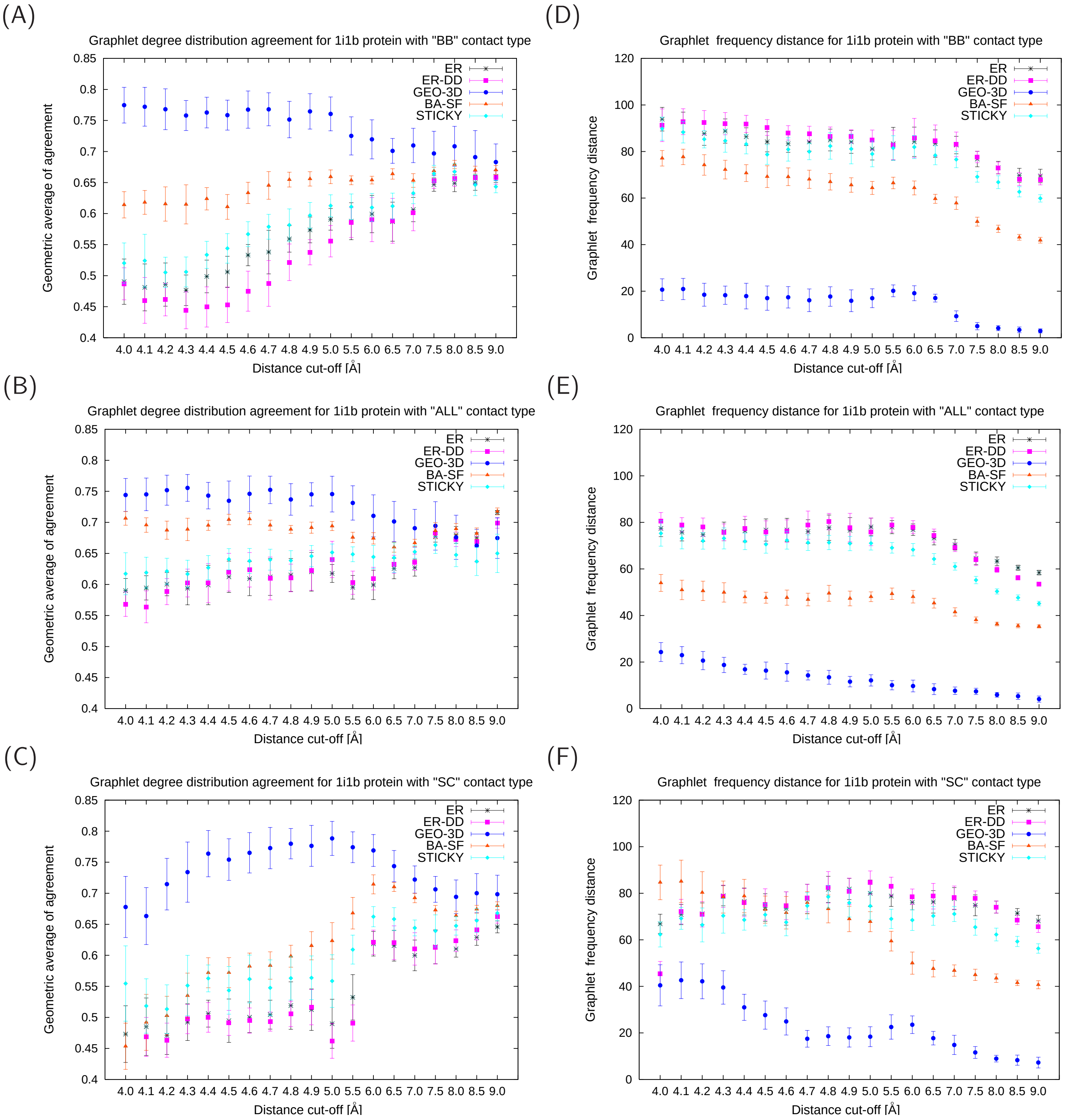

Figure S1.1 GDD-agreements and RGF-distances between model networks (ER, ER-DD, GEO-3D, SF-BA, and STICKY) and RIGs corresponding to 1i1b protein that are constructed for each of the three contact types ("BB", "ALL", and "SC") and a series of distance cut-off values between 4.0 and 9.0 Angstroms: **A.** GDD-agreement for "BB" contact type. **B.** GDD-agreement for "ALL" contact type. **C.** GDD-agreement for "SC" contact type. **D.** RGF-distance for "BB" contact type. **E.** RGF-distance for "ALL" contact type. **F.** RGF-distance for "SC" contact type. The larger the GDD-agreement in panels A-C, the better the fit. The smaller the RGF-distance in panels D-F, the better the fit.

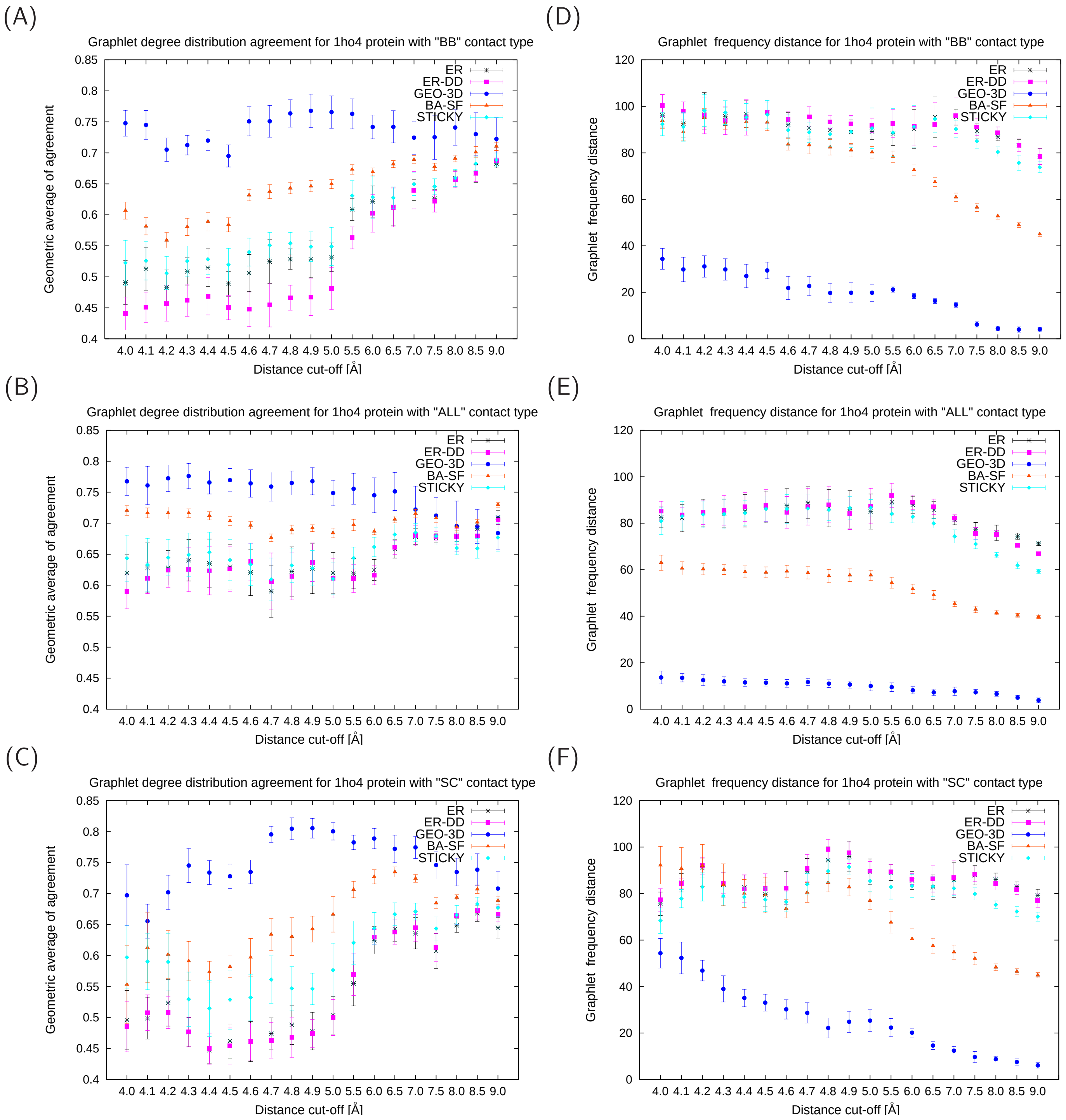

Figure S1.2 GDD-agreements and RGF-distances between model networks (ER, ER-DD, GEO-3D, SF-BA, and STICKY) and RIGs corresponding to 1ho4 protein that are constructed for each of the three contact types ("BB", "ALL", and "SC") and a series of distance cut-off values between 4.0 and 9.0 Angstroms: **A.** GDD-agreement for "BB" contact type. **B.** GDD-agreement for "ALL" contact type. **C.** GDD-agreement for "SC" contact type. **D.** RGF-distance for "BB" contact type. **E.** RGF-distance for "ALL" contact type. **F.** RGF-distance for "SC" contact type. The larger the GDD-agreement in panels A-C, the better the fit. The smaller the RGF-distance in panels D-F, the better the fit.

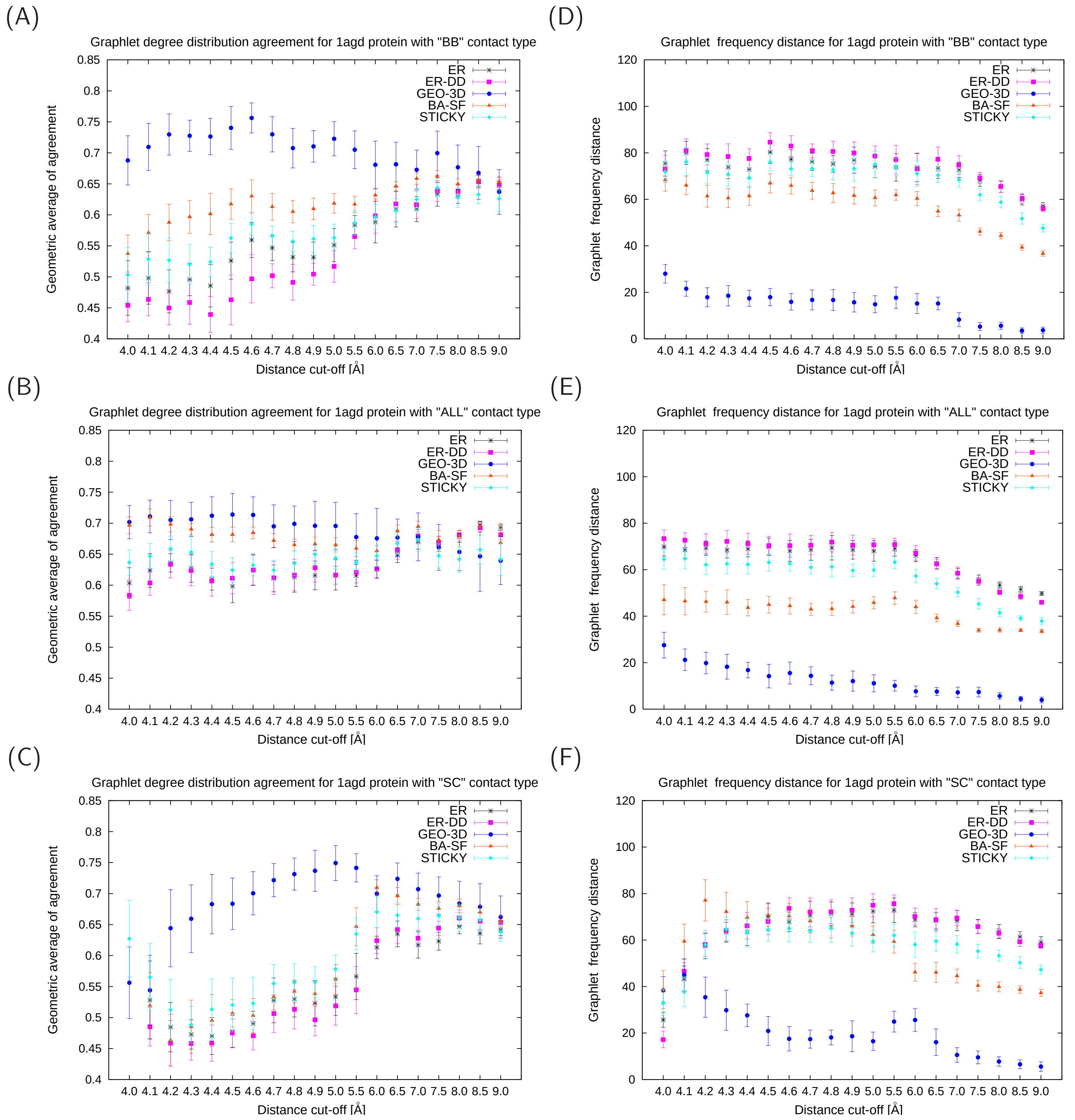

Figure S1.3 GDD-agreements and RGF-distances between model networks (ER, ER-DD, GEO-3D, SF-BA, and STICKY) and RIGs corresponding to 1agd protein that are constructed for each of the three contact types ("BB", "ALL", and "SC") and a series of distance cut-off values between 4.0 and 9.0 Angstroms: **A.** GDD-agreement for "BB" contact type. **B.** GDD-agreement for "ALL" contact type. **C.** GDD-agreement for "SC" contact type. **D.** RGF-distance for "BB" contact type. **E.** RGF-distance for "ALL" contact type. **F.** RGF-distance for "SC" contact type. The larger the GDD-agreement in panels A-C, the better the fit. The smaller the RGF-distance in panels D-F, the better the fit.

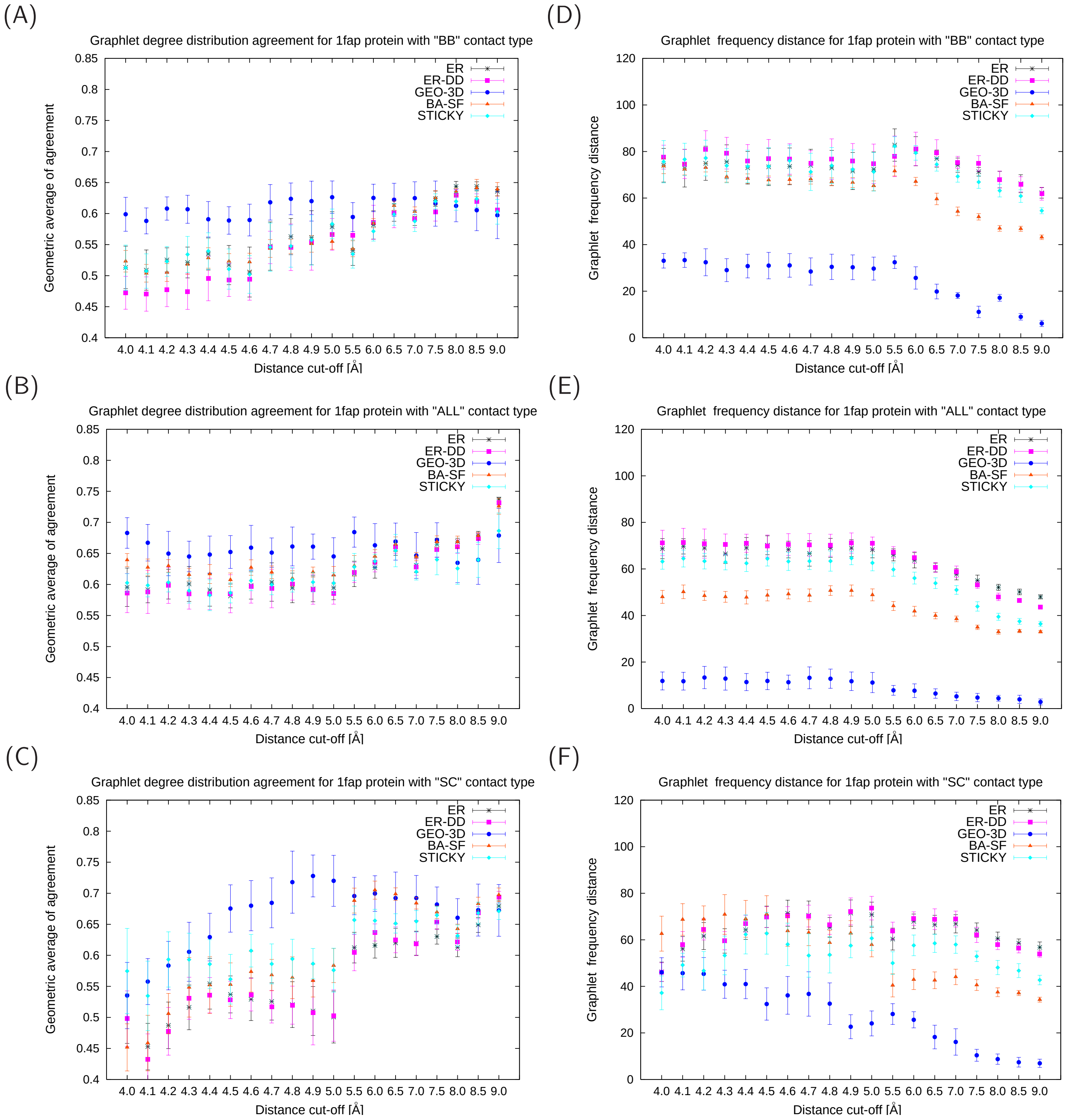

Figure S1.4 GDD-agreements and RGF-distances between model networks (ER, ER-DD, GEO-3D, SF-BA, and STICKY) and RIGs corresponding to 1fap protein that are constructed for each of the three contact types ("BB", "ALL", and "SC") and a series of distance cut-off values between 4.0 and 9.0 Angstroms: **A.** GDD-agreement for "BB" contact type. **B.** GDD-agreement for "ALL" contact type. **C.** GDD-agreement for "SC" contact type. **D.** RGF-distance for "BB" contact type. **E.** RGF-distance for "ALL" contact type. **F.** RGF-distance for "SC" contact type. The larger the GDD-agreement in panels A-C, the better the fit. The smaller the RGF-distance in panels D-F, the better the fit.

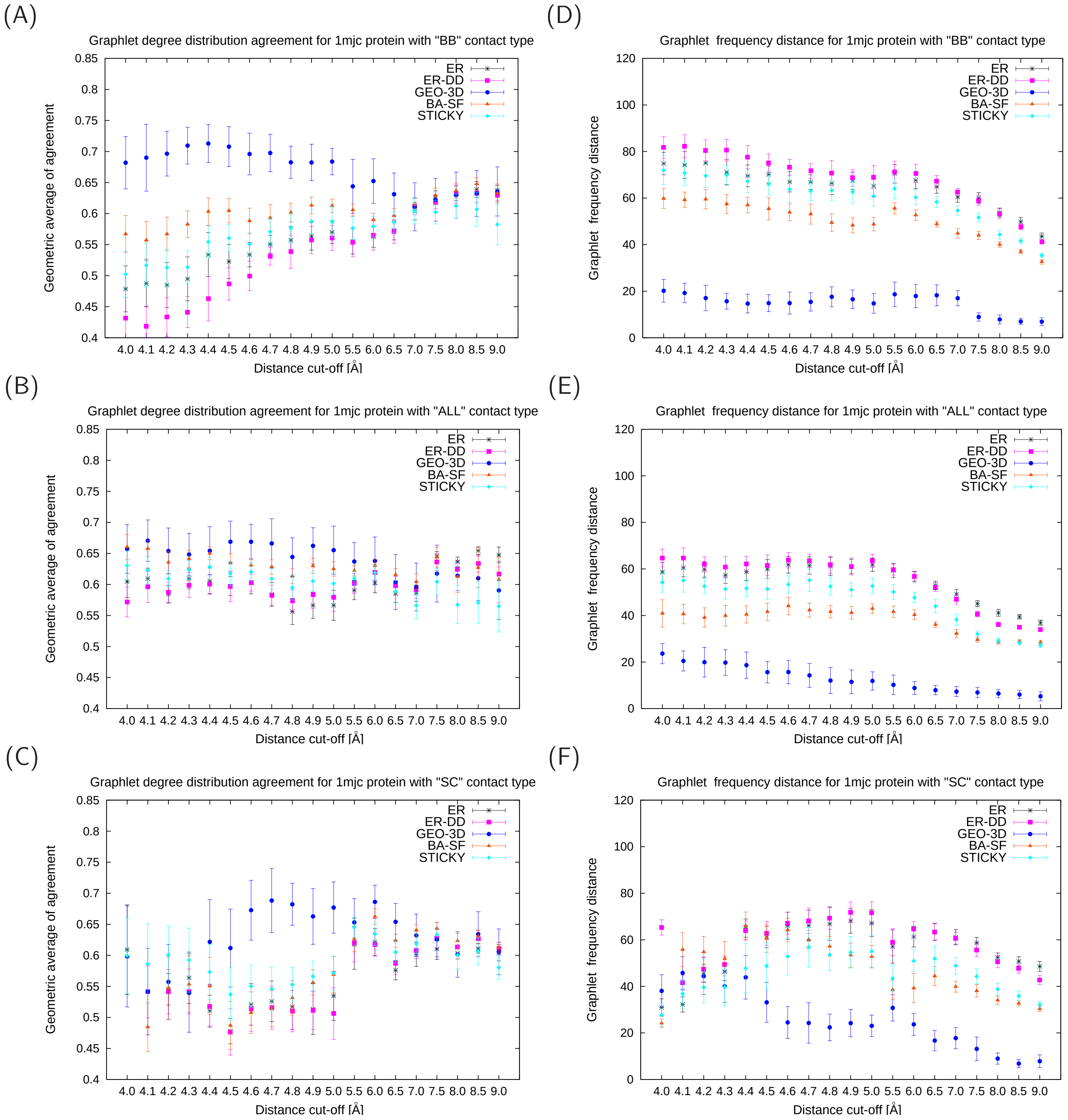

Figure S1.5 GDD-agreements and RGF-distances between model networks (ER, ER-DD, GEO-3D, SF-BA, and STICKY) and RIGs corresponding to 1mjc protein that are constructed for each of the three contact types ("BB", "ALL", and "SC") and a series of distance cut-off values between 4.0 and 9.0 Angstroms: **A.** GDD-agreement for "BB" contact type. **B.** GDD-agreement for "ALL" contact type. **C.** GDD-agreement for "SC" contact type. **D.** RGF-distance for "BB" contact type. **E.** RGF-distance for "ALL" contact type. **F.** RGF-distance for "SC" contact type. The larger the GDD-agreement in panels A-C, the better the fit. The smaller the RGF-distance in panels D-F, the better the fit.

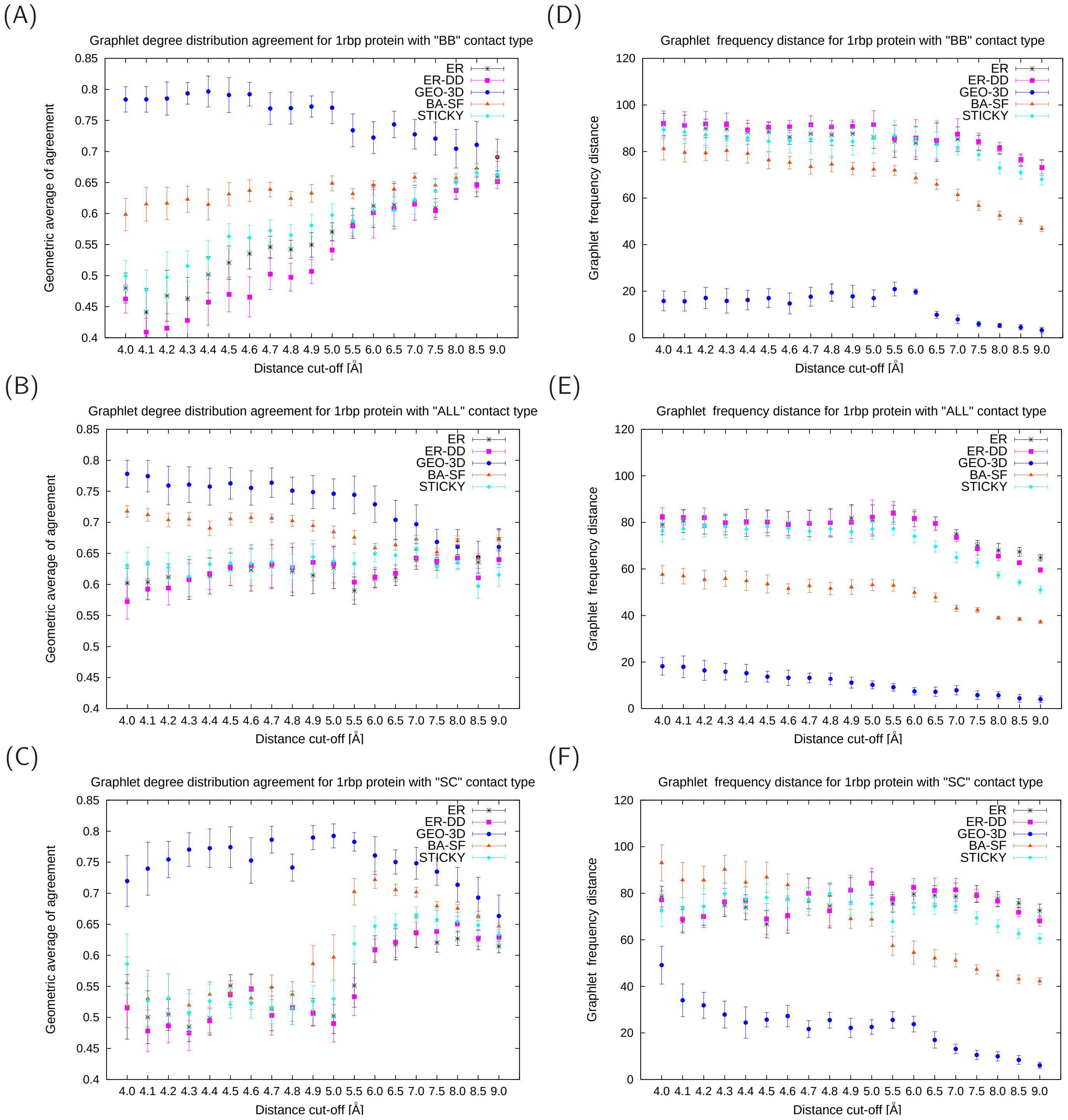

Figure S1.6 GDD-agreements and RGF-distances between model networks (ER, ER-DD, GEO-3D, SF-BA, and STICKY) and RIGs corresponding to 1rbp protein that are constructed for each of the three contact types ("BB", "ALL", and "SC") and a series of distance cut-off values between 4.0 and 9.0 Angstroms: **A.** GDD-agreement for "BB" contact type. **B.** GDD-agreement for "ALL" contact type. **C.** GDD-agreement for "SC" contact type. **D.** RGF-distance for "BB" contact type. **E.** RGF-distance for "ALL" contact type. **F.** RGF-distance for "SC" contact type. The larger the GDD-agreement in panels A-C, the better the fit. The smaller the RGF-distance in panels D-F, the better the fit.

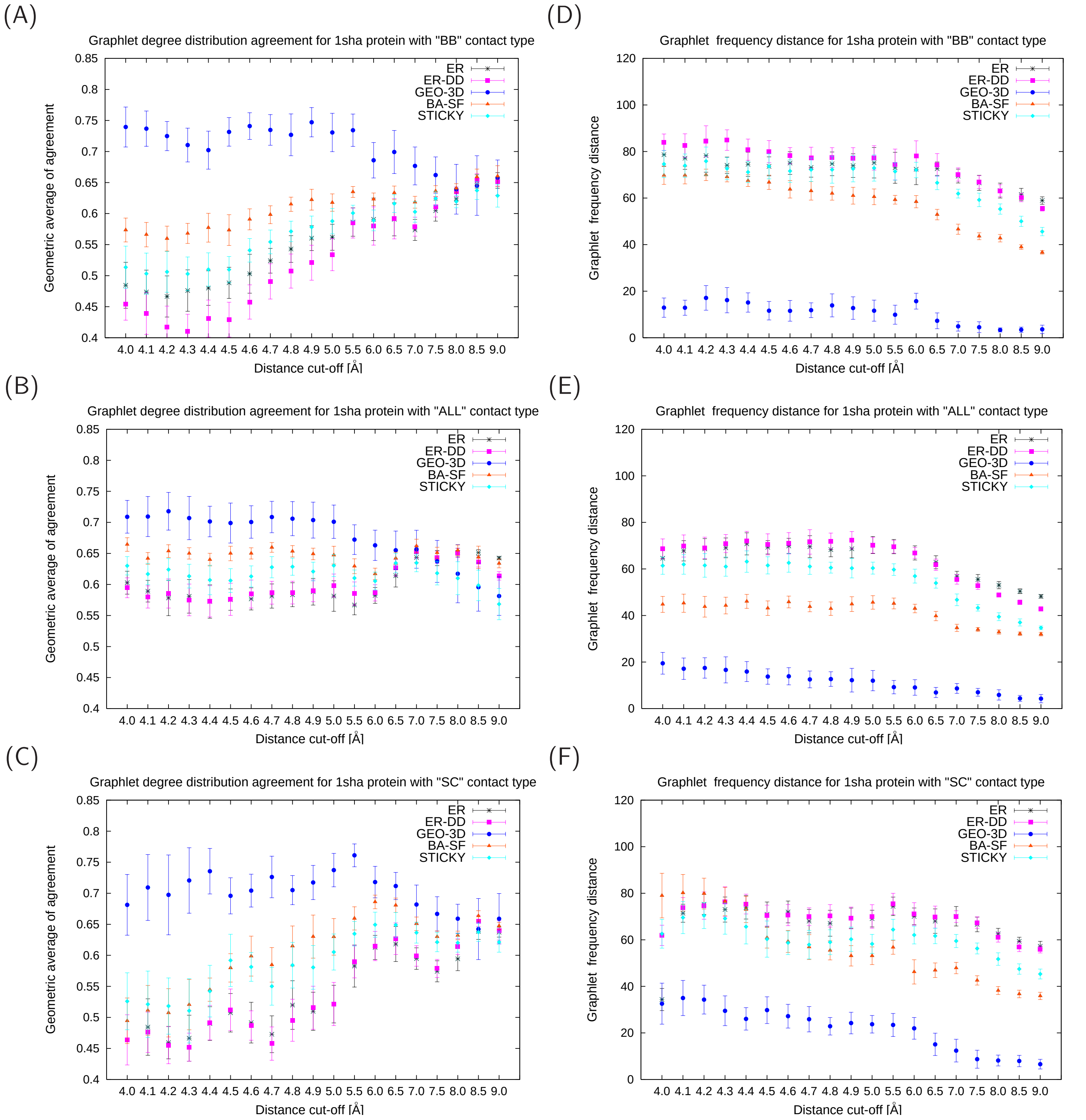

Figure S1.7 GDD-agreements and RGF-distances between model networks (ER, ER-DD, GEO-3D, SF-BA, and STICKY) and RIGs corresponding to 1sha protein that are constructed for each of the three contact types ("BB", "ALL", and "SC") and a series of distance cut-off values between 4.0 and 9.0 Angstroms: **A.** GDD-agreement for "BB" contact type. **B.** GDD-agreement for "ALL" contact type. **C.** GDD-agreement for "SC" contact type. **D.** RGF-distance for "BB" contact type. **E.** RGF-distance for "ALL" contact type. **F.** RGF-distance for "SC" contact type. The larger the GDD-agreement in panels A-C, the better the fit. The smaller the RGF-distance in panels D-F, the better the fit.

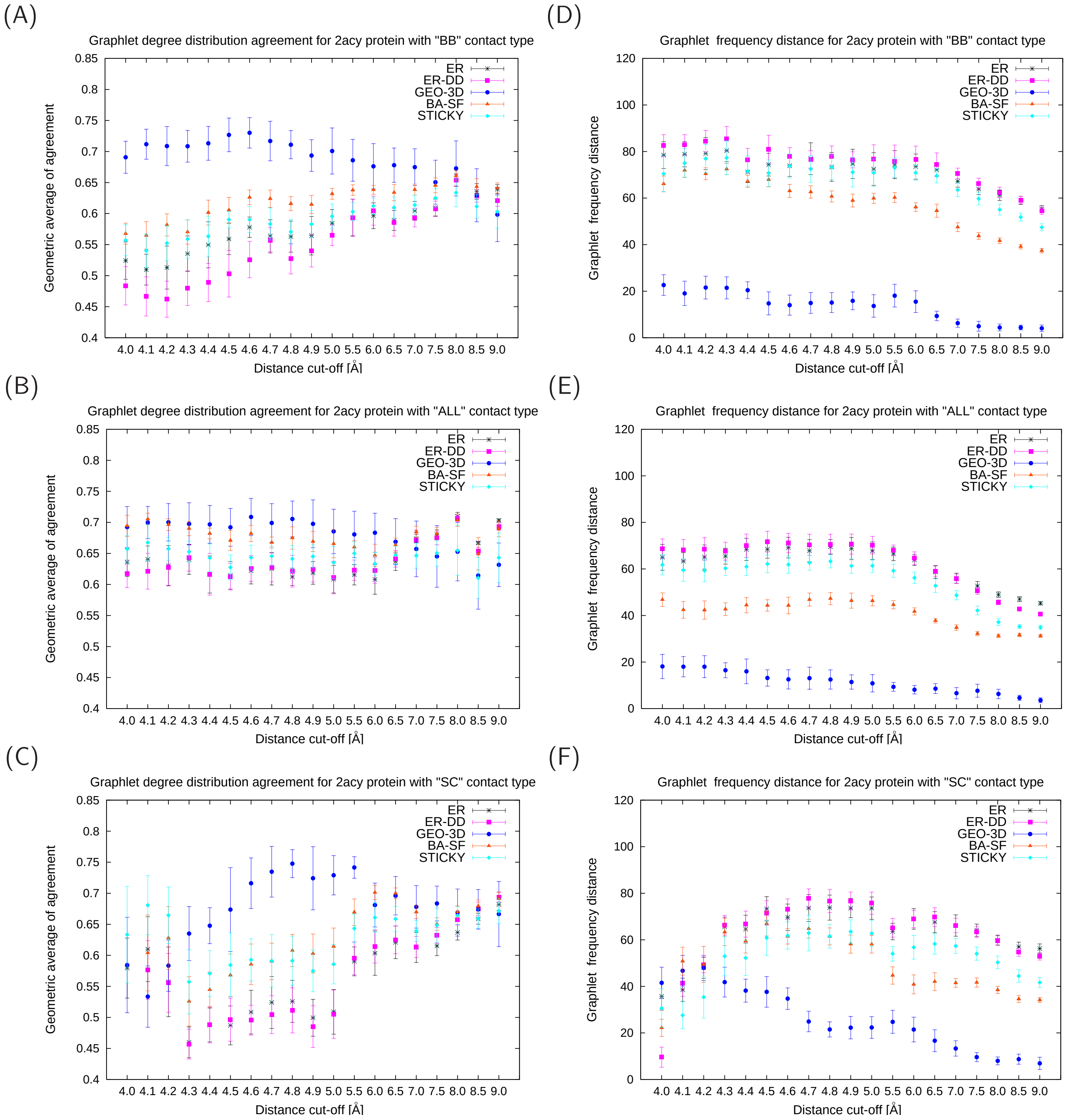

Figure S1.8 GDD-agreements and RGF-distances between model networks (ER, ER-DD, GEO-3D, SF-BA, and STICKY) and RIGs corresponding to 2acy protein that are constructed for each of the three contact types ("BB", "ALL", and "SC") and a series of distance cut-off values between 4.0 and 9.0 Angstroms: **A.** GDD-agreement for "BB" contact type. **B.** GDD-agreement for "ALL" contact type. **C.** GDD-agreement for "SC" contact type. **D.** RGF-distance for "BB" contact type. **E.** RGF-distance for "ALL" contact type. **F.** RGF-distance for "SC" contact type. The larger the GDD-agreement in panels A-C, the better the fit. The smaller the RGF-distance in panels D-F, the better the fit.

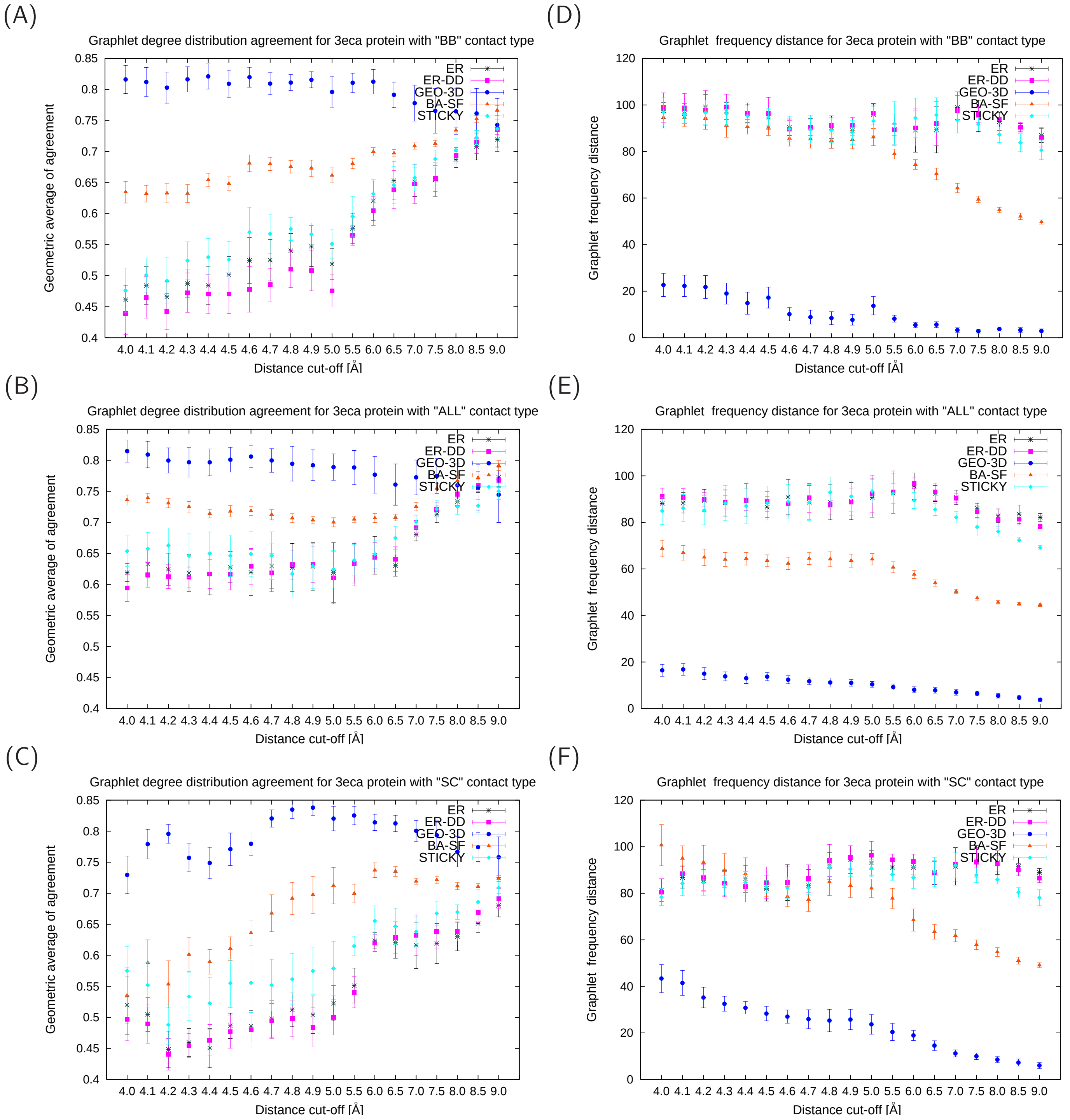

Figure S1.9 GDD-agreements and RGF-distances between model networks (ER, ER-DD, GEO-3D, SF-BA, and STICKY) and RIGs corresponding to 3eca protein that are constructed for each of the three contact types ("BB", "ALL", and "SC") and a series of distance cut-off values between 4.0 and 9.0 Angstroms: **A.** GDD-agreement for "BB" contact type. **B.** GDD-agreement for "ALL" contact type. **C.** GDD-agreement for "SC" contact type. **D.** RGF-distance for "BB" contact type. **E.** RGF-distance for "ALL" contact type. **F.** RGF-distance for "SC" contact type. The larger the GDD-agreement in panels A-C, the better the fit. The smaller the RGF-distance in panels D-F, the better the fit.

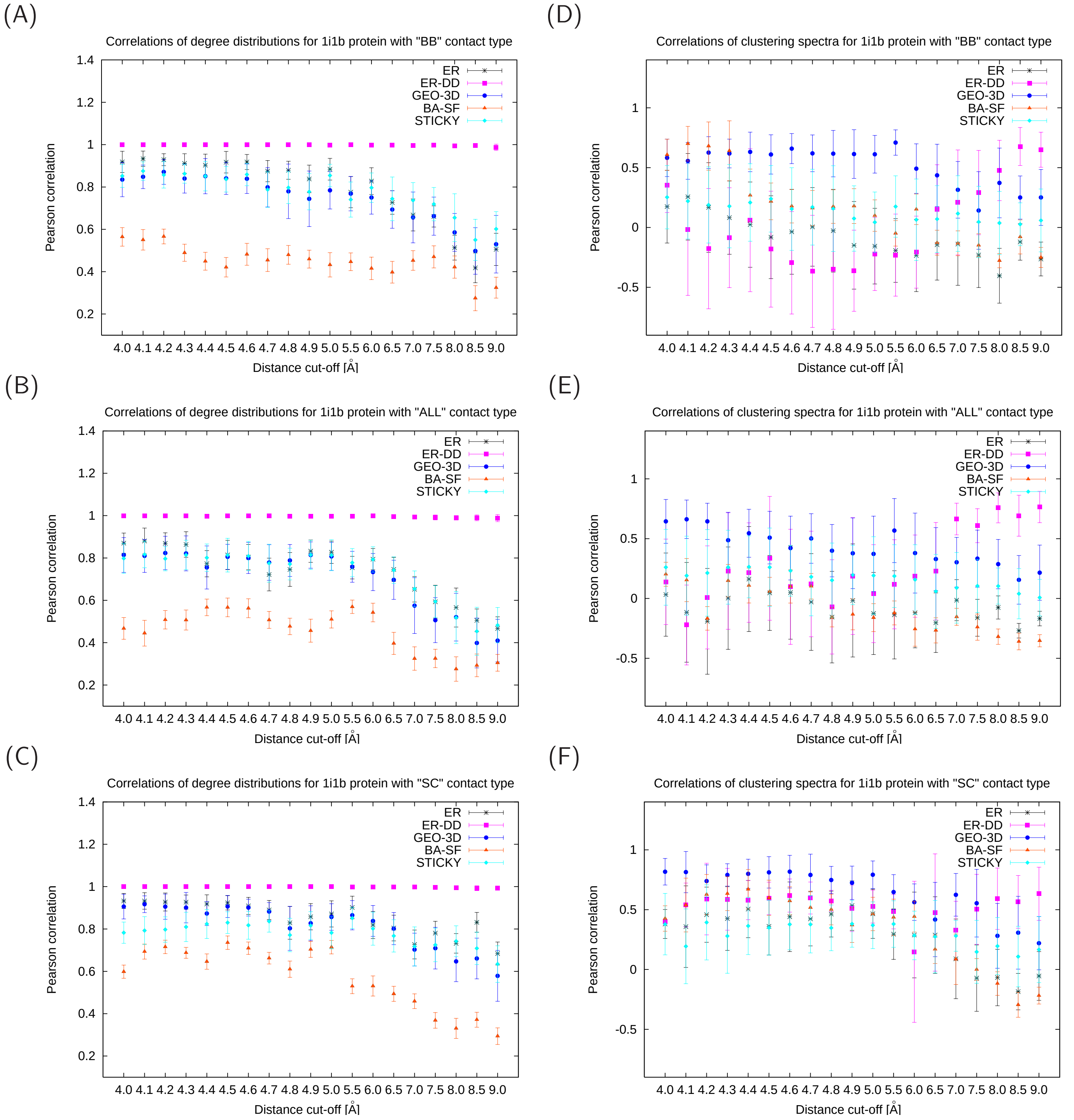

Figure S1.10 The Pearson correlation coefficients of degree distributions and clustering spectra of model networks (ER, ER-DD, GEO-3D, SF-BA, and STICKY) and RIGs corresponding to 1i1b protein that are constructed for each of the three contact types (“BB”, “ALL”, and “SC”) and a series of distance cut-off values between 4.0 and 9.0 Angstroms: **A.** degree distribution for “BB” contact type. **B.** degree distribution for “ALL” contact type. **C.** degree distribution for “SC” contact type. **D.** clustering spectrum for “BB” contact type. **E.** clustering spectrum for “ALL” contact type. **F.** clustering spectrum for “SC” contact type.

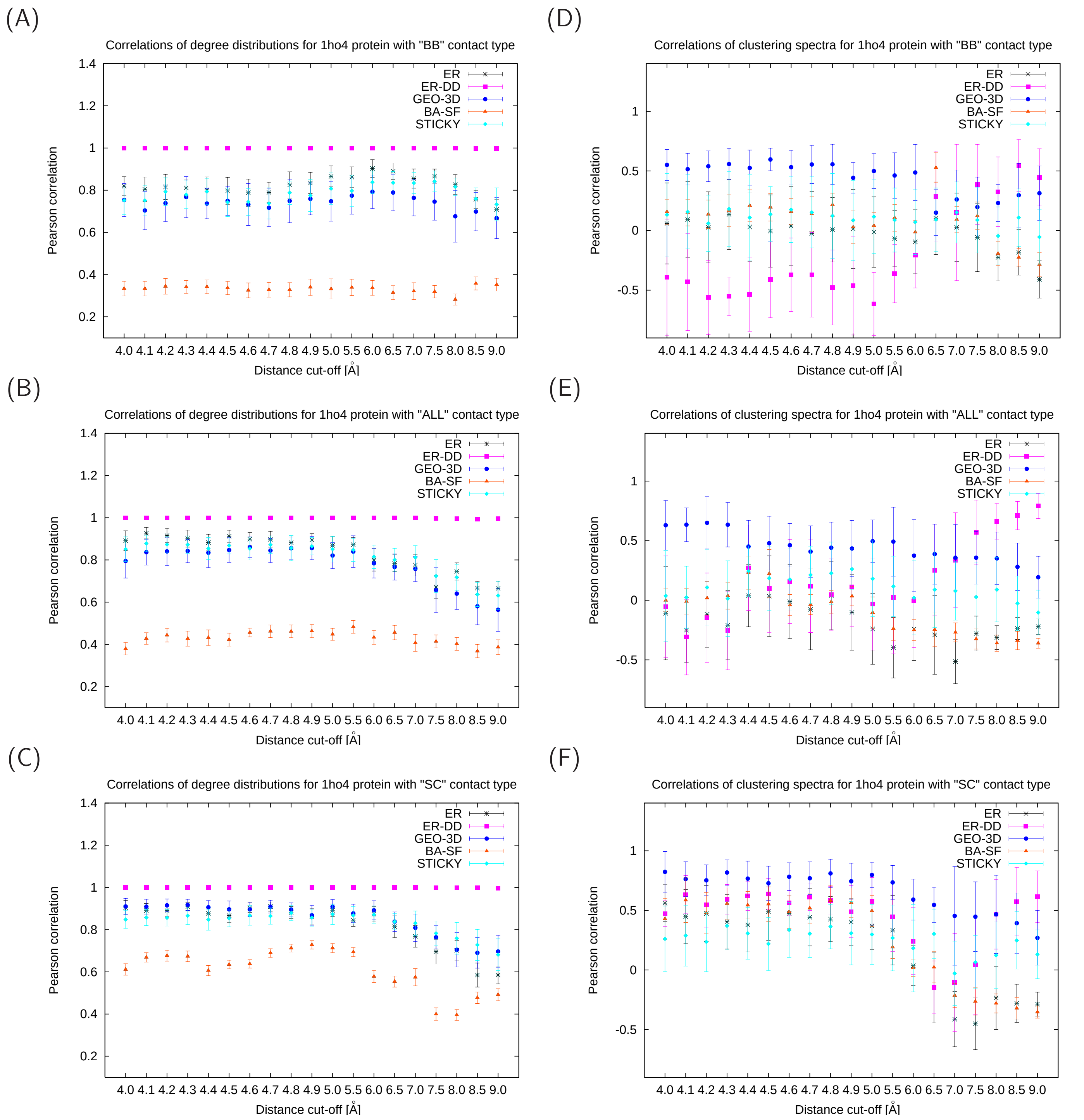

Figure S1.11 The Pearson correlation coefficients of degree distributions and clustering spectra of model networks (ER, ER-DD, GEO-3D, SF-BA, and STICKY) and RIGs corresponding to 1ho4 protein that are constructed for each of the three contact types ("BB", "ALL", and "SC") and a series of distance cut-off values between 4.0 and 9.0 Angstroms: **A.** degree distribution for "BB" contact type. **B.** degree distribution for "ALL" contact type. **C.** degree distribution for "SC" contact type. **D.** clustering spectrum for "BB" contact type. **E.** clustering spectrum for "ALL" contact type. **F.** clustering spectrum for "SC" contact type.

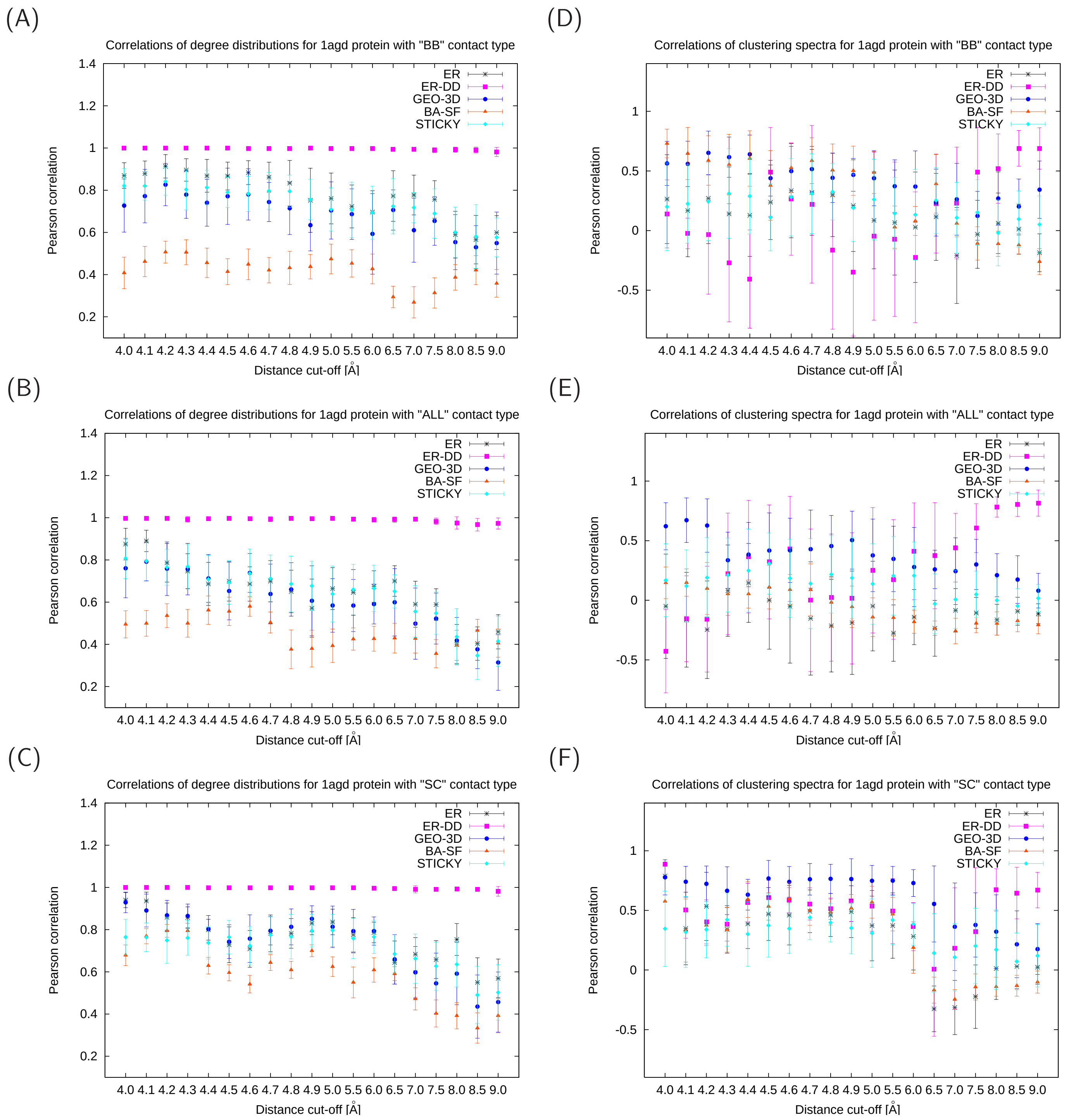

Figure S1.12 The Pearson correlation coefficients of degree distributions and clustering spectra of model networks (ER, ER-DD, GEO-3D, SF-BA, and STICKY) and RIGs corresponding to 1agd protein that are constructed for each of the three contact types (“BB”, “ALL”, and “SC”) and a series of distance cut-off values between 4.0 and 9.0 Angstroms: **A.** degree distribution for “BB” contact type. **B.** degree distribution for “ALL” contact type. **C.** degree distribution for “SC” contact type. **D.** clustering spectrum for “BB” contact type. **E.** clustering spectrum for “ALL” contact type. **F.** clustering spectrum for “SC” contact type.

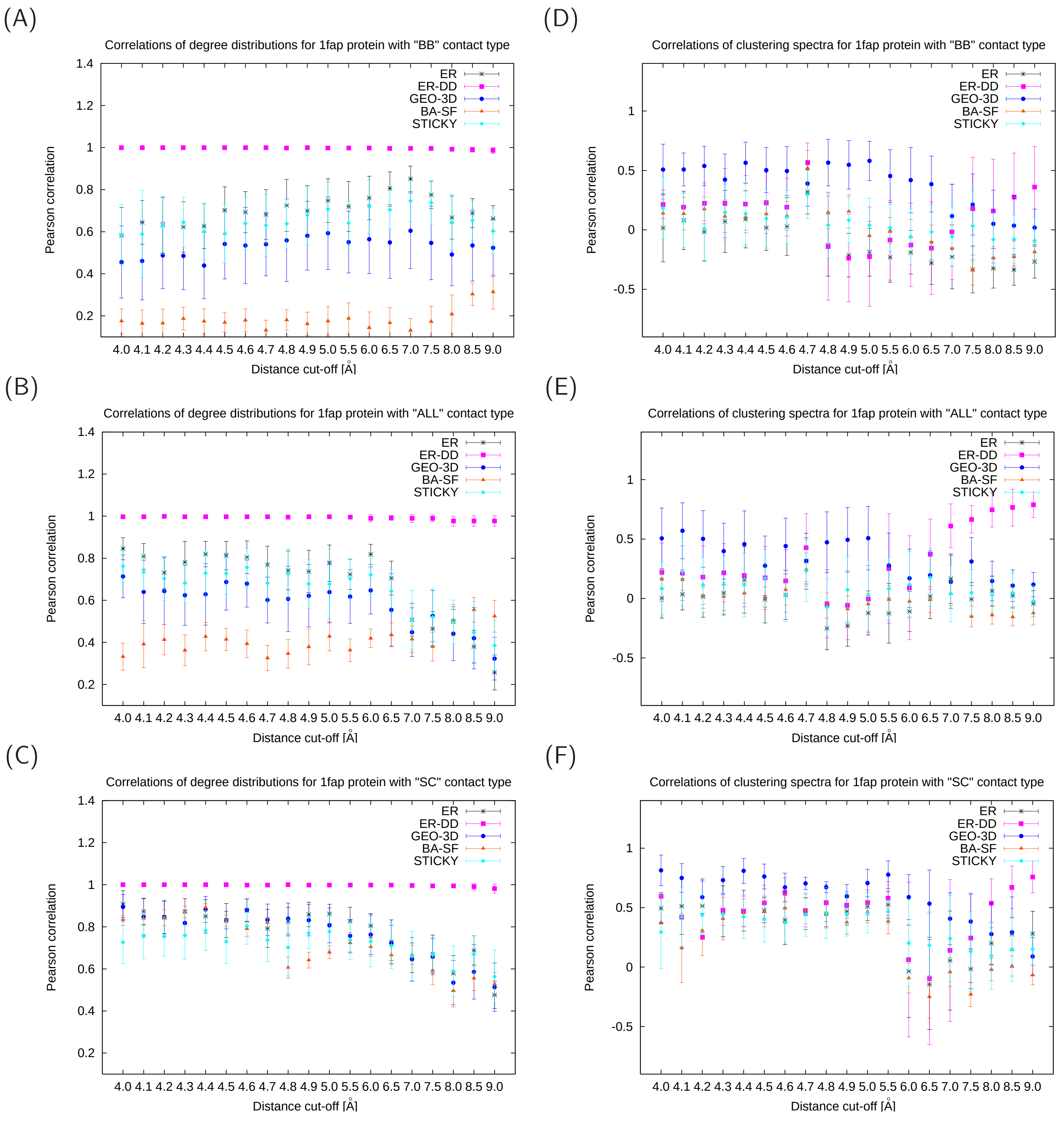

Figure S1.13 The Pearson correlation coefficients of degree distributions and clustering spectra of model networks (ER, ER-DD, GEO-3D, SF-BA, and STICKY) and RIGs corresponding to 1fap protein that are constructed for each of the three contact types (“BB”, “ALL”, and “SC”) and a series of distance cut-off values between 4.0 and 9.0 Angstroms: **A.** degree distribution for “BB” contact type. **B.** degree distribution for “ALL” contact type. **C.** degree distribution for “SC” contact type. **D.** clustering spectrum for “BB” contact type. **E.** clustering spectrum for “ALL” contact type. **F.** clustering spectrum for “SC” contact type.

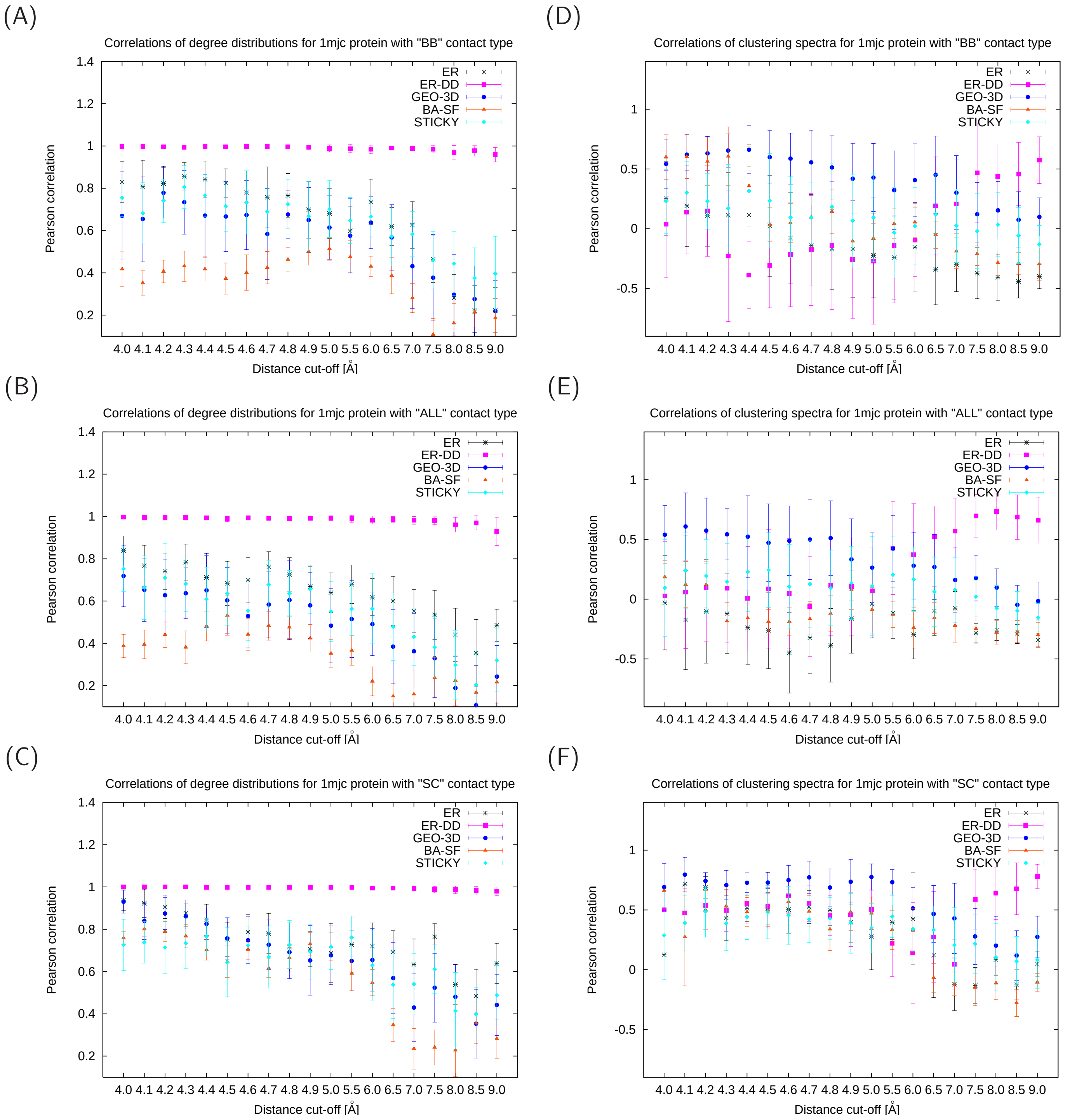

Figure S1.14 The Pearson correlation coefficients of degree distributions and clustering spectra of model networks (ER, ER-DD, GEO-3D, SF-BA, and STICKY) and RIGs corresponding to 1mjc protein that are constructed for each of the three contact types (“BB”, “ALL”, and “SC”) and a series of distance cut-off values between 4.0 and 9.0 Angstroms: **A.** degree distribution for “BB” contact type. **B.** degree distribution for “ALL” contact type. **C.** degree distribution for “SC” contact type. **D.** clustering spectrum for “BB” contact type. **E.** clustering spectrum for “ALL” contact type. **F.** clustering spectrum for “SC” contact type.

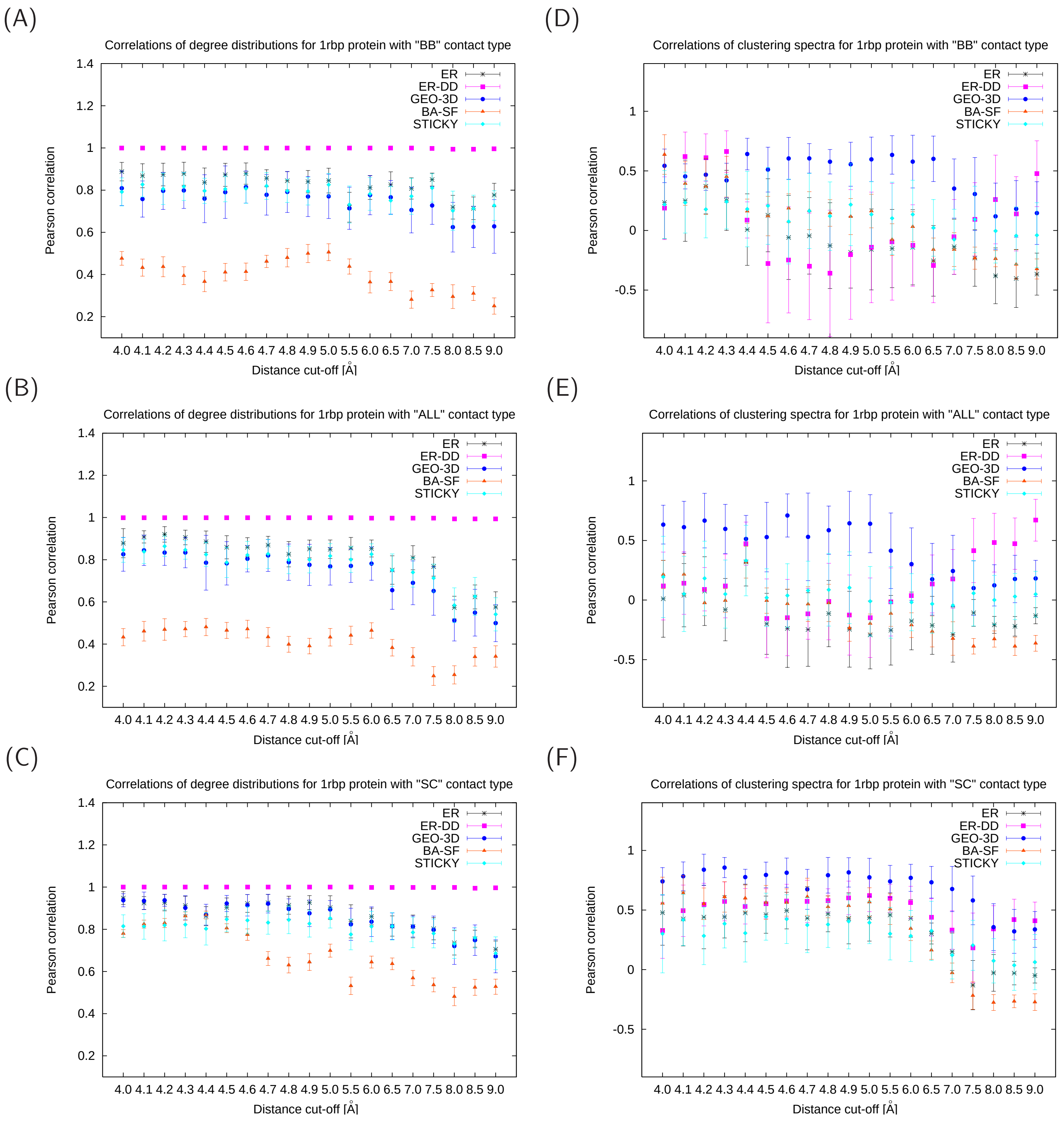

Figure S1.15 The Pearson correlation coefficients of degree distributions and clustering spectra of model networks (ER, ER-DD, GEO-3D, SF-BA, and STICKY) and RIGs corresponding to 1rbp protein that are constructed for each of the three contact types (“BB”, “ALL”, and “SC”) and a series of distance cut-off values between 4.0 and 9.0 Angstroms: **A.** degree distribution for “BB” contact type. **B.** degree distribution for “ALL” contact type. **C.** degree distribution for “SC” contact type. **D.** clustering spectrum for “BB” contact type. **E.** clustering spectrum for “ALL” contact type. **F.** clustering spectrum for “SC” contact type.

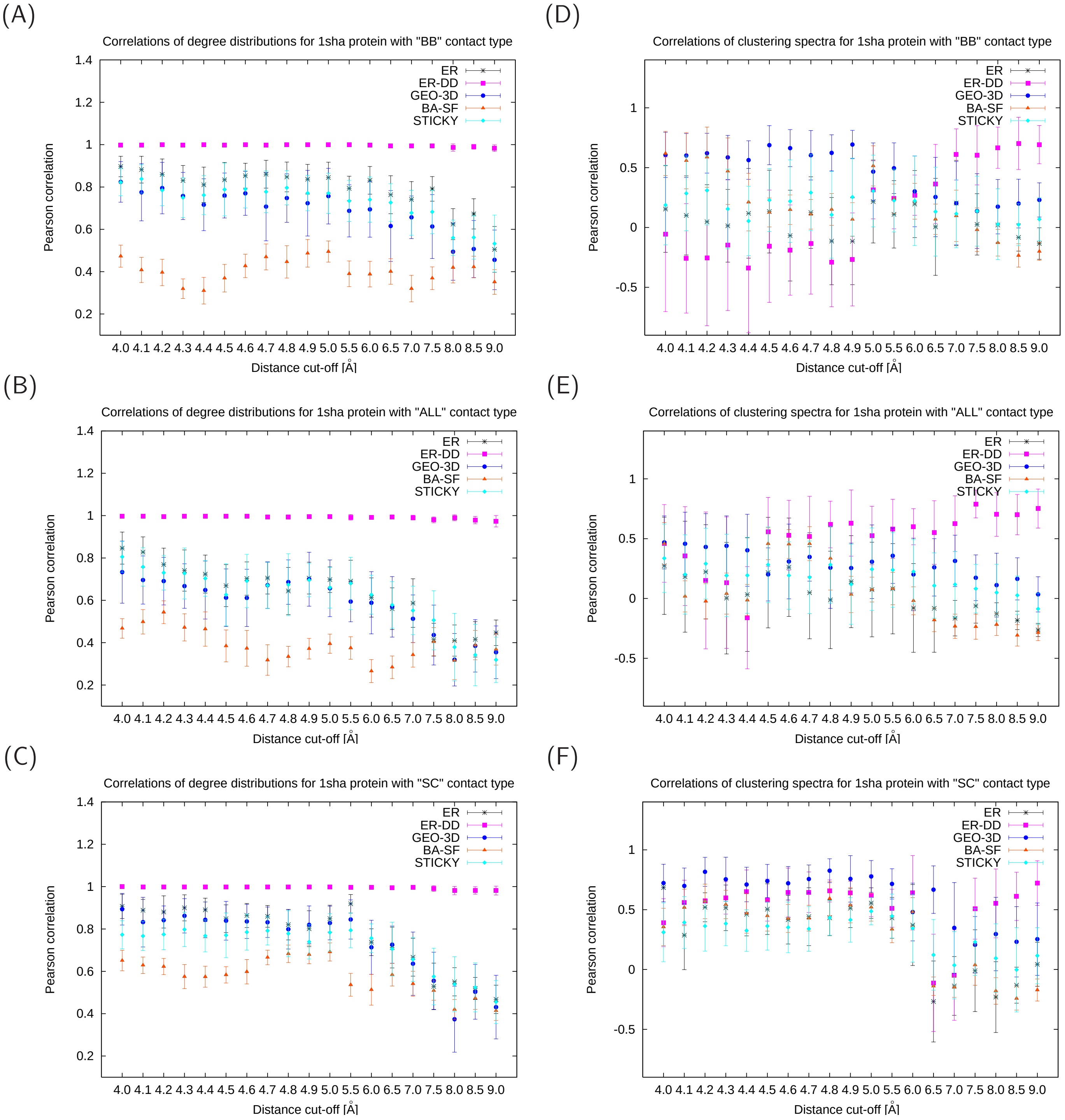

Figure S1.16 The Pearson correlation coefficients of degree distributions and clustering spectra of model networks (ER, ER-DD, GEO-3D, SF-BA, and STICKY) and RIGs corresponding to 1sha protein that are constructed for each of the three contact types ("BB", "ALL", and "SC") and a series of distance cut-off values between 4.0 and 9.0 Angstroms: **A.** degree distribution for "BB" contact type. **B.** degree distribution for "ALL" contact type. **C.** degree distribution for "SC" contact type. **D.** clustering spectrum for "BB" contact type. **E.** clustering spectrum for "ALL" contact type. **F.** clustering spectrum for "SC" contact type.

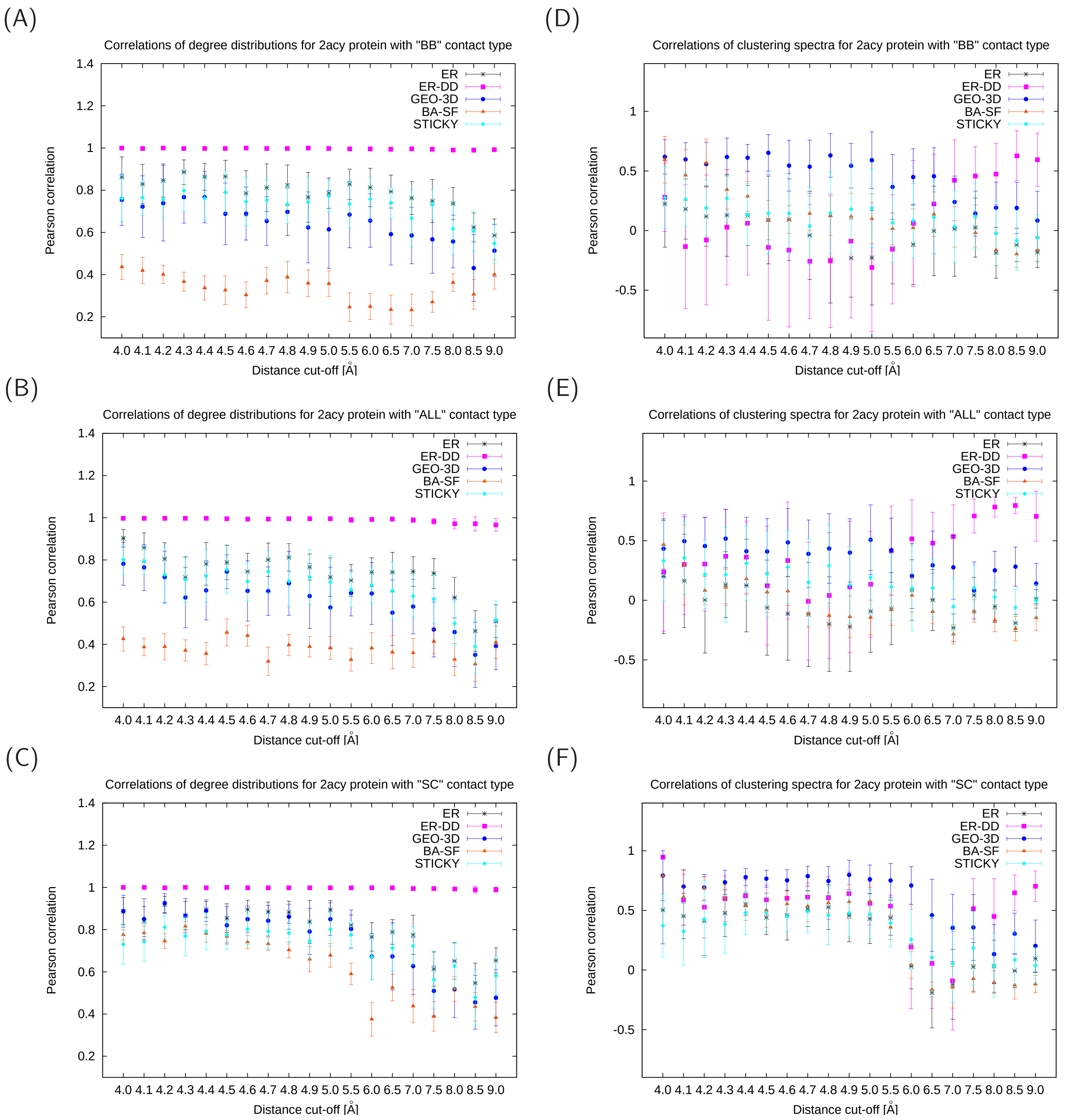

Figure S1.17 The Pearson correlation coefficients of degree distributions and clustering spectra of model networks (ER, ER-DD, GEO-3D, SF-BA, and STICKY) and RIGs corresponding to 2acy protein that are constructed for each of the three contact types ("BB", "ALL", and "SC") and a series of distance cut-off values between 4.0 and 9.0 Angstroms: **A.** degree distribution for "BB" contact type. **B.** degree distribution for "ALL" contact type. **C.** degree distribution for "SC" contact type. **D.** clustering spectrum for "BB" contact type. **E.** clustering spectrum for "ALL" contact type. **F.** clustering spectrum for "SC" contact type.

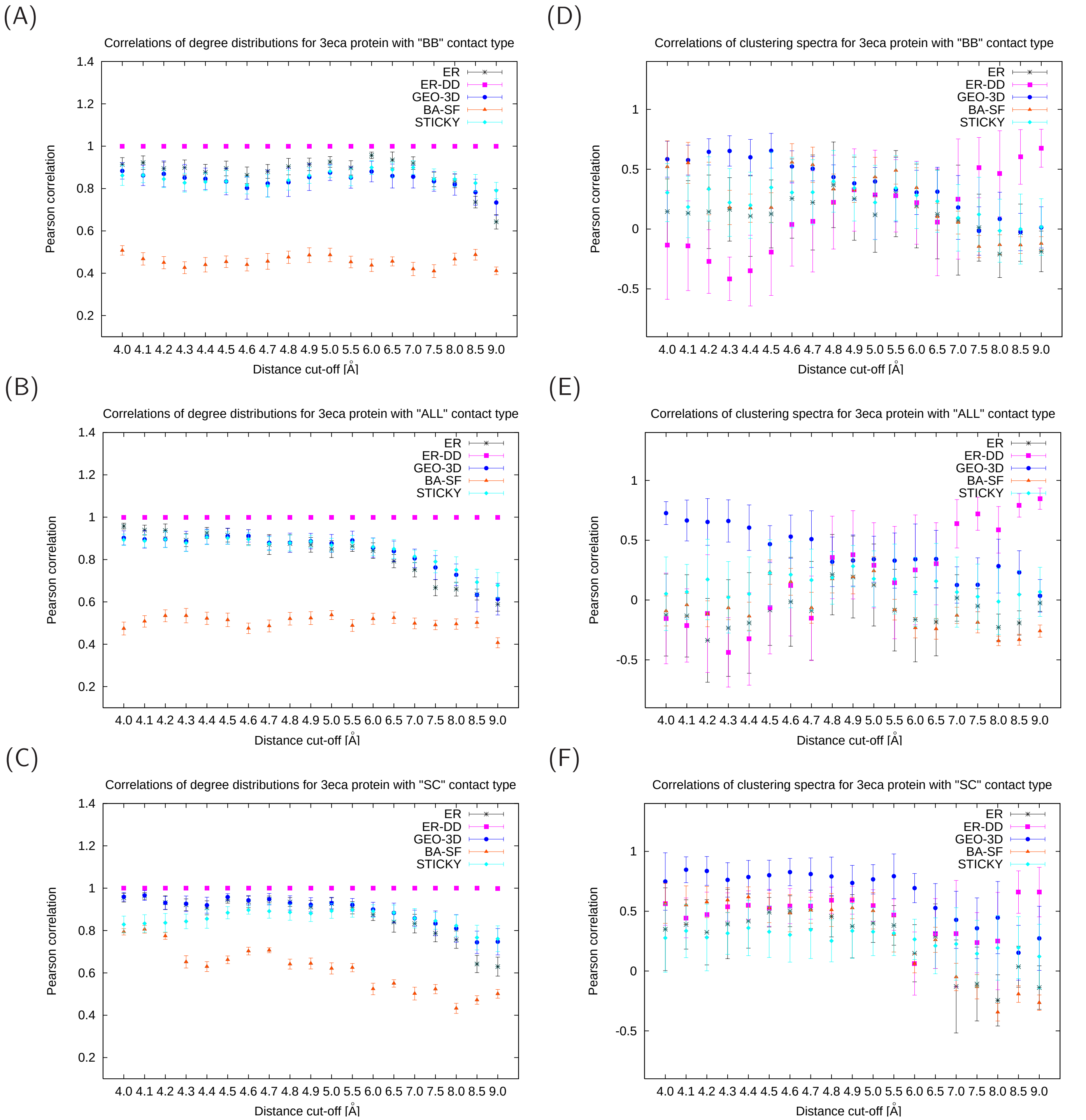

Figure S1.18 The Pearson correlation coefficients of degree distributions and clustering spectra of model networks (ER, ER-DD, GEO-3D, SF-BA, and STICKY) and RIGs corresponding to 3eca protein that are constructed for each of the three contact types ("BB", "ALL", and "SC") and a series of distance cut-off values between 4.0 and 9.0 Angstroms: **A.** degree distribution for "BB" contact type. **B.** degree distribution for "ALL" contact type. **C.** degree distribution for "SC" contact type. **D.** clustering spectrum for "BB" contact type. **E.** clustering spectrum for "ALL" contact type. **F.** clustering spectrum for "SC" contact type.

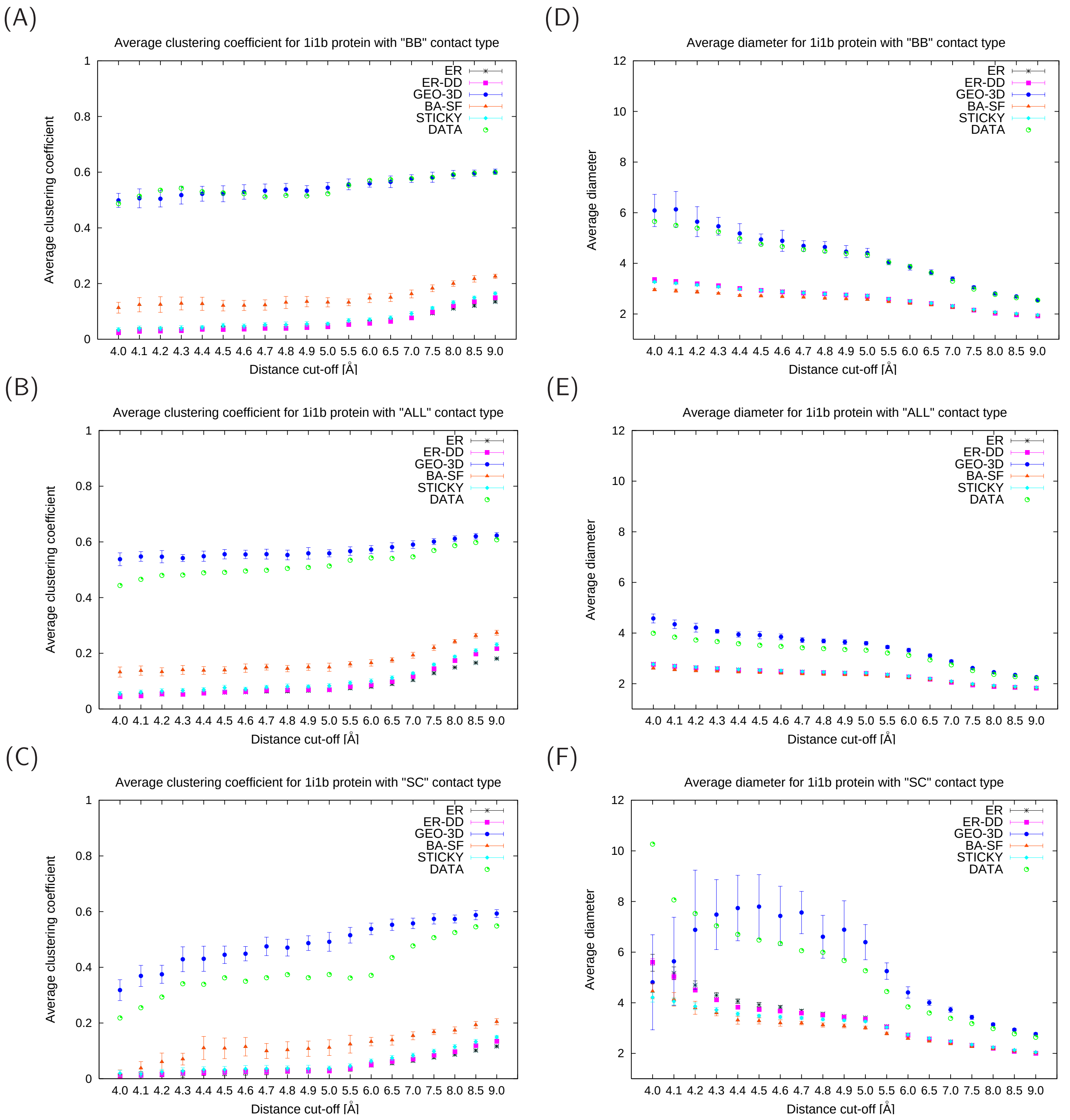

Figure S1.19 The agreements of average clustering coefficients and diameters of model networks (ER, ER-DD, GEO-3D, SF-BA, and STICKY) and RIGs corresponding to 1i1b protein that are constructed for each of the three contact types ("BB", "ALL", and "SC") and a series of distance cut-off values between 4.0 and 9.0 Angstroms: **A.** average clustering coefficients for "BB" contact type. **B.** average clustering coefficients for "ALL" contact type. **C.** average clustering coefficients for "SC" contact type. **D.** average diameters for "BB" contact type. **E.** average diameters for "ALL" contact type. **F.** average diameters for "SC" contact type.

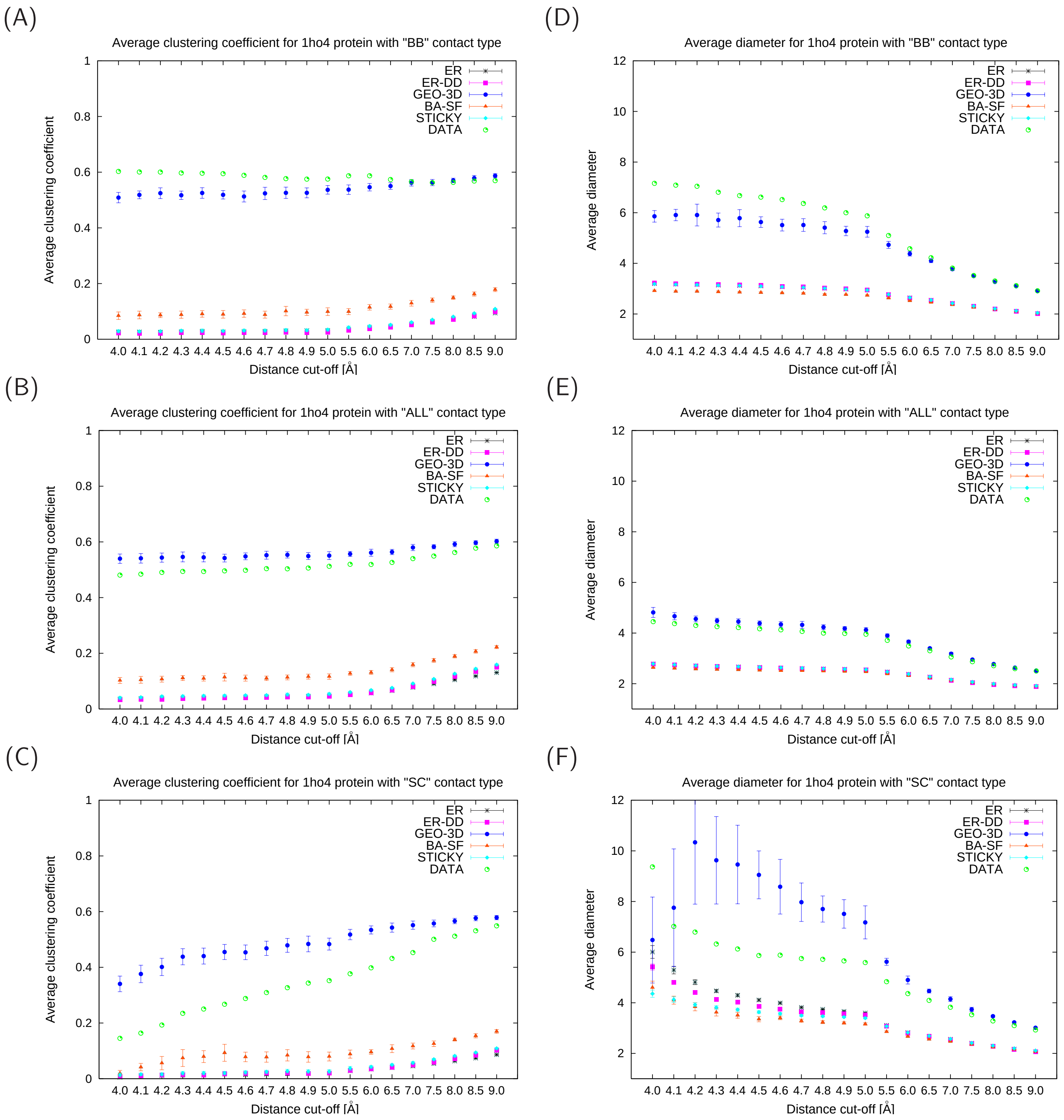

Figure S1.20 The agreements of average clustering coefficients and diameters of model networks (ER, ER-DD, GEO-3D, SF-BA, and STICKY) and RIGs corresponding to 1ho4 protein that are constructed for each of the three contact types ("BB", "ALL", and "SC") and a series of distance cut-off values between 4.0 and 9.0 Angstroms: **A.** average clustering coefficients for "BB" contact type. **B.** average clustering coefficients for "ALL" contact type. **C.** average clustering coefficients for "SC" contact type. **D.** average diameters for "BB" contact type. **E.** average diameters for "ALL" contact type. **F.** average diameters for "SC" contact type.

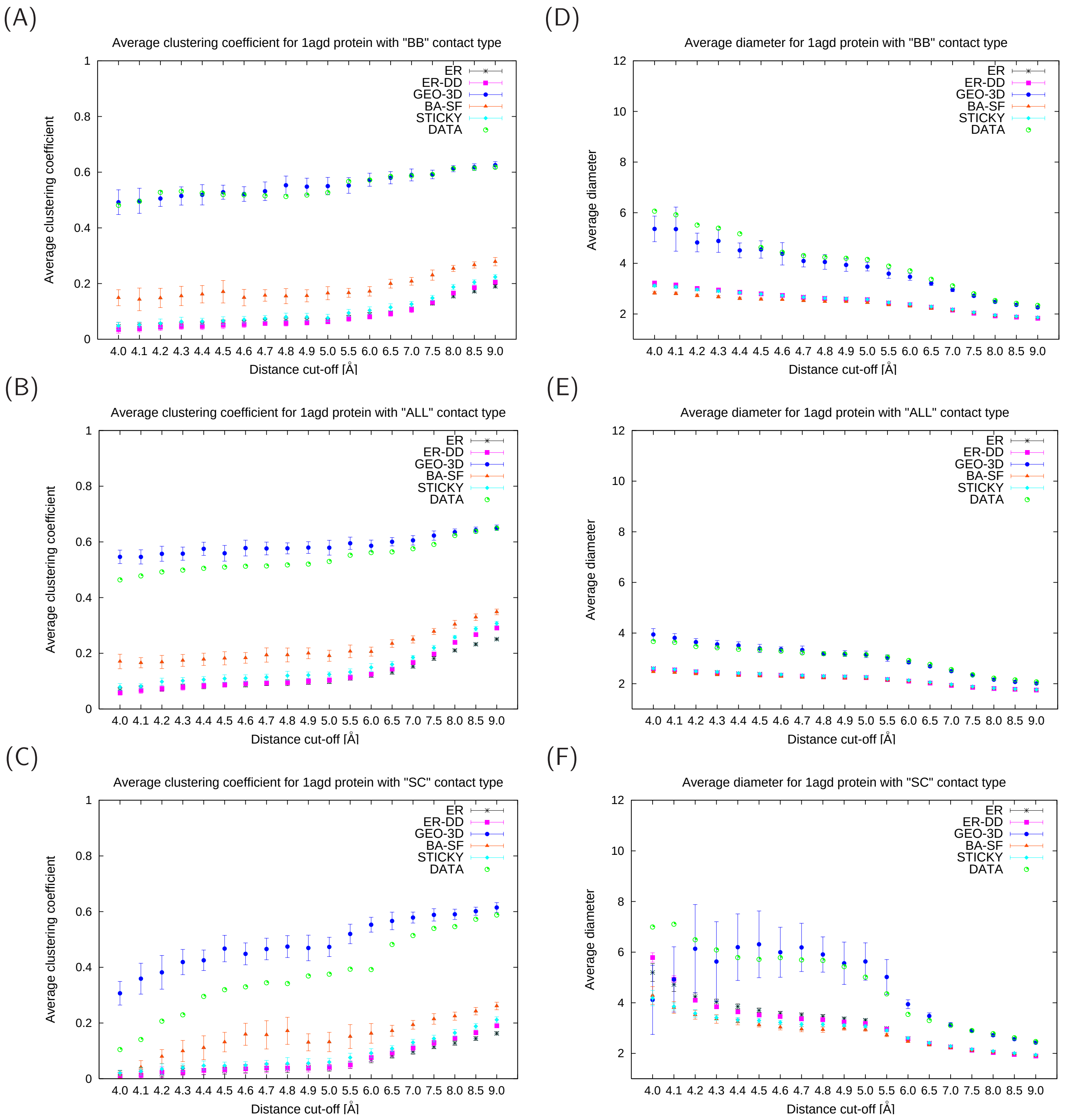

Figure S1.21 The agreements of average clustering coefficients and diameters of model networks (ER, ER-DD, GEO-3D, SF-BA, and STICKY) and RIGs corresponding to 1agd protein that are constructed for each of the three contact types (“BB”, “ALL”, and “SC”) and a series of distance cut-off values between 4.0 and 9.0 Angstroms: **A.** average clustering coefficients for “BB” contact type. **B.** average clustering coefficients for “ALL” contact type. **C.** average clustering coefficients for “SC” contact type. **D.** average diameters for “BB” contact type. **E.** average diameters for “ALL” contact type. **F.** average diameters for “SC” contact type.

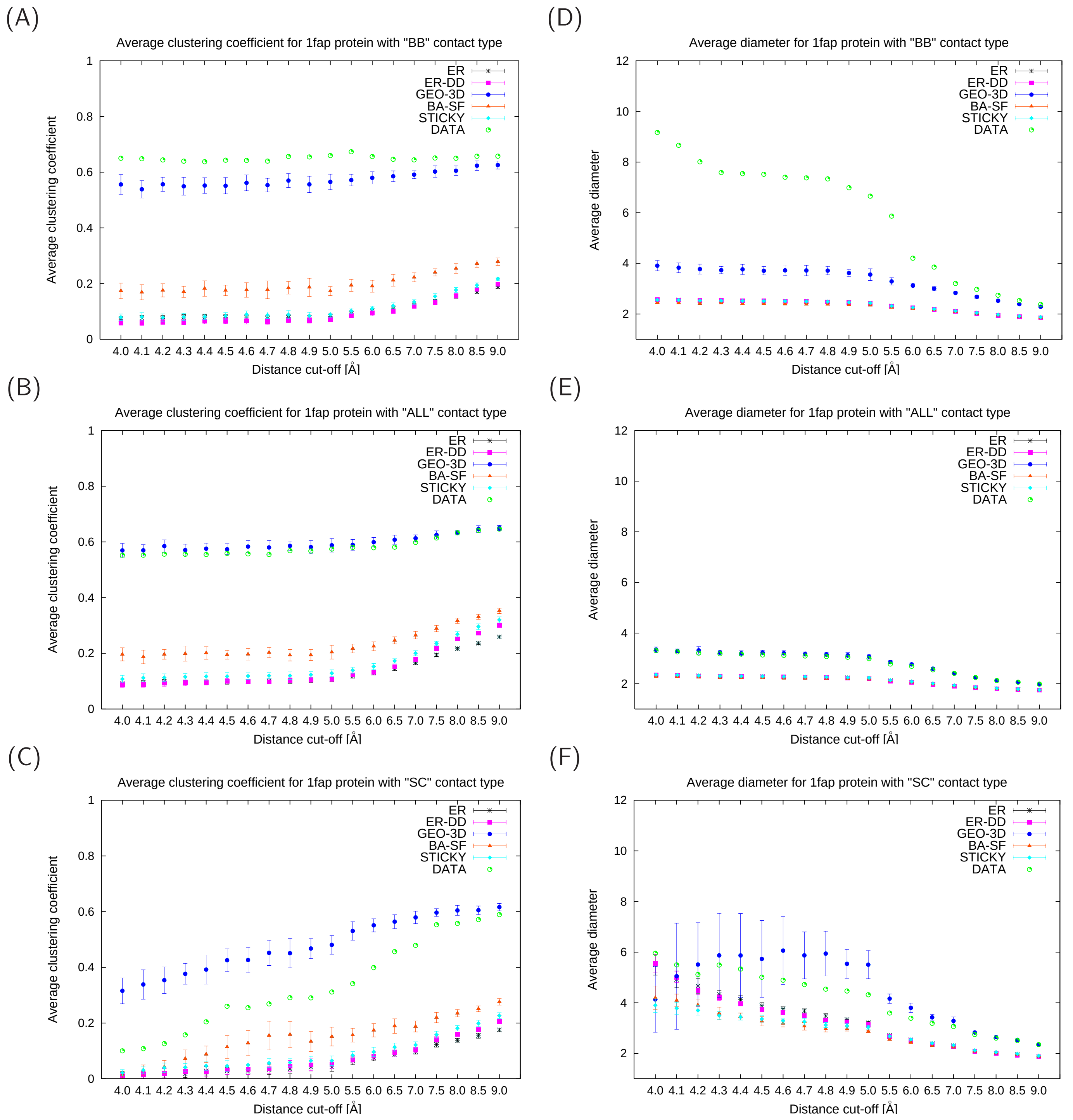

Figure S1.22 The agreements of average clustering coefficients and diameters of model networks (ER, ER-DD, GEO-3D, SF-BA, and STICKY) and RIGs corresponding to 1fap protein that are constructed for each of the three contact types ("BB", "ALL", and "SC") and a series of distance cut-off values between 4.0 and 9.0 Angstroms: **A.** average clustering coefficients for "BB" contact type. **B.** average clustering coefficients for "ALL" contact type. **C.** average clustering coefficients for "SC" contact type. **D.** average diameters for "BB" contact type. **E.** average diameters for "ALL" contact type. **F.** average diameters for "SC" contact type.

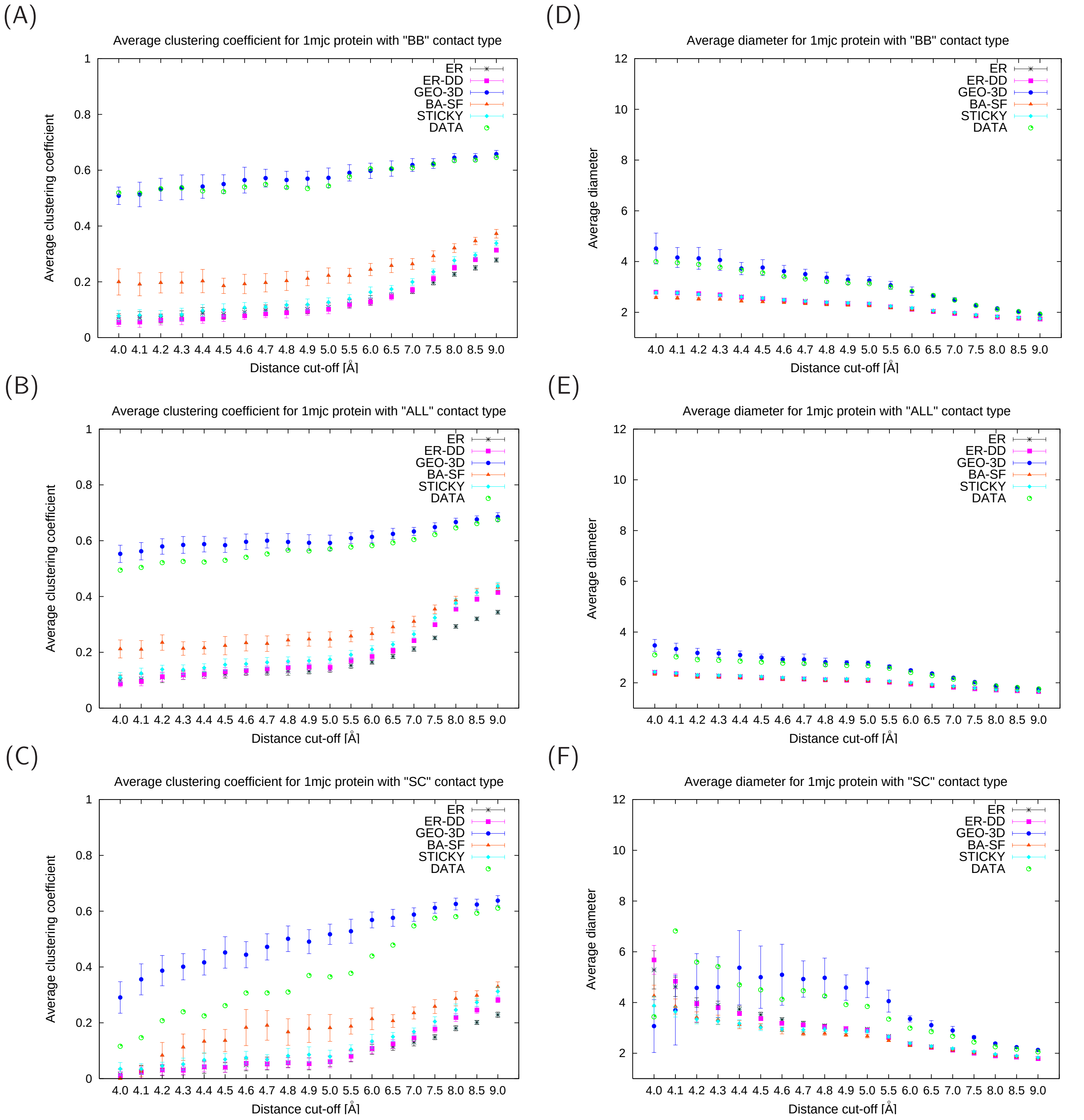

Figure S1.23 The agreements of average clustering coefficients and diameters of model networks (ER, ER-DD, GEO-3D, SF-BA, and STICKY) and RIGs corresponding to 1mjc protein that are constructed for each of the three contact types (“BB”, “ALL”, and “SC”) and a series of distance cut-off values between 4.0 and 9.0 Angstroms: **A.** average clustering coefficients for “BB” contact type. **B.** average clustering coefficients for “ALL” contact type. **C.** average clustering coefficients for “SC” contact type. **D.** average diameters for “BB” contact type. **E.** average diameters for “ALL” contact type. **F.** average diameters for “SC” contact type.

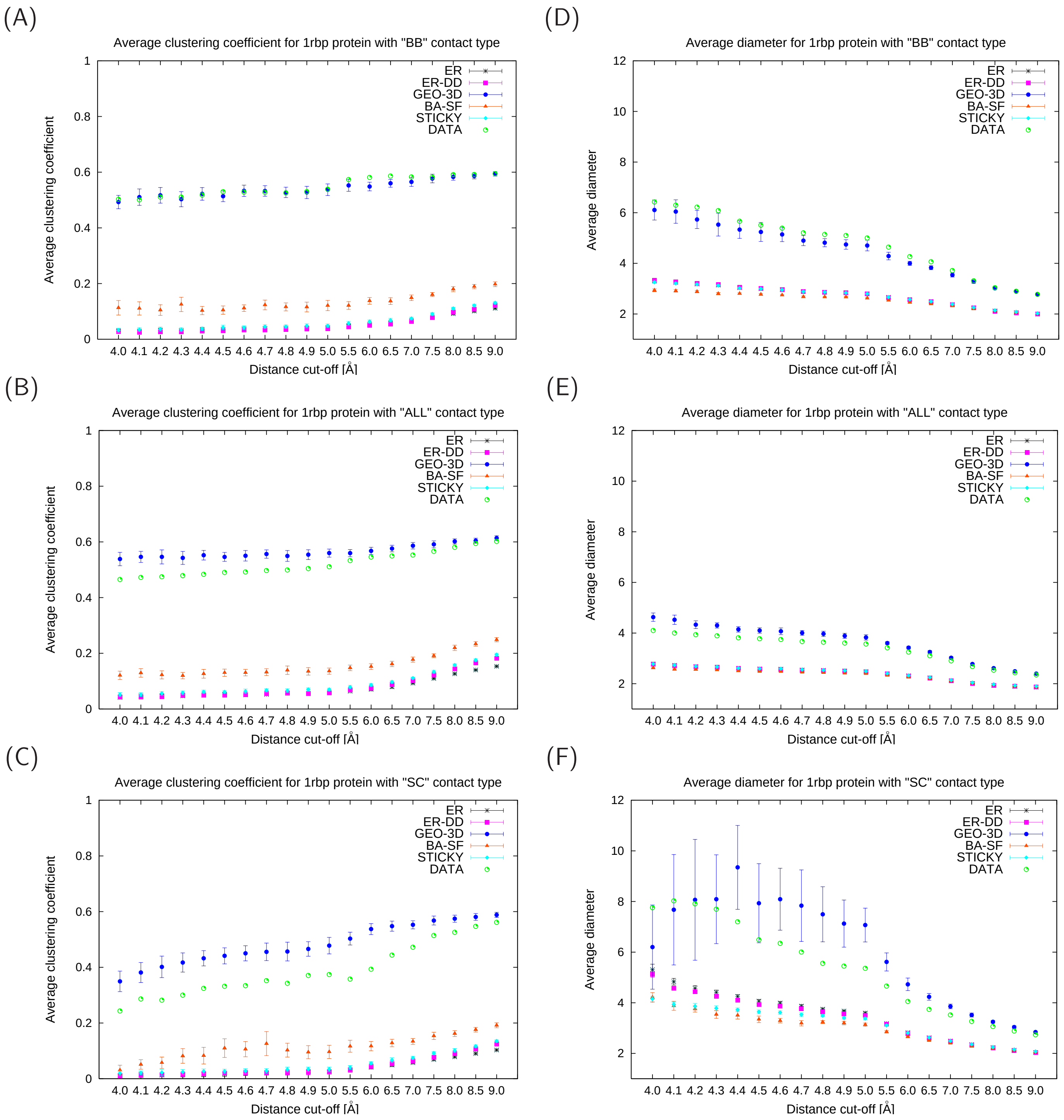

Figure S1.24 The agreements of average clustering coefficients and diameters of model networks (ER, ER-DD, GEO-3D, SF-BA, and STICKY) and RIGs corresponding to 1rbp protein that are constructed for each of the three contact types ("BB", "ALL", and "SC") and a series of distance cut-off values between 4.0 and 9.0 Angstroms: **A.** average clustering coefficients for "BB" contact type. **B.** average clustering coefficients for "ALL" contact type. **C.** average clustering coefficients for "SC" contact type. **D.** average diameters for "BB" contact type. **E.** average diameters for "ALL" contact type. **F.** average diameters for "SC" contact type.

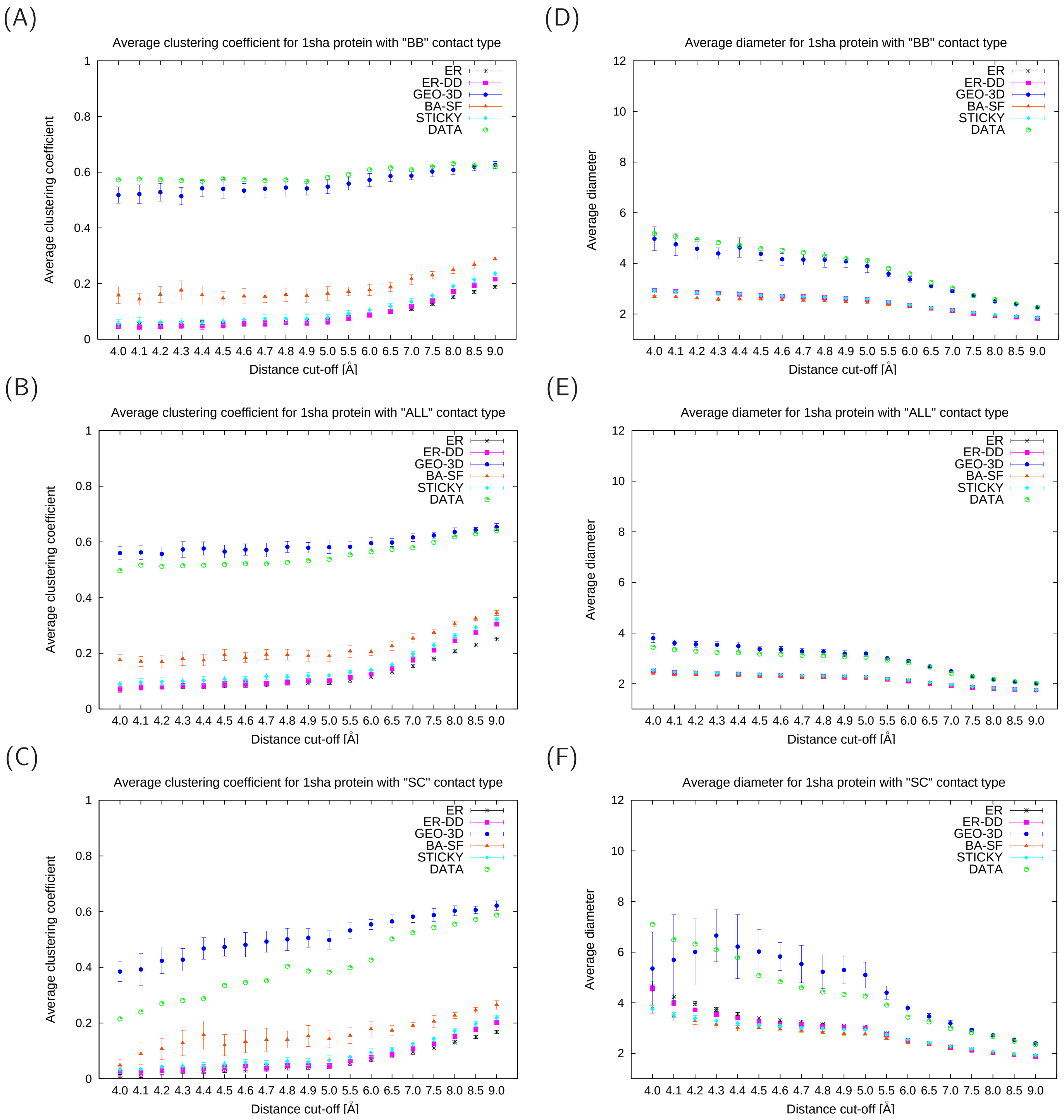

Figure S1.25 The agreements of average clustering coefficients and diameters of model networks (ER, ER-DD, GEO-3D, SF-BA, and STICKY) and RIGs corresponding to 1sha protein that are constructed for each of the three contact types (“BB”, “ALL”, and “SC”) and a series of distance cut-off values between 4.0 and 9.0 Angstroms: **A.** average clustering coefficients for “BB” contact type. **B.** average clustering coefficients for “ALL” contact type. **C.** average clustering coefficients for “SC” contact type. **D.** average diameters for “BB” contact type. **E.** average diameters for “ALL” contact type. **F.** average diameters for “SC” contact type.

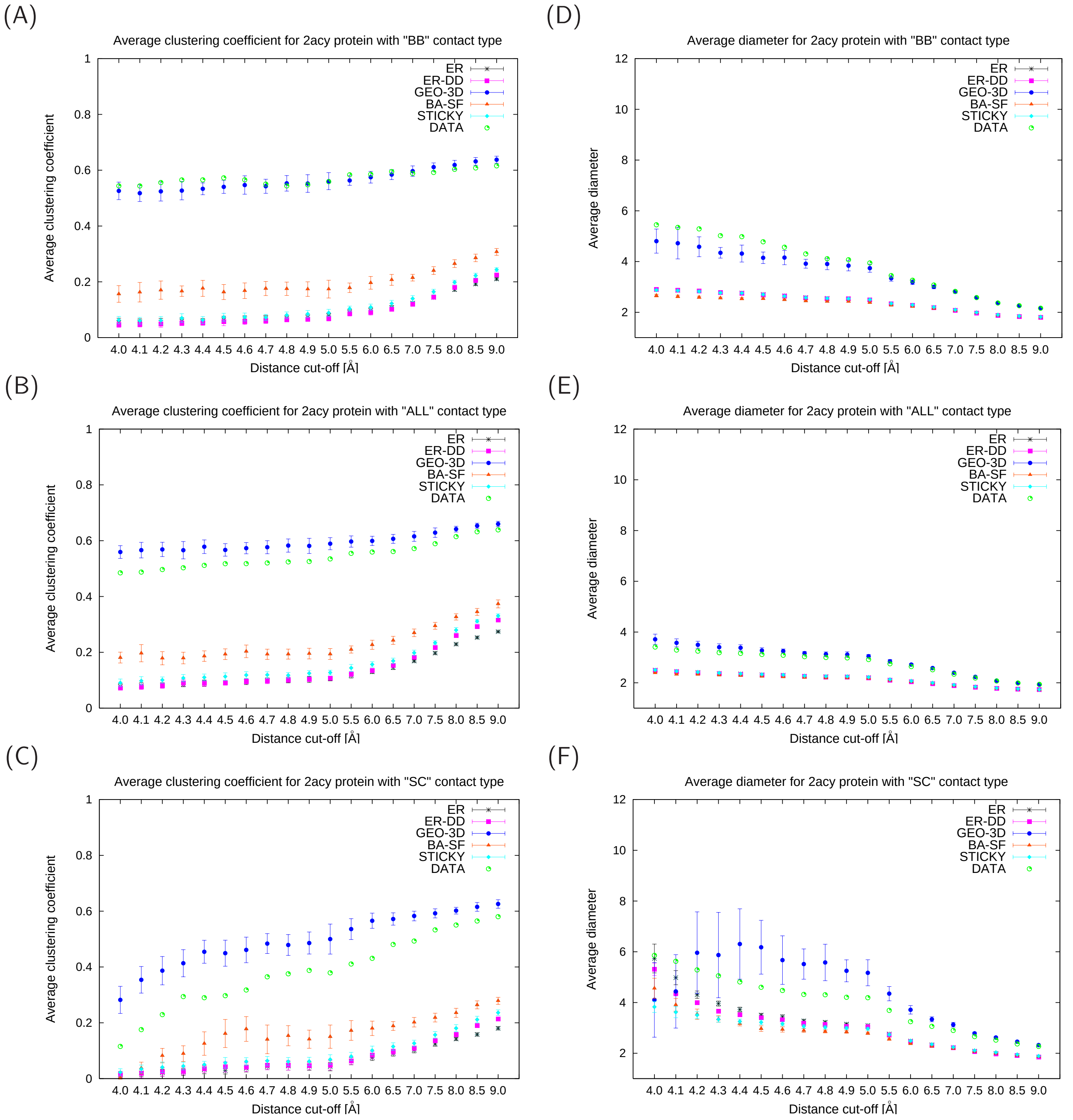

Figure S1.26 The agreements of average clustering coefficients and diameters of model networks (ER, ER-DD, GEO-3D, SF-BA, and STICKY) and RIGs corresponding to 2acy protein that are constructed for each of the three contact types ("BB", "ALL", and "SC") and a series of distance cut-off values between 4.0 and 9.0 Angstroms: **A.** average clustering coefficients for "BB" contact type. **B.** average clustering coefficients for "ALL" contact type. **C.** average clustering coefficients for "SC" contact type. **D.** average diameters for "BB" contact type. **E.** average diameters for "ALL" contact type. **F.** average diameters for "SC" contact type.

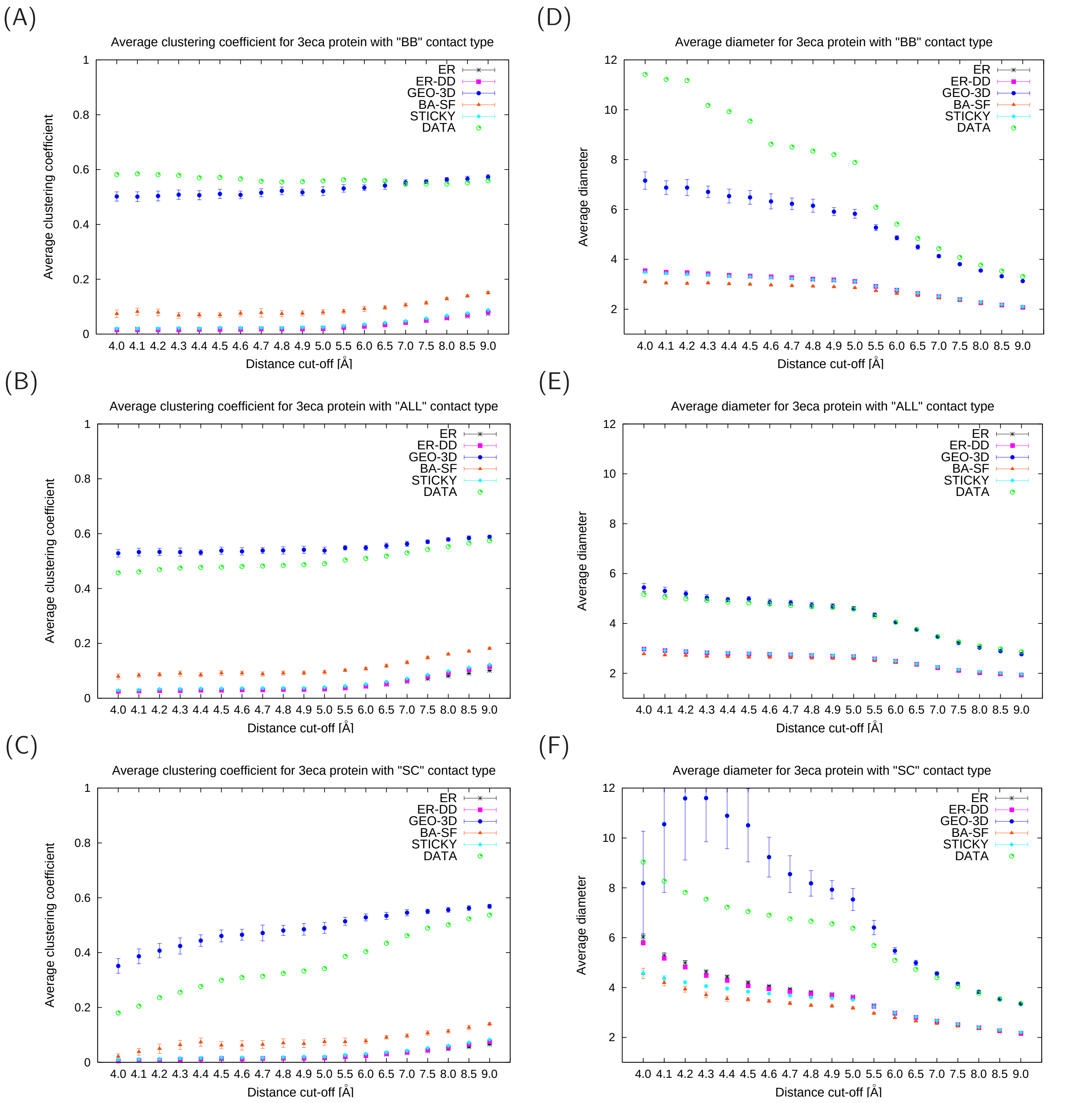

Figure S1.27 The agreements of average clustering coefficients and diameters of model networks (ER, ER-DD, GEO-3D, SF-BA, and STICKY) and RIGs corresponding to 3eca protein that are constructed for each of the three contact types ("BB", "ALL", and "SC") and a series of distance cut-off values between 4.0 and 9.0 Angstroms: **A.** average clustering coefficients for "BB" contact type. **B.** average clustering coefficients for "ALL" contact type. **C.** average clustering coefficients for "SC" contact type. **D.** average diameters for "BB" contact type. **E.** average diameters for "ALL" contact type. **F.** average diameters for "SC" contact type.

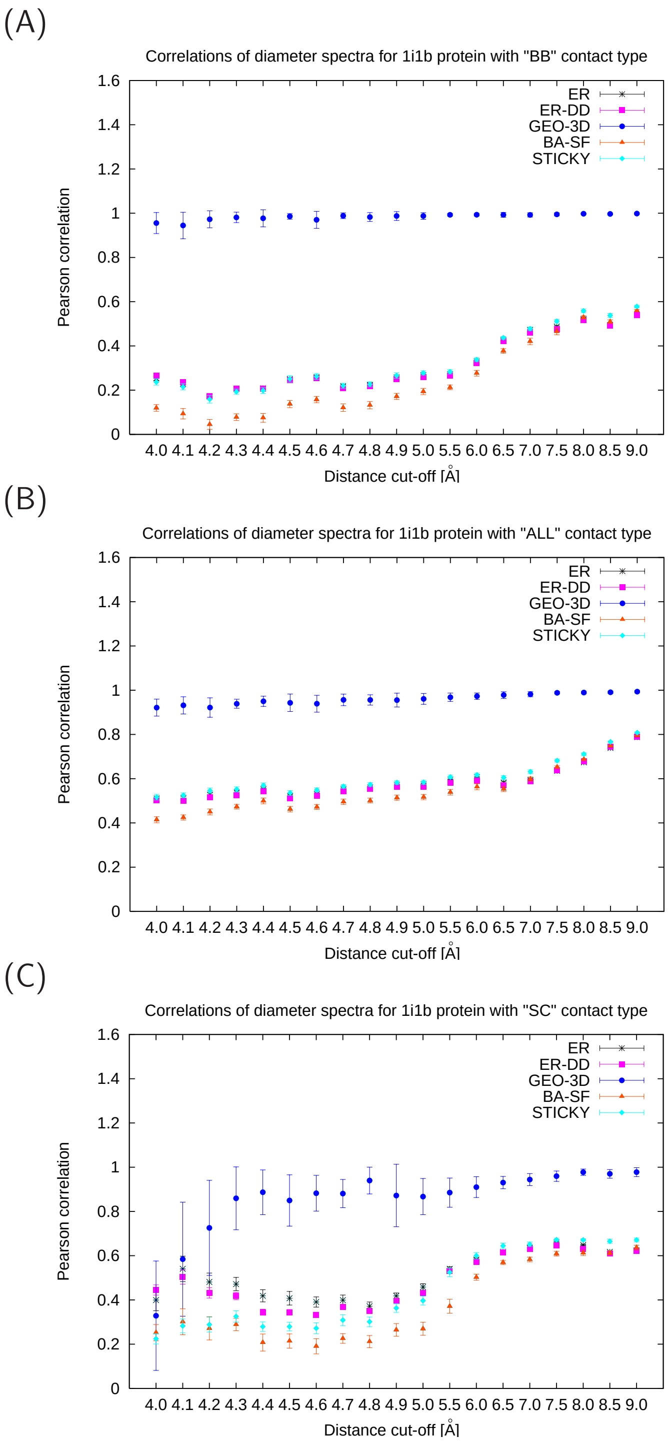

Figure S1.28 The Pearson correlation coefficients of the shortest path lengths spectra of model networks (ER, ER-DD, GEO-3D, SF-BA, and STICKY) and RIGs corresponding to 1i1b protein that are constructed for each of the three contact types ("BB", "ALL", and "SC") and a series of distance cut-off values between 4.0 and 9.0 Angstroms: **A.** spectra of shortest path lengths for "BB" contact type. **B.** spectra of shortest path lengths for "ALL" contact type. **C.** spectra of shortest path lengths for "SC" contact type.

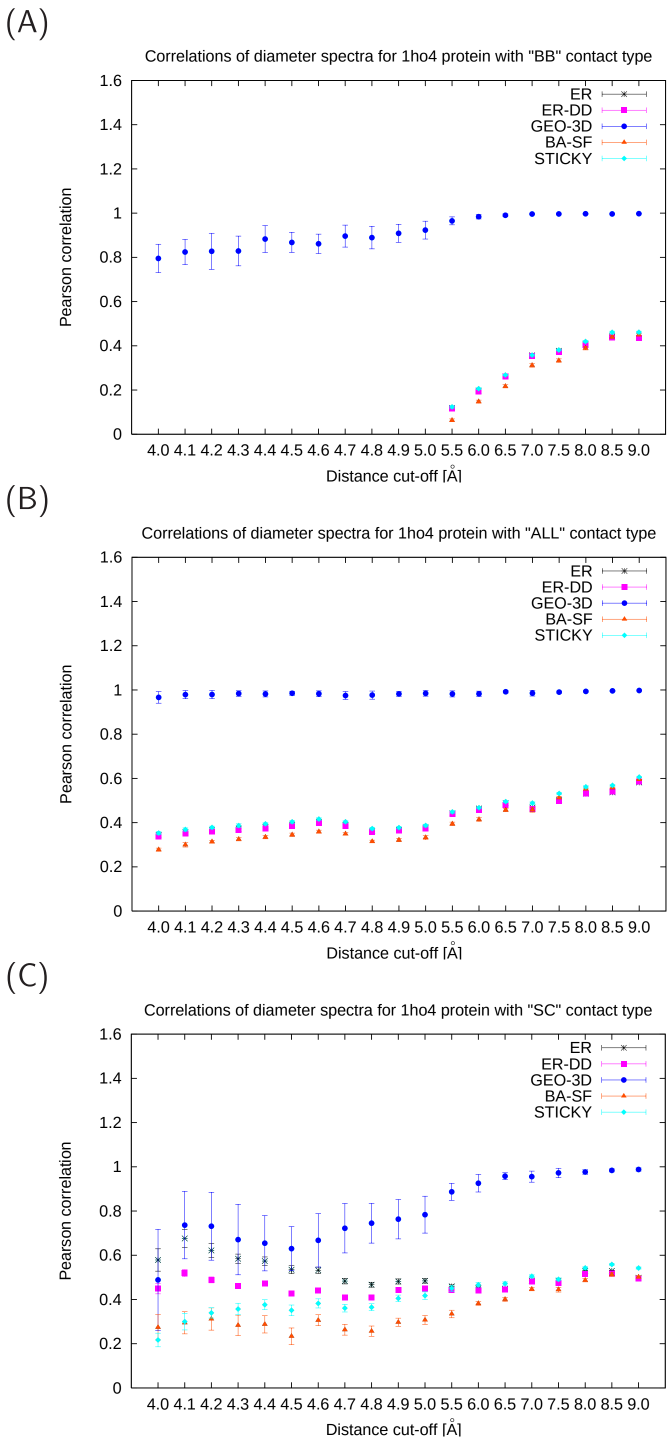

Figure S1.29 The Pearson correlation coefficients of the shortest path lengths spectra of model networks (ER, ER-DD, GEO-3D, SF-BA, and STICKY) and RIGs corresponding to 1ho4 protein that are constructed for each of the three contact types (“BB”, “ALL”, and “SC”) and a series of distance cut-off values between 4.0 and 9.0 Angstroms: **A.** spectra of shortest path lengths for “BB” contact type. **B.** spectra of shortest path lengths for “ALL” contact type. **C.** spectra of shortest path lengths for “SC” contact type.

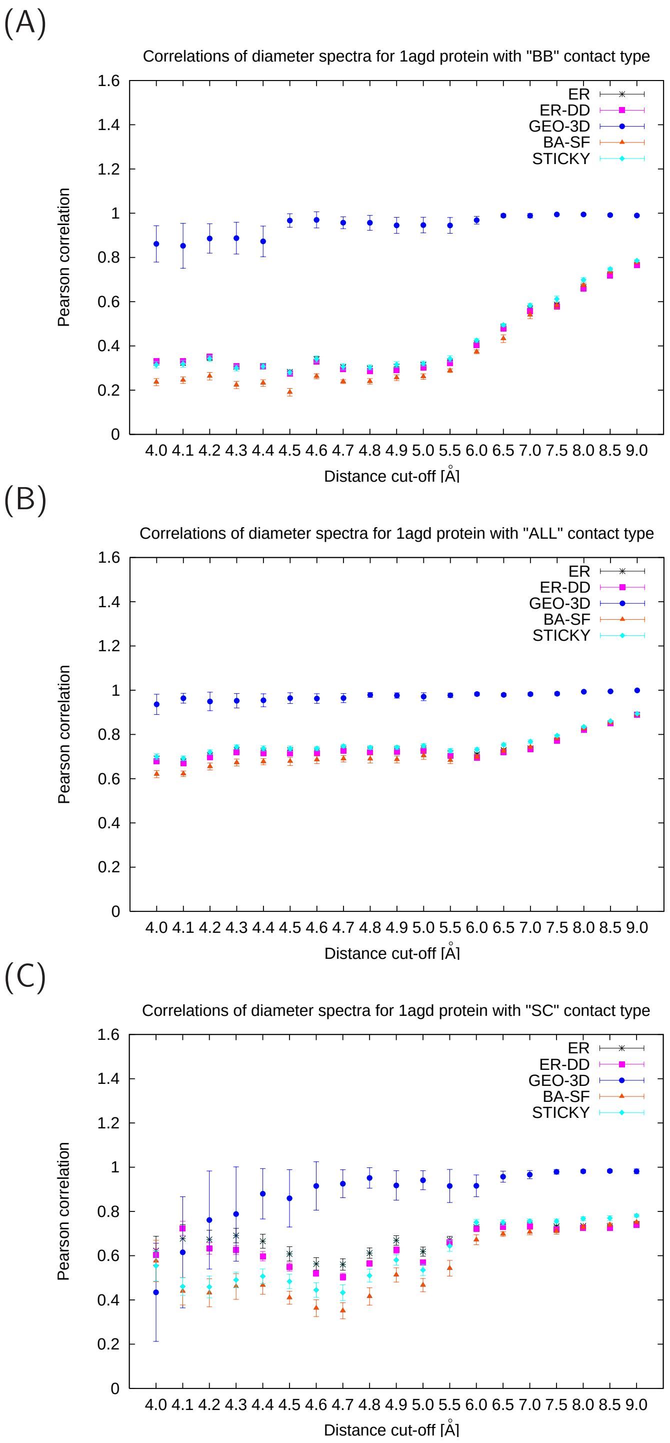

Figure S1.30 The Pearson correlation coefficients of the shortest path lengths spectra of model networks (ER, ER-DD, GEO-3D, SF-BA, and STICKY) and RIGs corresponding to 1agd protein that are constructed for each of the three contact types ("BB", "ALL", and "SC") and a series of distance cut-off values between 4.0 and 9.0 Angstroms: **A.** spectra of shortest path lengths for "BB" contact type. **B.** spectra of shortest path lengths for "ALL" contact type. **C.** spectra of shortest path lengths for "SC" contact type.

(A)

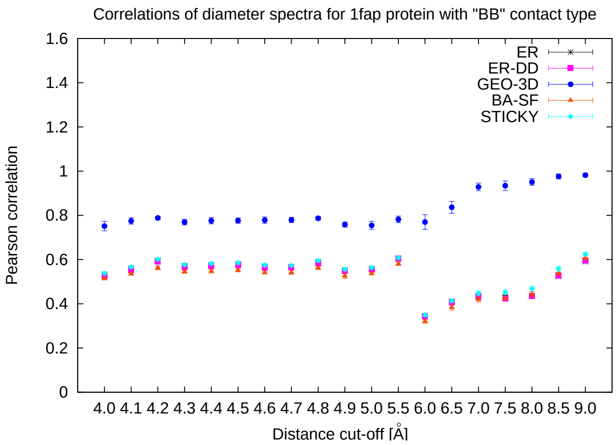

(B)

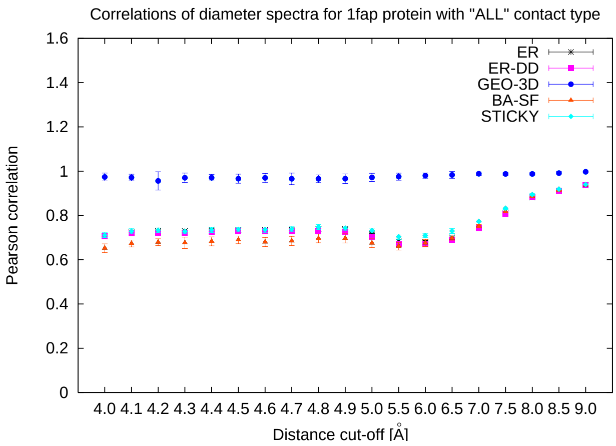

(C)

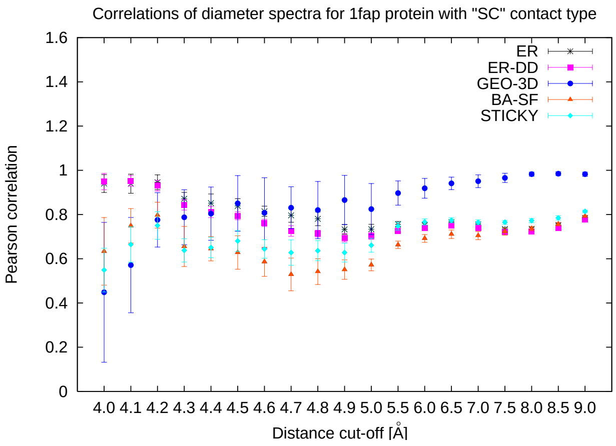

Figure S1.31 The Pearson correlation coefficients of the shortest path lengths spectra of model networks (ER, ER-DD, GEO-3D, SF-BA, and STICKY) and RIGs corresponding to 1fap protein that are constructed for each of the three contact types ("BB", "ALL", and "SC") and a series of distance cut-off values between 4.0 and 9.0 Angstroms: **A.** spectra of shortest path lengths for "BB" contact type. **B.** spectra of shortest path lengths for "ALL" contact type. **C.** spectra of shortest path lengths for "SC" contact type.

(A)

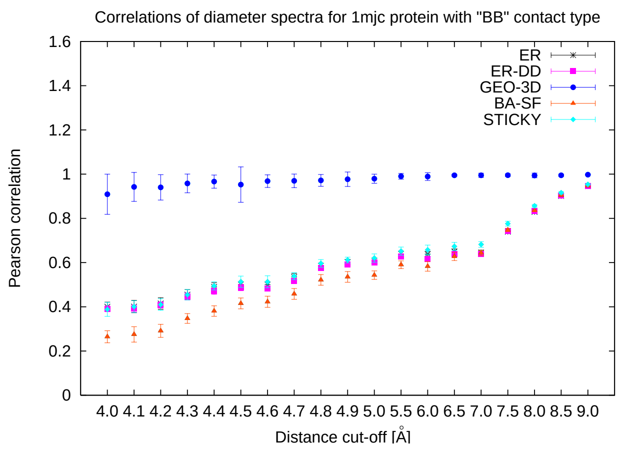

(B)

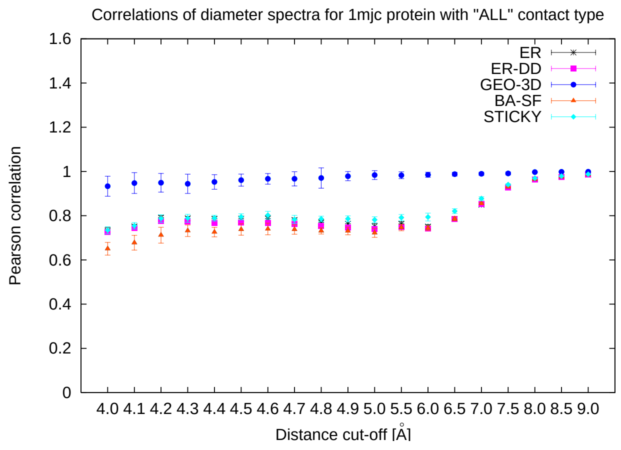

(C)

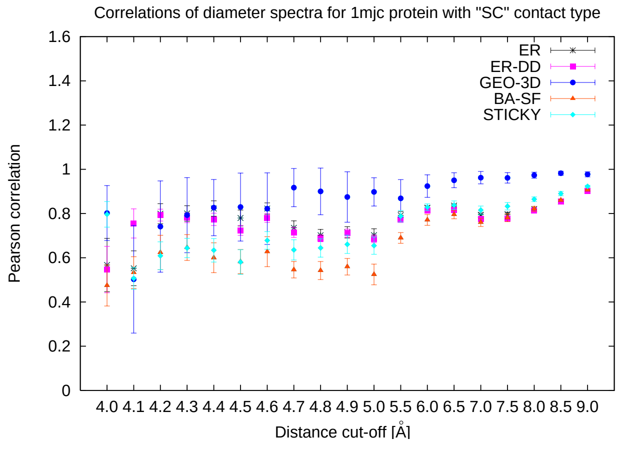

Figure S1.32 The Pearson correlation coefficients of the shortest path lengths spectra of model networks (ER, ER-DD, GEO-3D, SF-BA, and STICKY) and RIGs corresponding to 1mjc protein that are constructed for each of the three contact types ("BB", "ALL", and "SC") and a series of distance cut-off values between 4.0 and 9.0 Angstroms: **A.** spectra of shortest path lengths for "BB" contact type. **B.** spectra of shortest path lengths for "ALL" contact type. **C.** spectra of shortest path lengths for "SC" contact type.

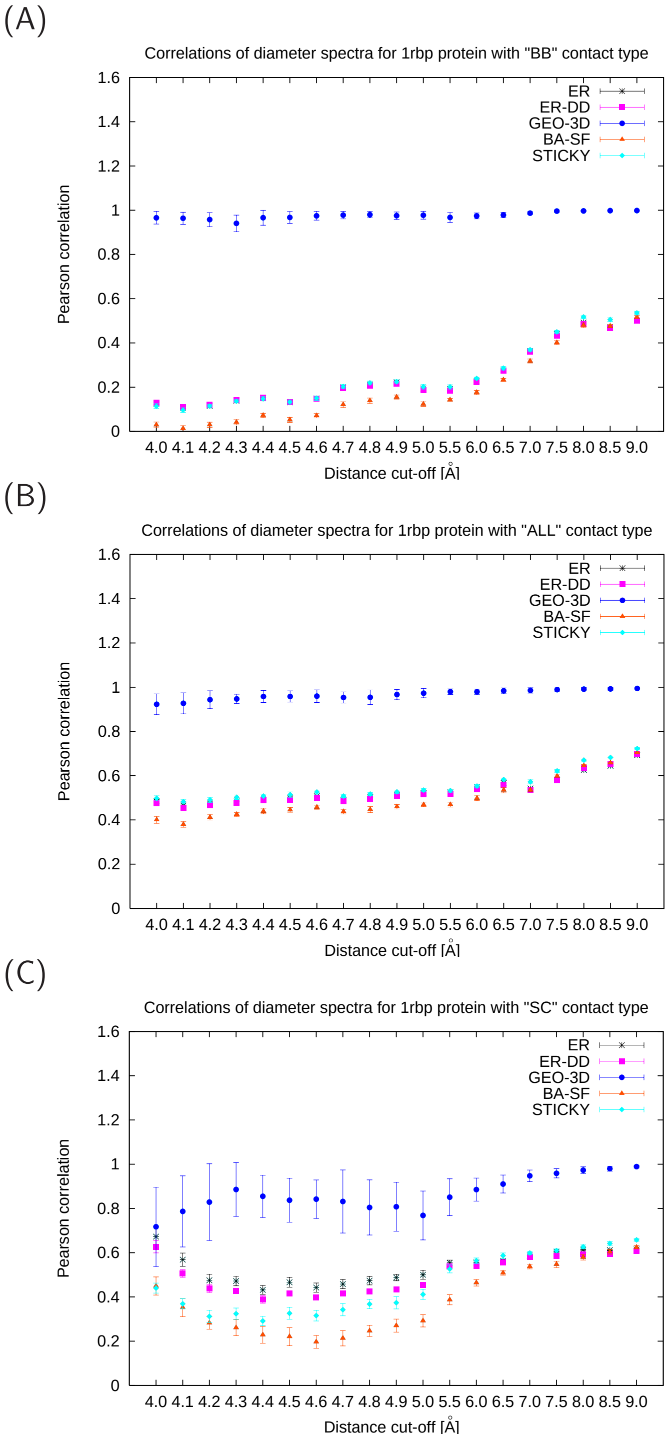

Figure S1.33 The Pearson correlation coefficients of the shortest path lengths spectra of model networks (ER, ER-DD, GEO-3D, SF-BA, and STICKY) and RIGs corresponding to 1rbp protein that are constructed for each of the three contact types (“BB”, “ALL”, and “SC”) and a series of distance cut-off values between 4.0 and 9.0 Angstroms: **A.** spectra of shortest path lengths for “BB” contact type. **B.** spectra of shortest path lengths for “ALL” contact type. **C.** spectra of shortest path lengths for “SC” contact type.

(A)

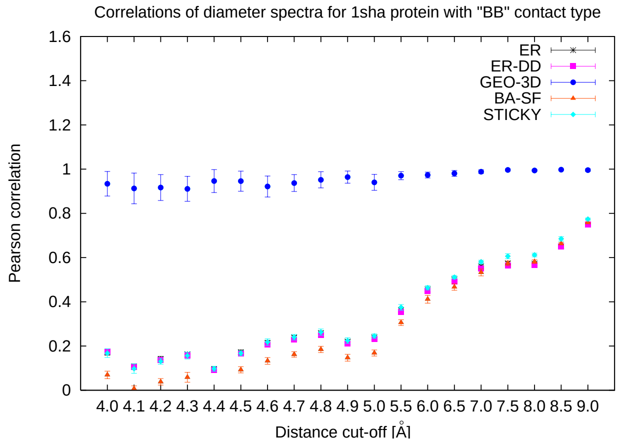

(B)

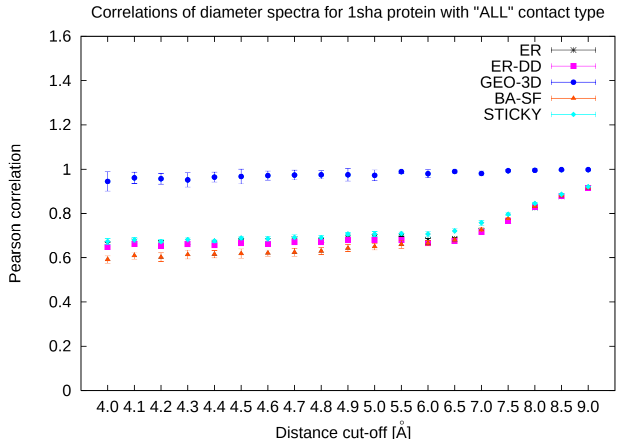

(C)

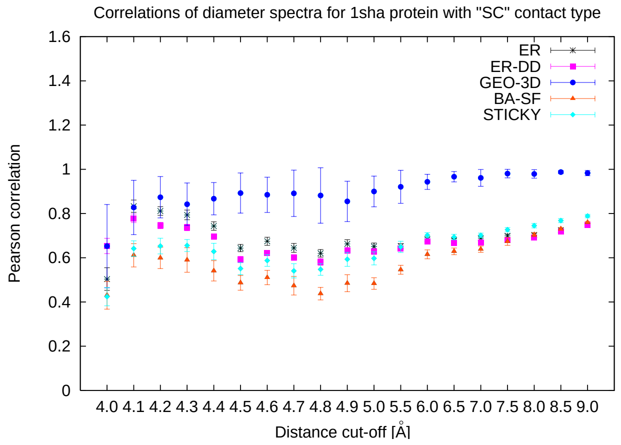

Figure S1.34 The Pearson correlation coefficients of the shortest path lengths spectra of model networks (ER, ER-DD, GEO-3D, SF-BA, and STICKY) and RIGs corresponding to 1sha protein that are constructed for each of the three contact types ("BB", "ALL", and "SC") and a series of distance cut-off values between 4.0 and 9.0 Angstroms: **A.** spectra of shortest path lengths for "BB" contact type. **B.** spectra of shortest path lengths for "ALL" contact type. **C.** spectra of shortest path lengths for "SC" contact type.

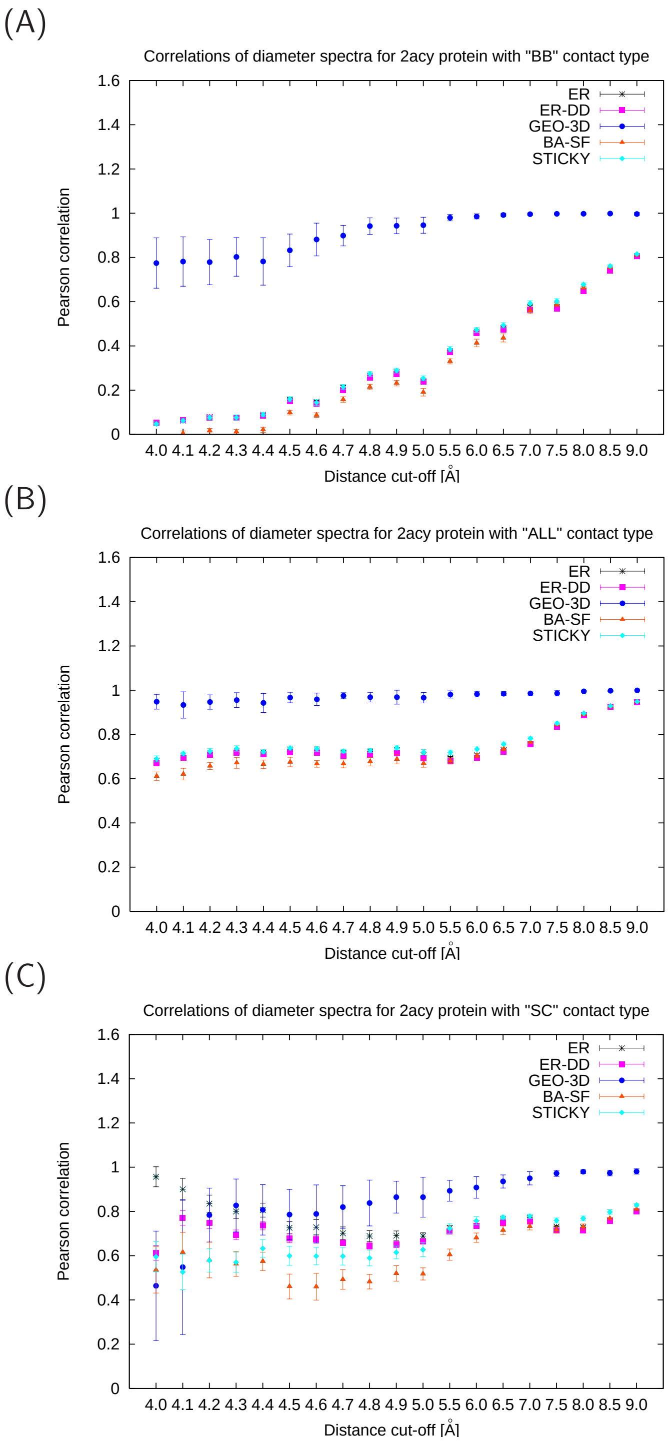

Figure S1.35 The Pearson correlation coefficients of the shortest path lengths spectra of model networks (ER, ER-DD, GEO-3D, SF-BA, and STICKY) and RIGs corresponding to 2acy protein that are constructed for each of the three contact types ("BB", "ALL", and "SC") and a series of distance cut-off values between 4.0 and 9.0 Angstroms: **A.** spectra of shortest path lengths for "BB" contact type. **B.** spectra of shortest path lengths for "ALL" contact type. **C.** spectra of shortest path lengths for "SC" contact type.

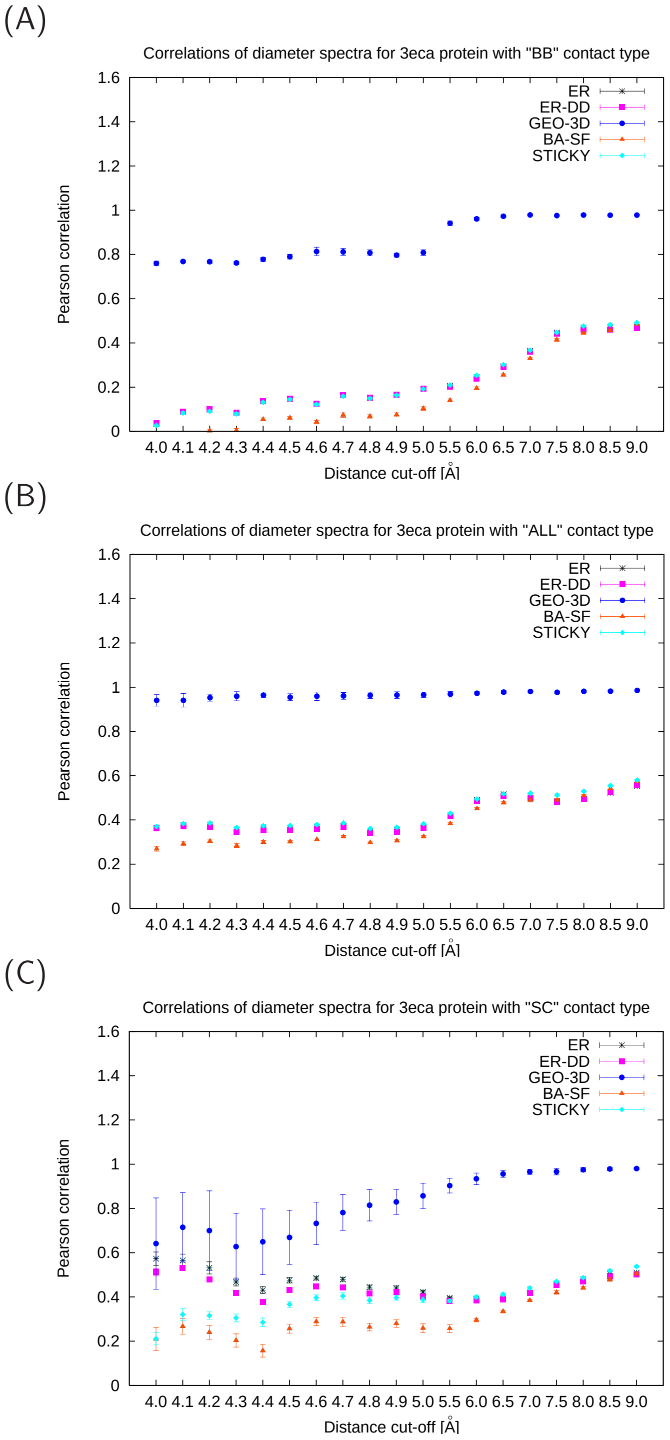

Figure S1.36 The Pearson correlation coefficients of the shortest path lengths spectra of model networks (ER, ER-DD, GEO-3D, SF-BA, and STICKY) and RIGs corresponding to 3eca protein that are constructed for each of the three contact types (“BB”, “ALL”, and “SC”) and a series of distance cut-off values between 4.0 and 9.0 Angstroms: **A.** spectra of shortest path lengths for “BB” contact type. **B.** spectra of shortest path lengths for “ALL” contact type. **C.** spectra of shortest path lengths for “SC” contact type.

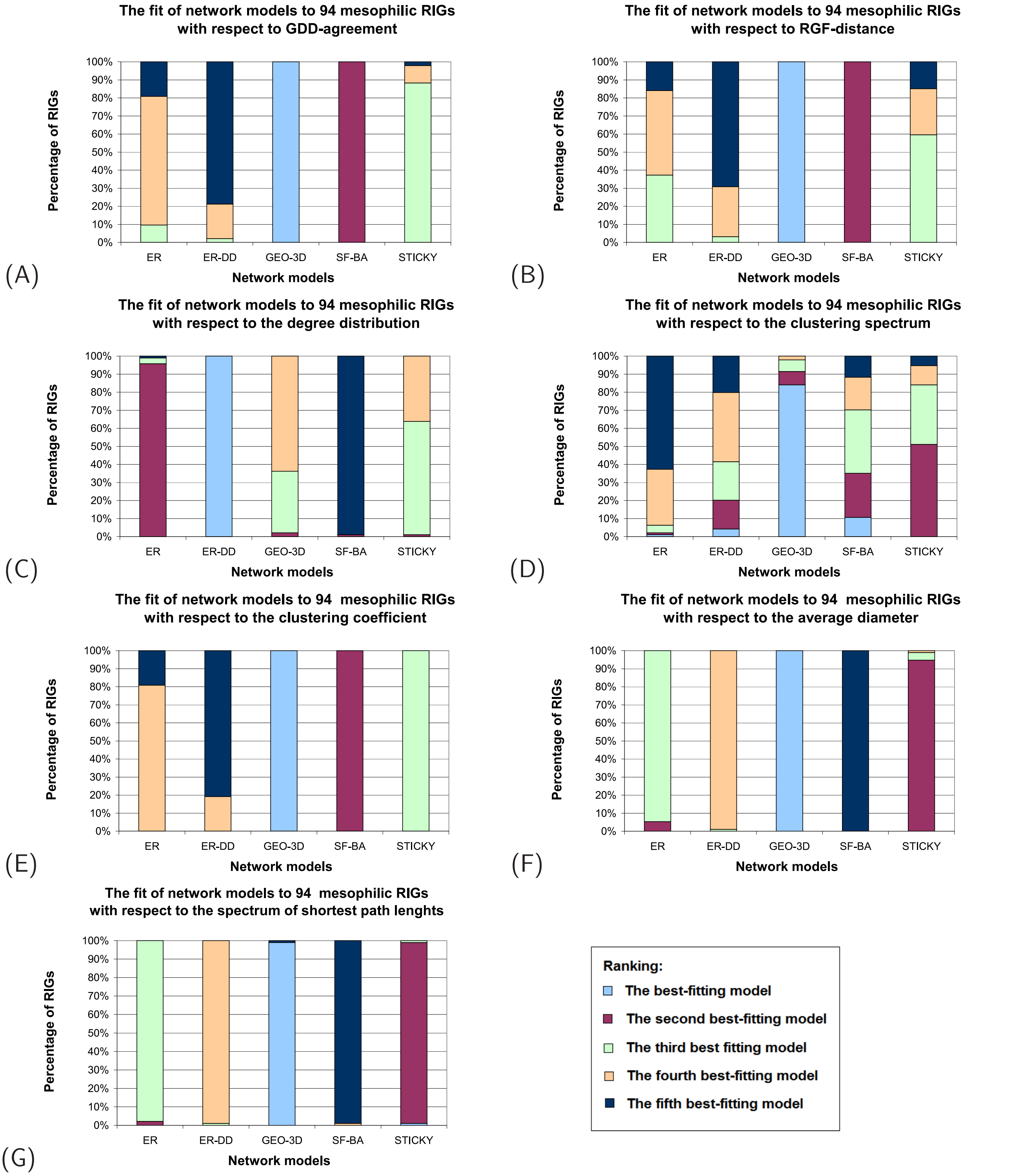

Figure S1.37 The ranking of five network models (ER, ER-DD, GEO-3D, SF-BA, and STICKY) for 94 RIGs corresponding to the 94 mesophilic proteins, constructed with "ALL" contact type and distance cut-off of 4.5 Å . The ranking is based on: (A) GDD-agreements between RIGs and model networks, (B) RGF-distances between RIGs and model networks, (C) agreements between degree distributions of RIGs and model networks, (D) agreements between clustering spectra of RIGs and model networks, (E) agreements between clustering coefficients of RIGs and model networks, (F) agreements between average diameters of RIGs and model networks, and (G) agreements between spectra of shortest path lengths of RIGs and model networks.

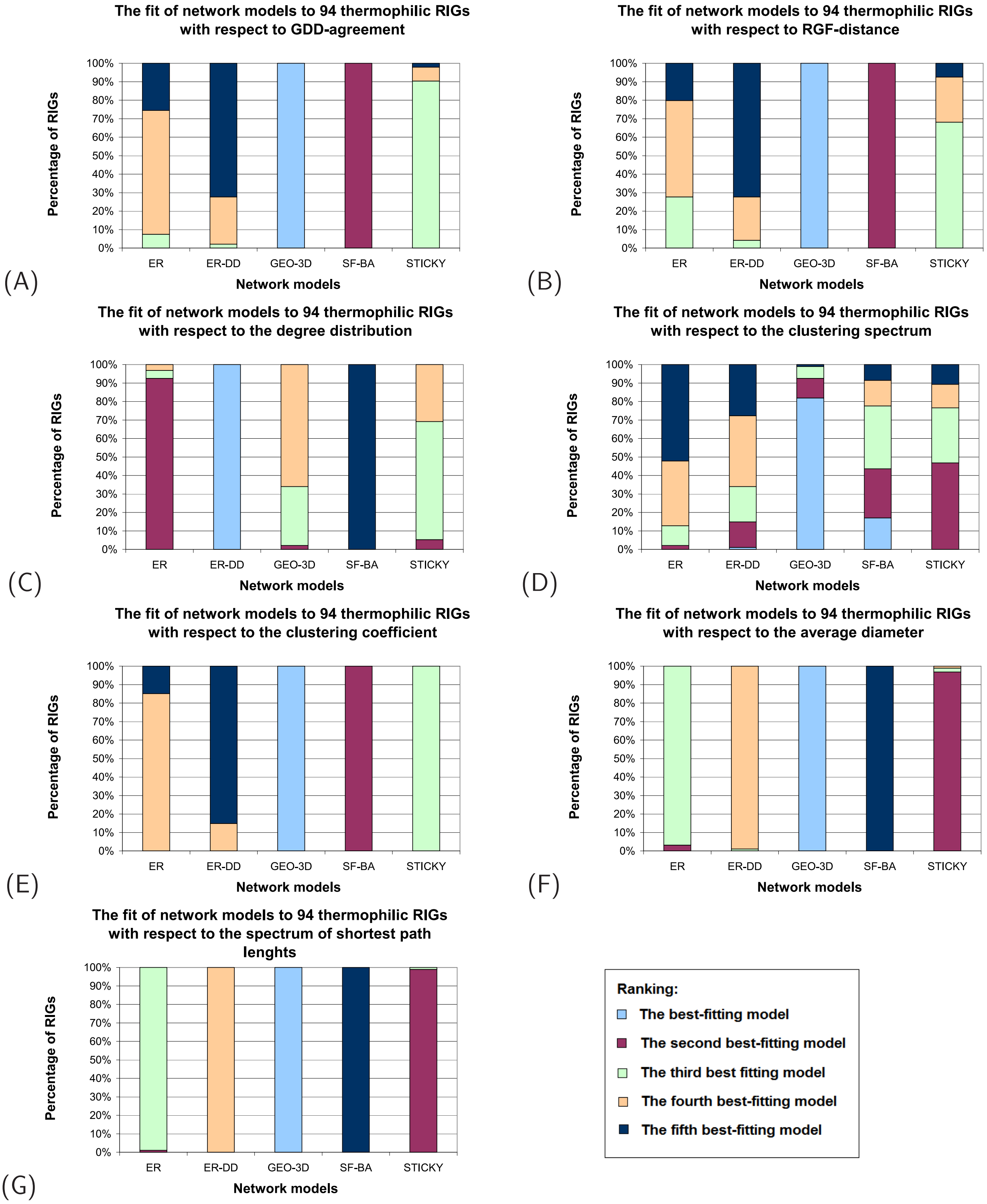

Figure S1.38 The ranking of five network models (ER, ER-DD, GEO-3D, SF-BA, and STICKY) for 94 RIGs corresponding to the 94 thermophilic proteins, constructed with "ALL" contact type and distance cut-off of 4.5 Å. The ranking is based on: (A) GDD-agreements between RIGs and model networks, (B) RGF-distances between RIGs and model networks, (C) agreements between degree distributions of RIGs and model networks, (D) agreements between clustering spectra of RIGs and model networks, (E) agreements between clustering coefficients of RIGs and model networks, (F) agreements between average diameters of RIGs and model networks, and (G) agreements between spectra of shortest path lengths of RIGs and model networks.

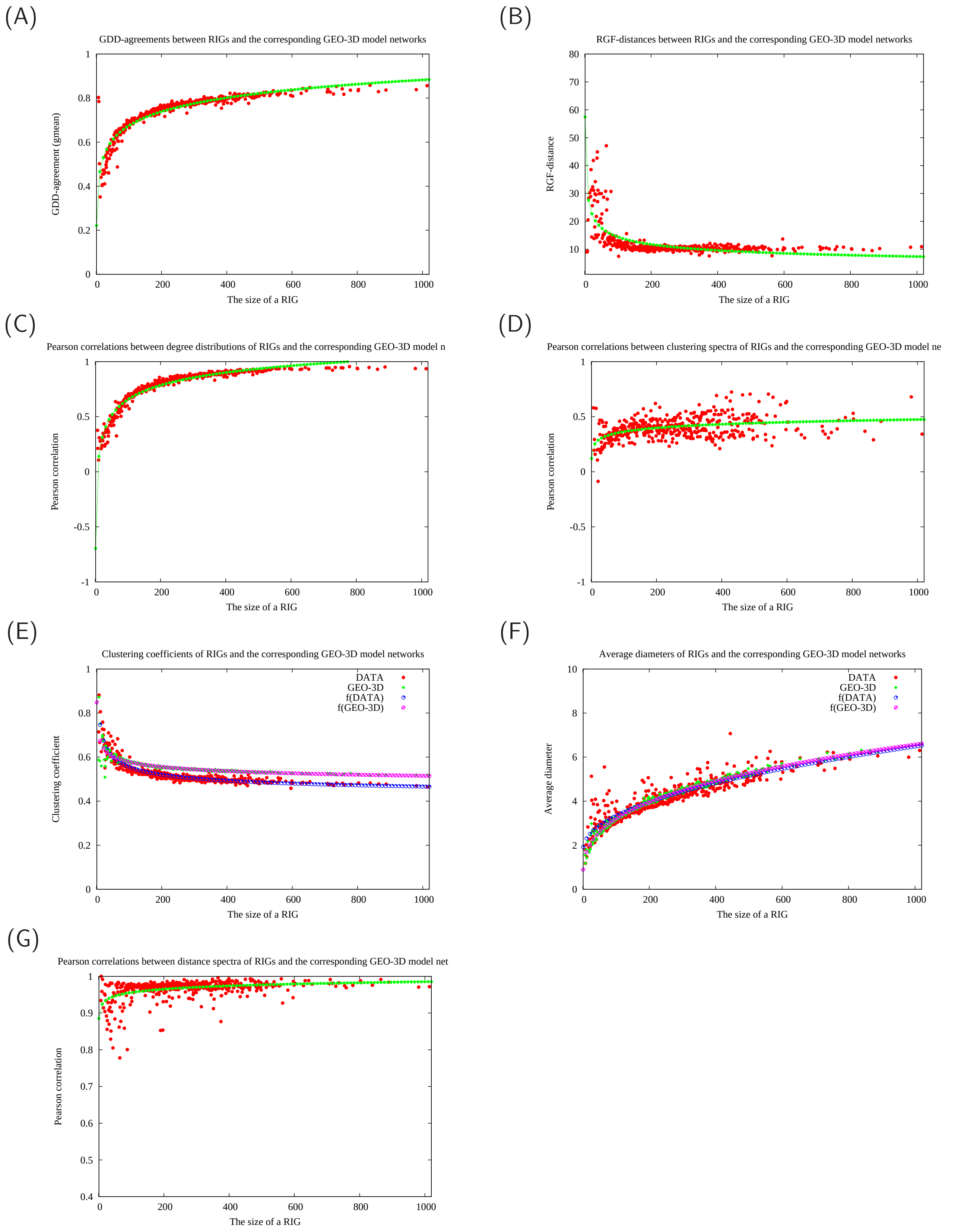

Figure S1.39 Network property values describing the fit of RIGs to GEO-3D graphs and the fitted power-law functions. 1,272 RIGs from Data Set 2 were analyzed with respect to: (A) GDD-agreements, (B) RGF-distances, (C) agreements between degree distributions, (D) agreements between clustering spectra, (E) clustering coefficients of RIGs and the corresponding GEO-3D model networks, (F) average diameters of RIGs and the corresponding GEO-3D model networks, and (G) agreements between spectra of shortest path lengths.

Data set	Property	a	b	c	R-Square
The entire data set (1,272 RIGs)	GDDA	-4.13299	-0.02524	4.35428	0.83710
	RGFD	56.89579	-0.30680	0.54893	0.42765
	DD	-2.56226	-0.16289	1.86617	0.93543
	DS	-0.32687	-0.05289	1.21211	0.16531
	CS	-1.99221	-0.02822	2.11350	0.17694
	CC DATA	0.84706	-0.36712	0.39925	0.86259
	CC GEO	0.43196	-0.21384	0.41652	0.76697
	AD DATA	0.18323	0.47092	1.73757	0.81732
	AD GEO	0.63993	0.33136	0.25469	0.98958
Class A	GDDA	-2.37950	-0.51151	0.90775	0.93963
	RGFD	3723.38465	-1.60669	10.15264	0.57514
	DD	-3.85813	-0.42567	1.20449	0.95217
	DS	0.00024	0.79359	0.94487	0.01139
	CS	-1.19414	-0.51363	0.48532	0.11862
	CC DATA	1.45262	-0.54662	0.44763	0.85187
	CC GEO	0.52558	-0.32987	0.46379	0.94726
	AD DATA	0.58752	0.32954	0.64482	0.65354
	AD GEO	0.89578	0.29300	-0.26590	0.98199
Class B	GDDA	-1.28659	-0.14318	1.35182	0.93826
	RGFD	103.68002	-0.64369	7.25261	0.51435
	DD	-2.55698	-0.15931	1.89345	0.92415
	DS	-0.27778	-0.73719	0.97441	0.09980
	CS	0.00000	2.30896	0.38174	0.04630
	CC DATA	0.57862	-0.15441	0.25507	0.82357
	CC GEO	-0.00087	0.71667	0.59825	0.68834
	AD DATA	0.02537	0.75145	2.42600	0.64101
	AD GEO	0.17434	0.49691	1.49143	0.96078
Class C	GDDA	-1.39943	-0.32743	0.99518	0.87498
	RGFD	1.371E+13	-6.46140	10.47601	0.21543
	DD	-7.26862	-0.62686	1.06885	0.91158
	DS	0.00941	-0.38220	0.97351	0.00032
	CS	0.00131	0.70347	0.33768	0.03483
	CC DATA	10.58399	-1.08828	0.47959	0.71221
	CC GEO	0.41276	-0.20508	0.41598	0.93191
	AD DATA	0.39449	0.38867	0.72762	0.84718
	AD GEO	0.49040	0.36385	0.56849	0.98161
Class D	GDDA	-1.37701	-0.31108	1.01774	0.92754
	RGFD	1115.03720	-1.37919	9.56031	0.25267
	DD	-4.93768	-0.51017	1.13807	0.92221
	DS	-6331.66396	-3.28965	0.96929	0.01223
	CS	0.00008	1.14429	0.36063	0.04239
	CC DATA	2.00873	-0.69827	0.46556	0.71681
	CC GEO	0.47162	-0.12621	0.31449	0.86066
	AD DATA	0.10463	0.55671	1.87974	0.63692
	AD GEO	0.55392	0.34938	0.43300	0.96130

Figure S1.40 The coefficients a, b, and c for the fitted power-law functions $a * x^{b} + c$ and R-Square values measuring the goodness of fit with respect to the following network properties: GDD-agreement (GDDA), RGF-distance (RGFD), agreements between degree distributions (DD), agreements between clustering spectra (CS), clustering coefficients of RIGs (CC DATA) and the corresponding GEO-3D model networks (CC GEO), average diameters of RIGs (AD DATA) and the corresponding GEO-3D model networks (AD GEO), and agreements between spectra of shortest path lengths (DS). The statistics are presented for the entire Data Set 2 of 1,272 RIGs, as well as for individual groups of proteins belonging to the four structural classes: A, B, C, and D.

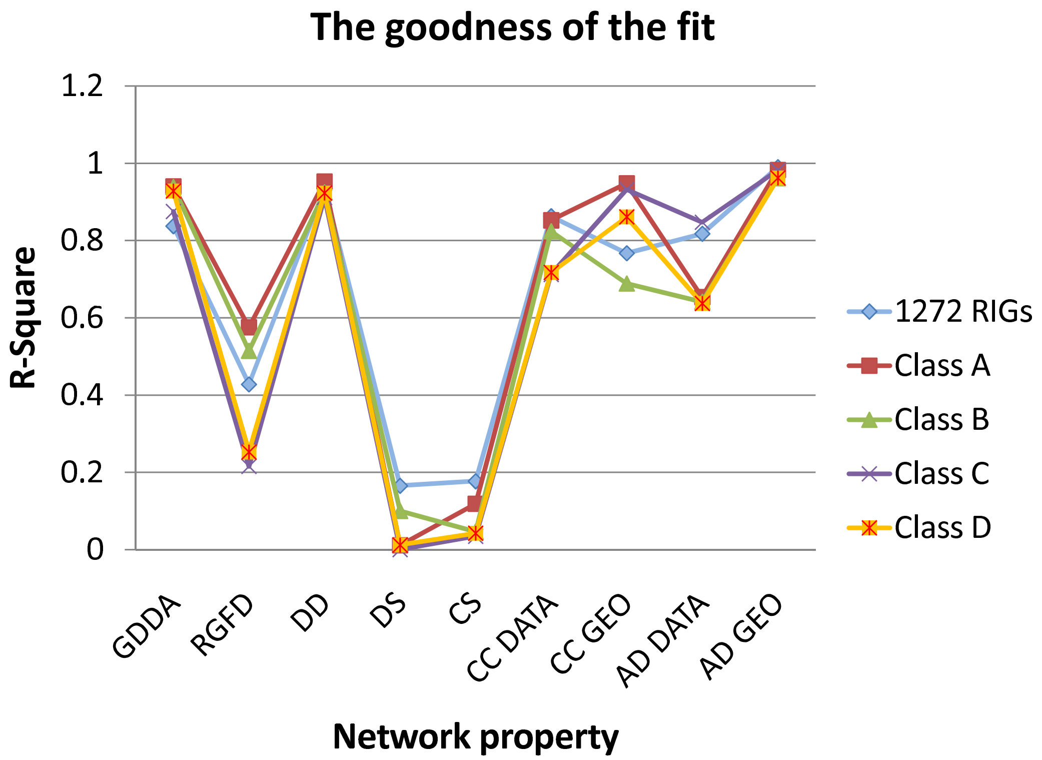

Figure S1.41 The goodness of fit of the fitted power-law functions, expressed in terms of R-squares, for the following network properties: GDD-agreement (GDDA), RGF-distance (RGFD), agreements between degree distributions (DD), agreements between clustering spectra (CS), clustering coefficients of RIGs (CC DATA) and the corresponding GEO-3D model networks (CC GEO), average diameters of RIGs (AD DATA) and the corresponding GEO-3D model networks (AD GEO), and agreements between spectra of shortest path lengths (DS). The entire Data Set 2 of 1,272 RIGs was analyzed, as well as for individual groups of proteins belonging to the four structural classes: A, B, C, and D.

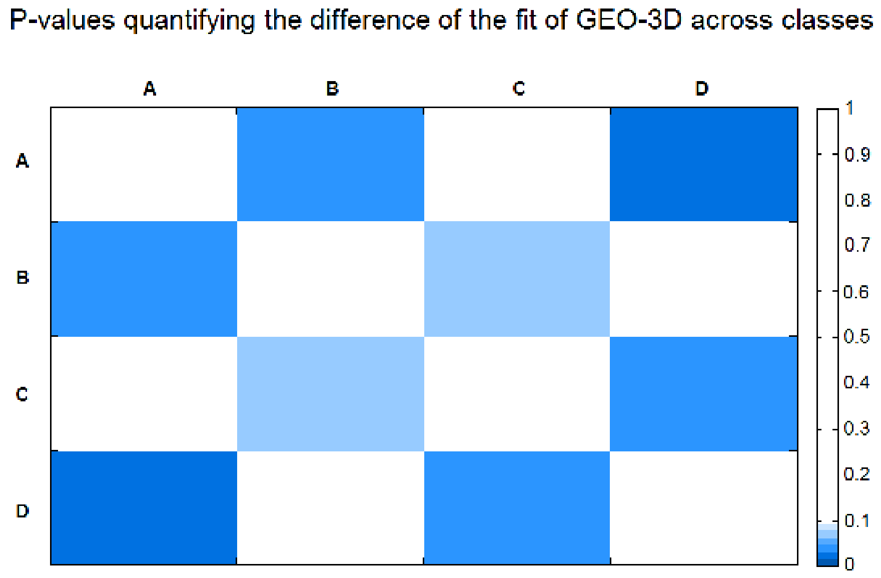

(A)

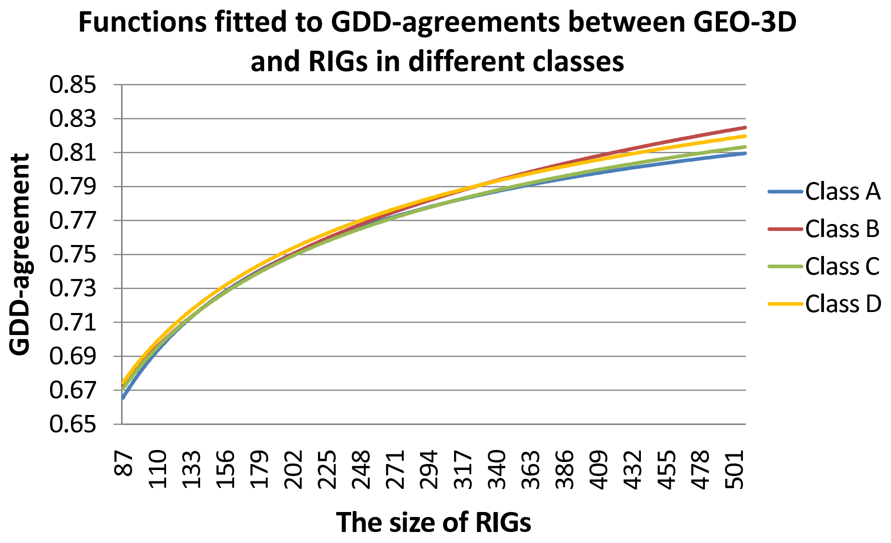

(B)

Figure S1.42 The fit of GEO-3D to RIGs in different classes. (A) *P*-values quantifying the difference in the fit of GEO-3D to proteins of a given size belonging to four different structural classes, A, B, C, and D, with respect to GDD-agreement. Low p-values indicate that the difference in the fit between two classes is significant. (B) The functions that are fitted to GDD-agreements between GEO-3D and RIGs in different classes, from which these p-values are computed.

(A)

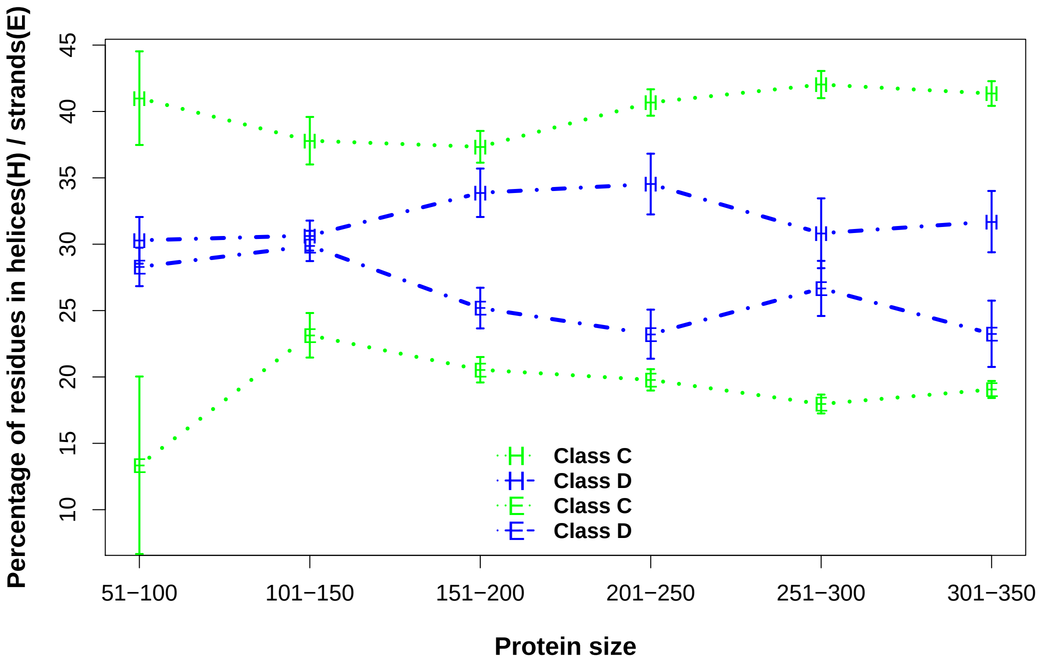

(B)

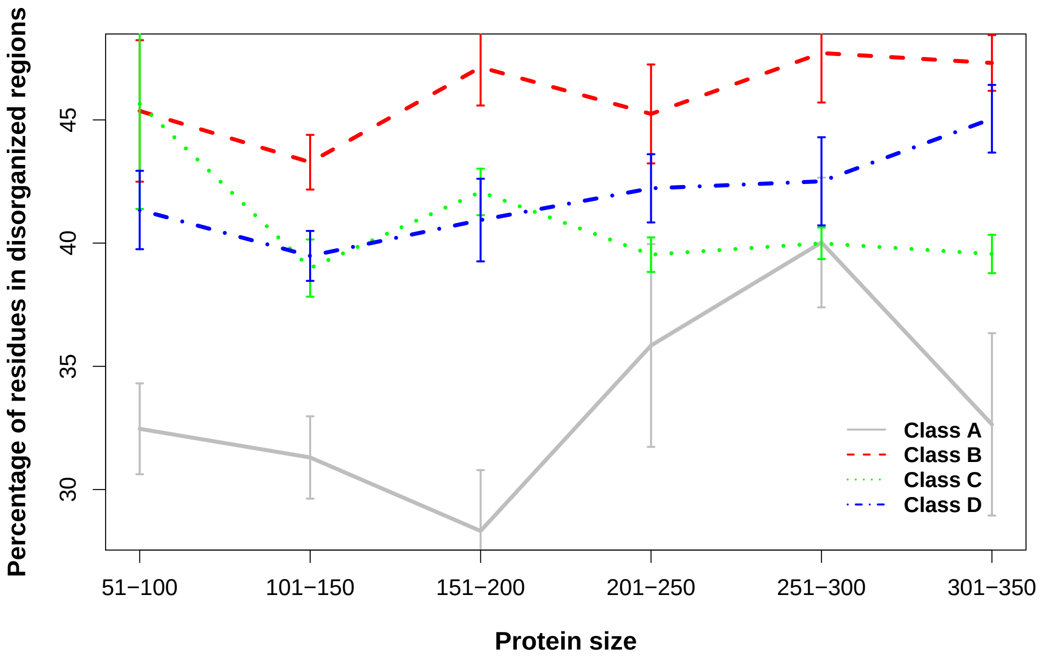

Figure S1.43 (A) Average percentage of residues in α-helices and β-strands for all proteins of class C and D in Data Set 2, with respect to each protein size range. (B) Average percentage of residues in disorganized regions for 744 proteins in Data Set 2 belonging to the four structural classes, with respect to each size range and each class. The standard error of the mean is plotted.

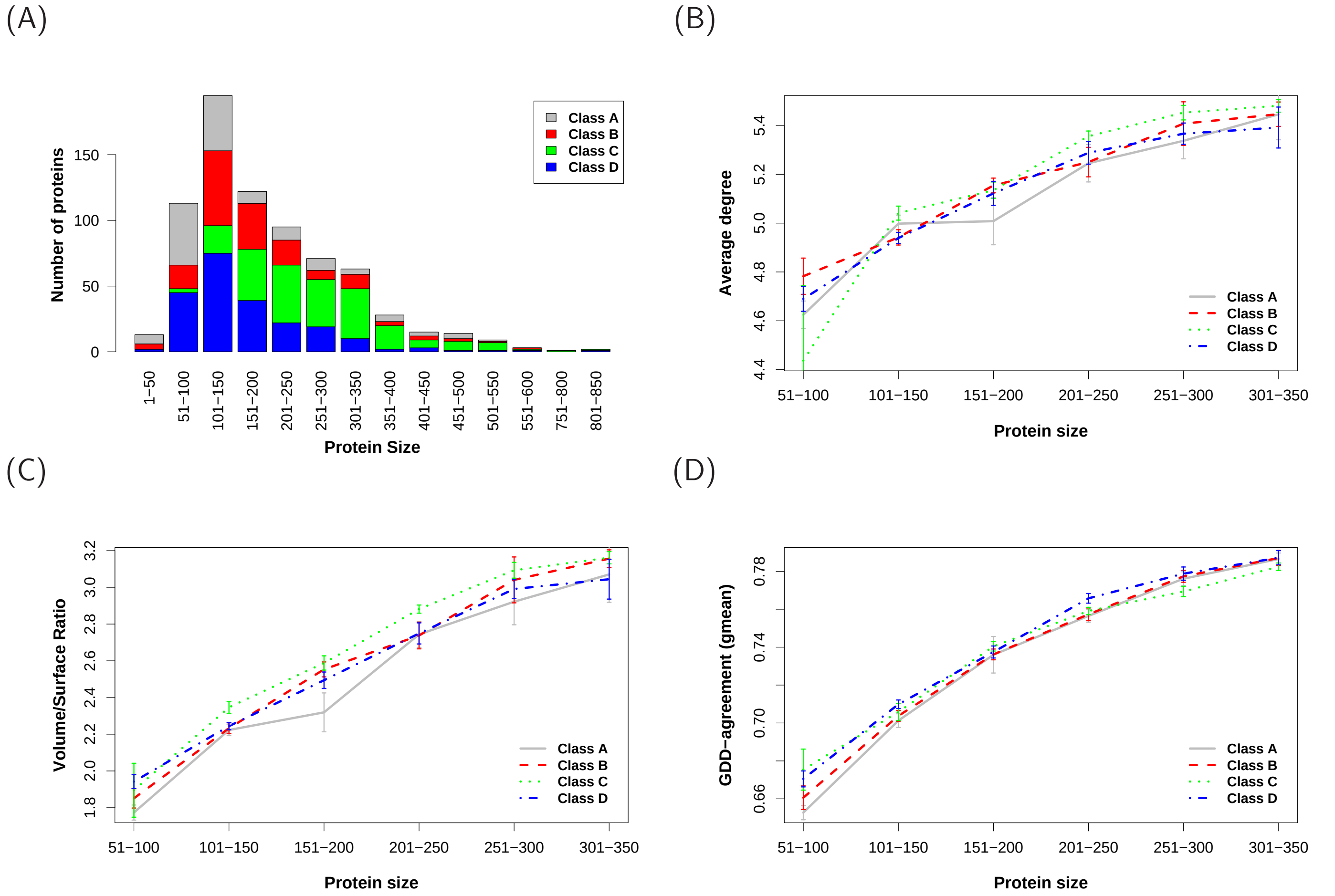

Figure S1.44 (A) Distribution of protein size for 744 proteins from Data Set 2 that belong to one of the four structural classes. (B) Average degree, (C) average volume-to-surface ratio, and (D) average GDD-agreement between GEO-3D graphs and the corresponding RIGs, with respect to each size range and each of the four structural classes. The standard error of the mean is plotted.

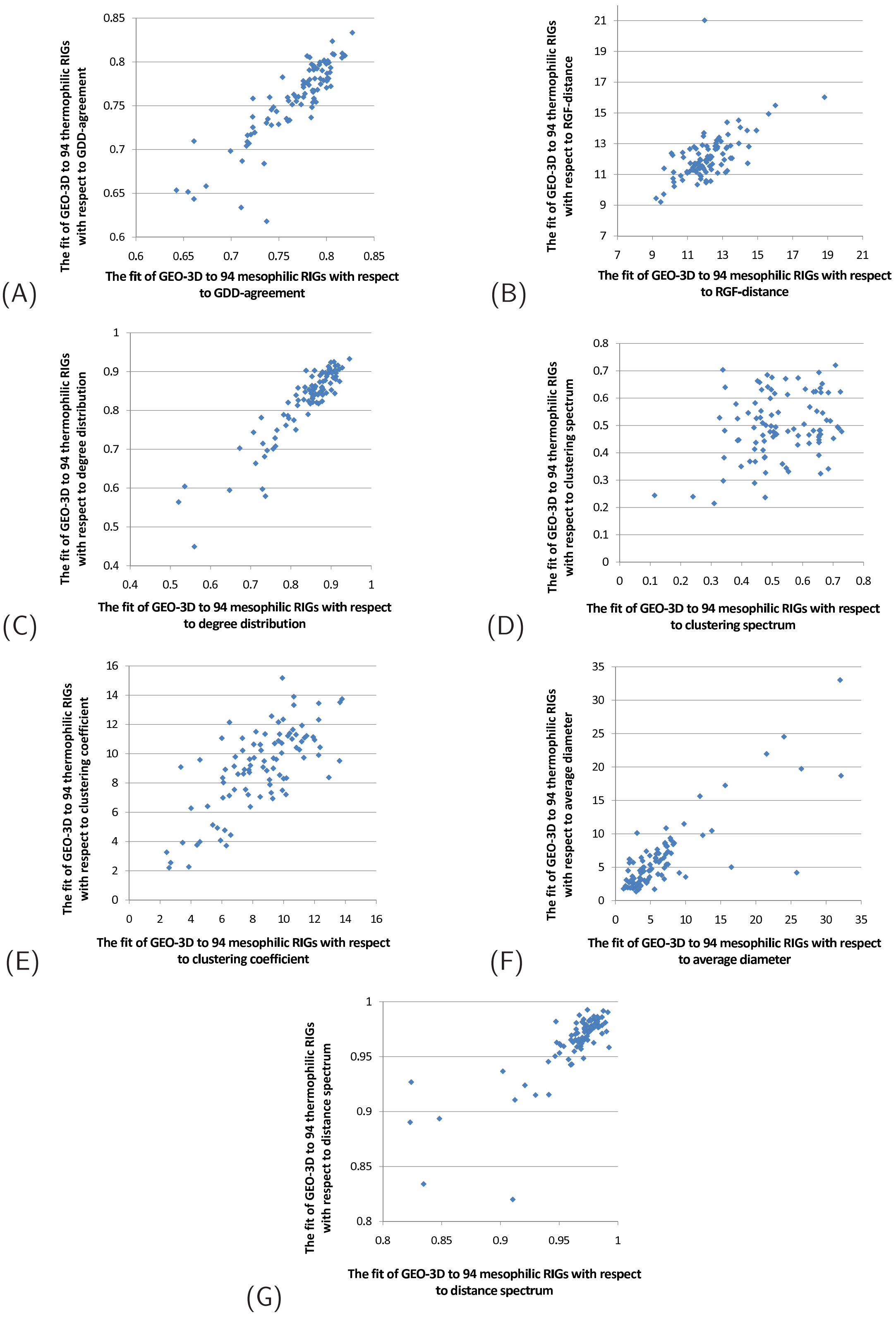

Figure S1.45 The fit of GEO-3D to the 94 thermophilic proteins versus its fit to their mesophilic homologs, with respect to: (A) GDD-agreements (GDDA) between RIGs and GEO-3D model networks, (B) RGF-distances (RGFD) between RIGs and GEO-3D model networks, (C) agreements between degree distributions (DD) of RIGs and GEO-3D model networks, (D) agreements between clustering spectra (CS) of RIGs and GEO-3D model networks, (E) agreements between clustering coefficients (CC) of RIGs and GEO-3D model networks, (F) agreements between average diameters (AD) of RIGs and GEO-3D model networks, and (G) agreements between spectra of shortest path lengths (DS) of RIGs and GEO-3D model networks.

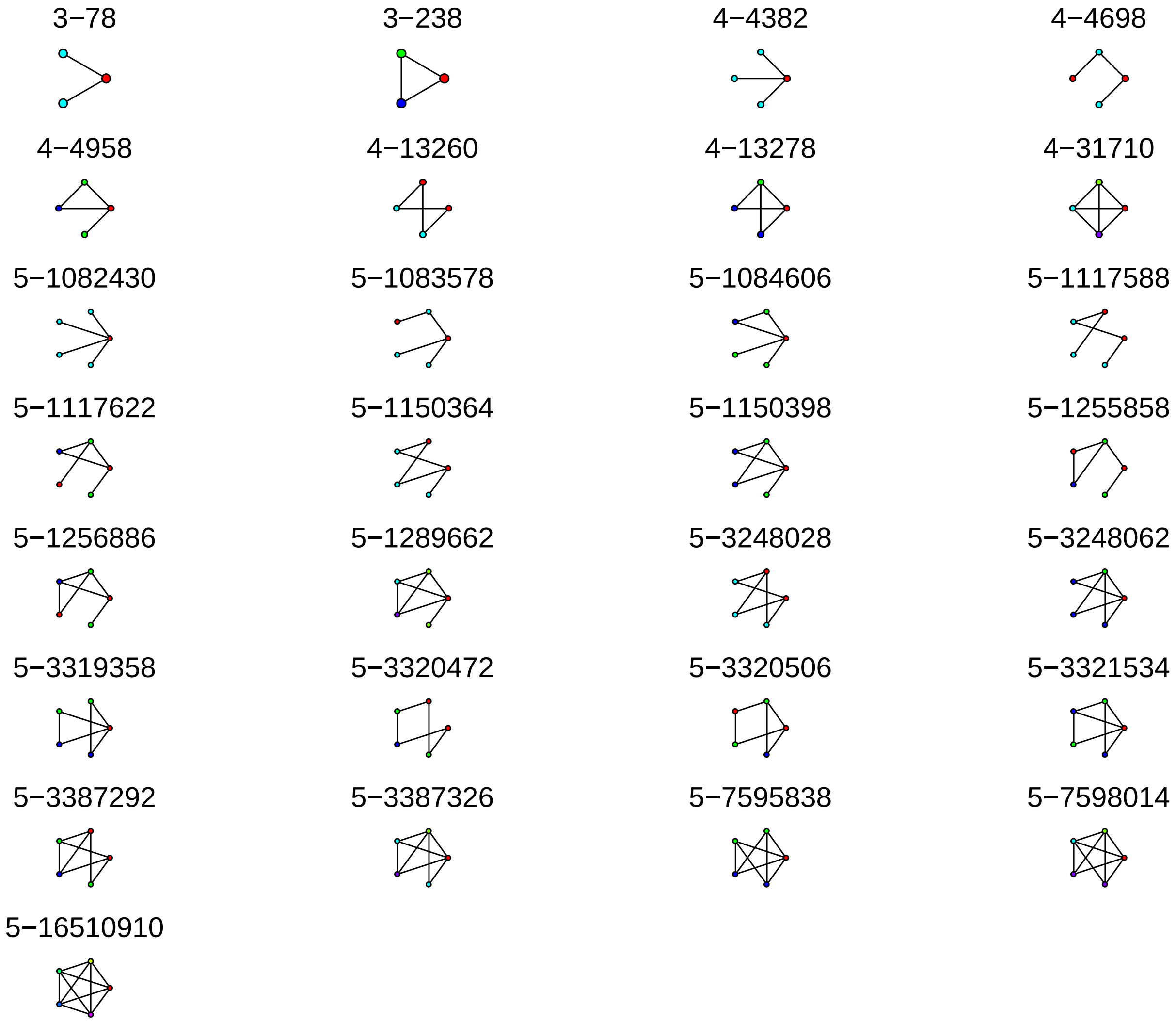

Figure S1.46 Motif Dictionary: all 3- to 5-node subgraphs. Each subgraph is labelled with its size followed by its ID according to mfinder.

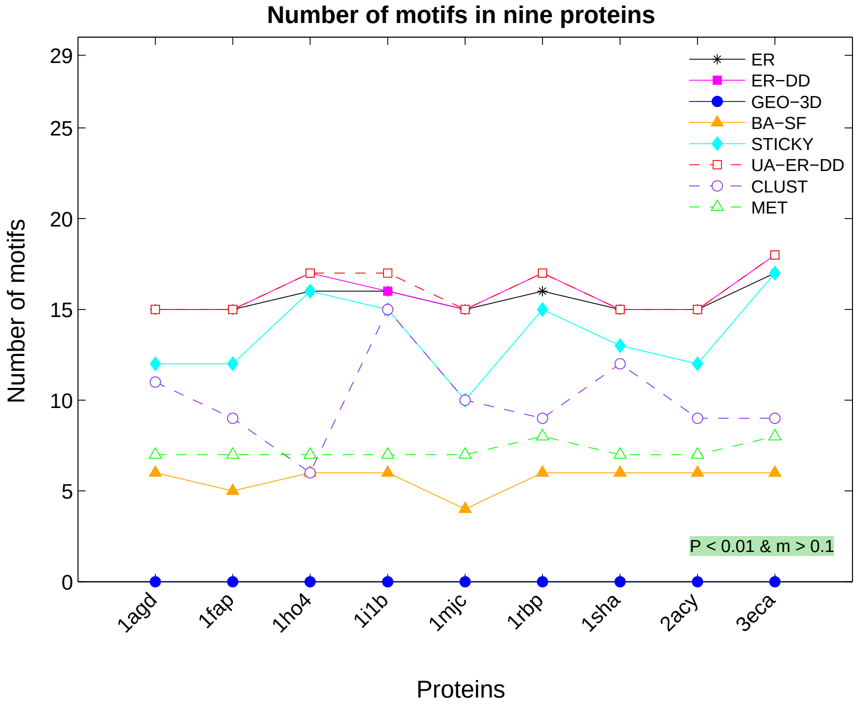

(A)

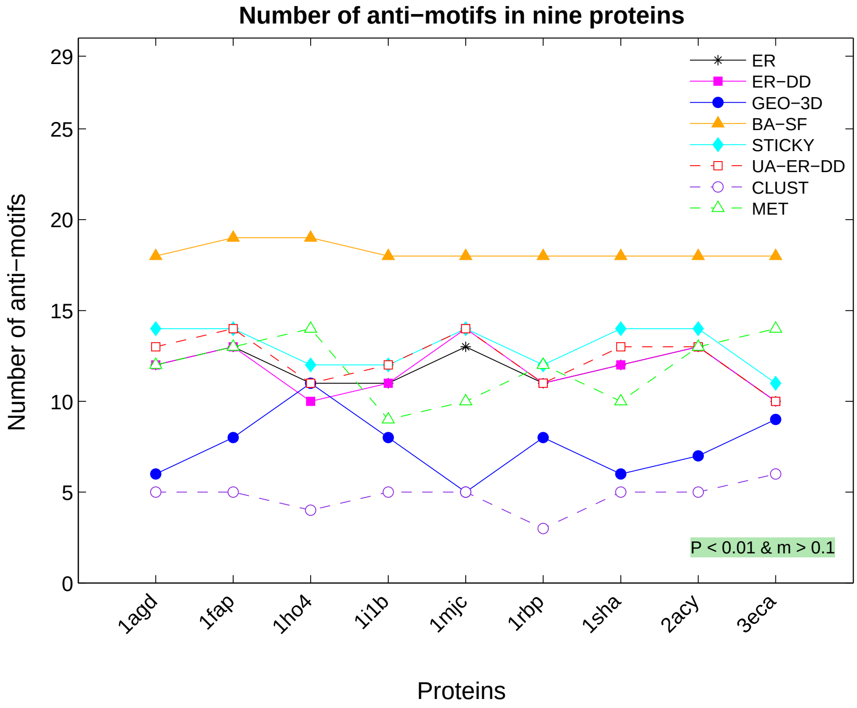

(B)

Figure S1.47 The total number of (A) motifs and (B) anti-motifs identified in nine RIGs corresponding to the nine proteins (1adg, 1fap, 1ho4, 1i1b, 1mjc, 1rbp, 1sha, 2acy, and 3eca), constructed with "ALL" contact type and 5.0 Å distance cut-off. The motifs and anti-motifs were identified with respect to the eight network models (ER, ER-DD, GEO-3D, BA-SF, STICKY, UA-ER-DD, CLUST, and MET). The threshold values used for motif selection (P-value lower than 0.01 and M-factor greater than 0.1) are displayed within the colored textbox.

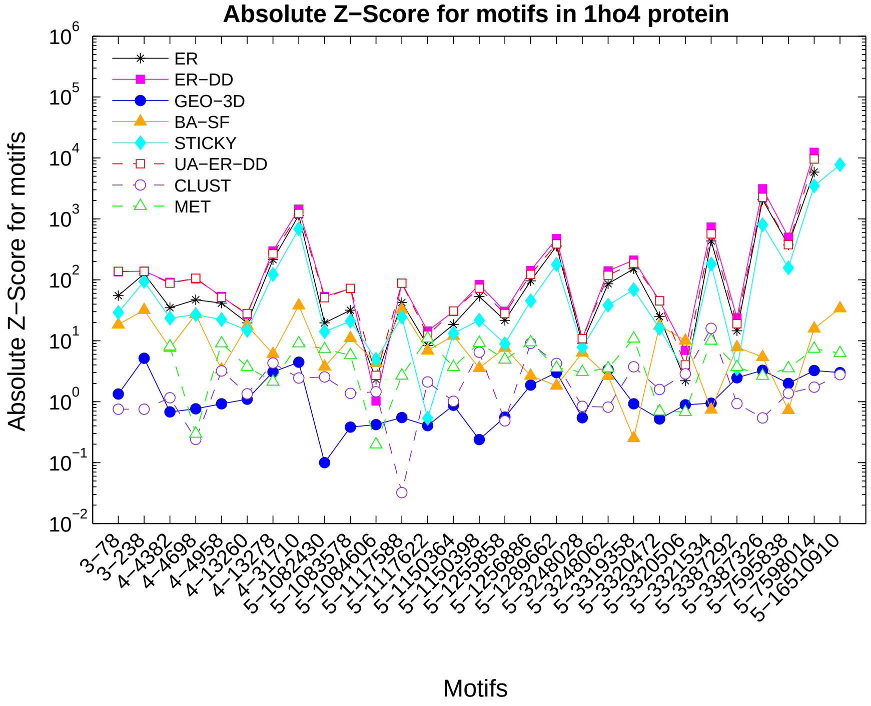

(A)

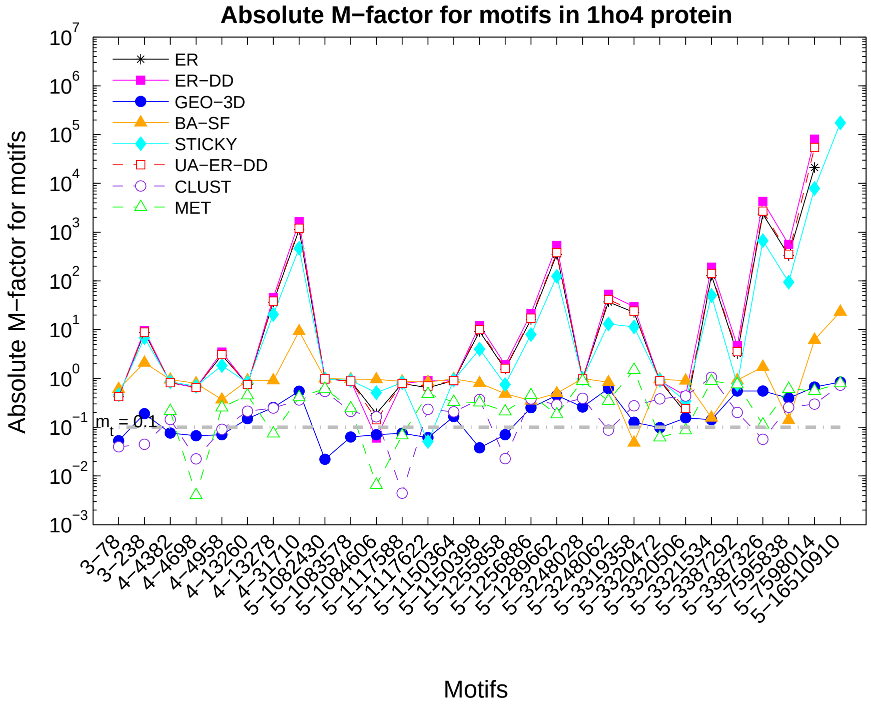

(B)

Figure S1.48 Absolute (A) Z-scores and (B) M-factors for all 3- to 5-node subgraphs in the RIG corresponding to 1ho4 protein, constructed with "ALL" contact type and distance cut-off of 5.0 Å . These statistics were computed with respect to eight network models (ER, ER-DD, GEO-3D, BA-SF, STICKY, UA-ER-DD, CLUST, and MET). Y-axis is shown in a logarithmic scale to facilitate the comparison of different models. The threshold value used for motif selection (M-factor greater than 0.1) is displayed as the grey dash-dot line.

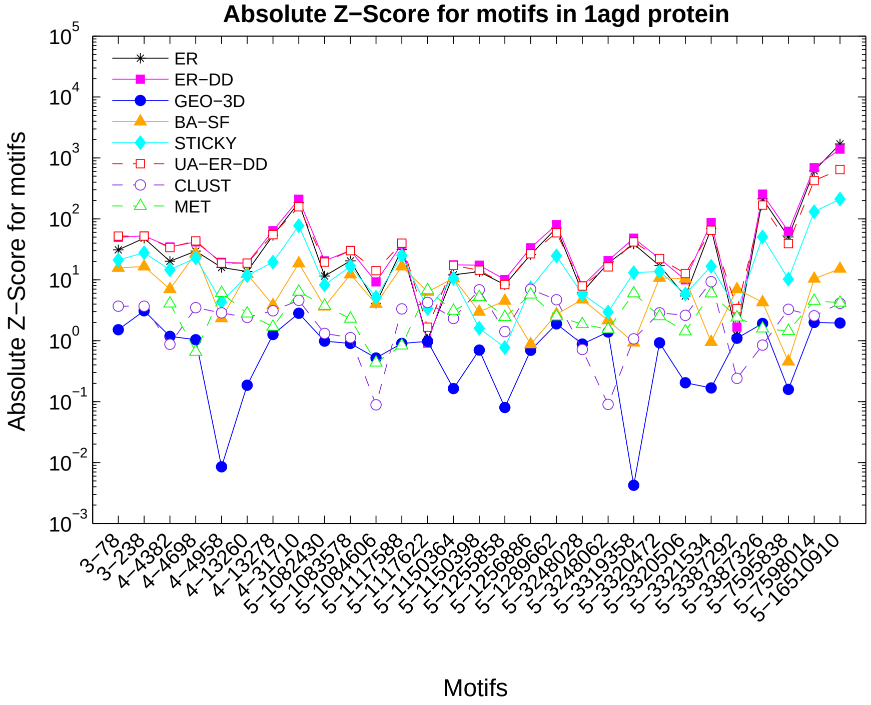

(A)

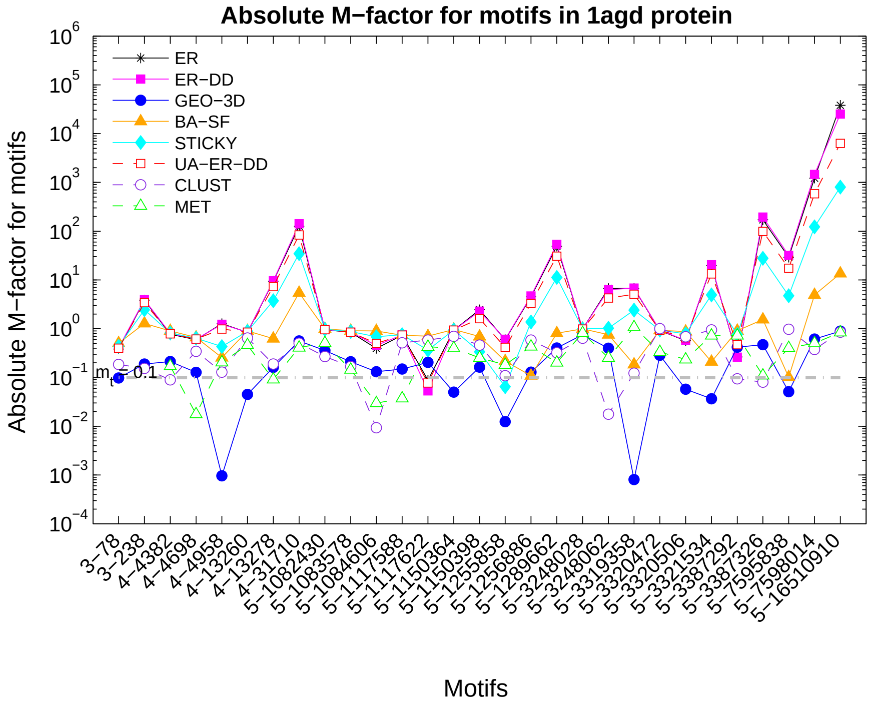

(B)

Figure S1.49 Absolute (A) Z-scores and (B) M-factors for all 3- to 5-node subgraphs in the RIG corresponding to 1agd protein, constructed with "ALL" contact type and distance cut-off of 5.0 Å . These statistics were computed with respect to eight network models (ER, ER-DD, GEO-3D, BA-SF, STICKY, UA-ER-DD, CLUST, and MET). Y-axis is shown in a logarithmic scale to facilitate the comparison of different models. The threshold value used for motif selection (M-factor greater than 0.1) is displayed as the grey dash-dot line.

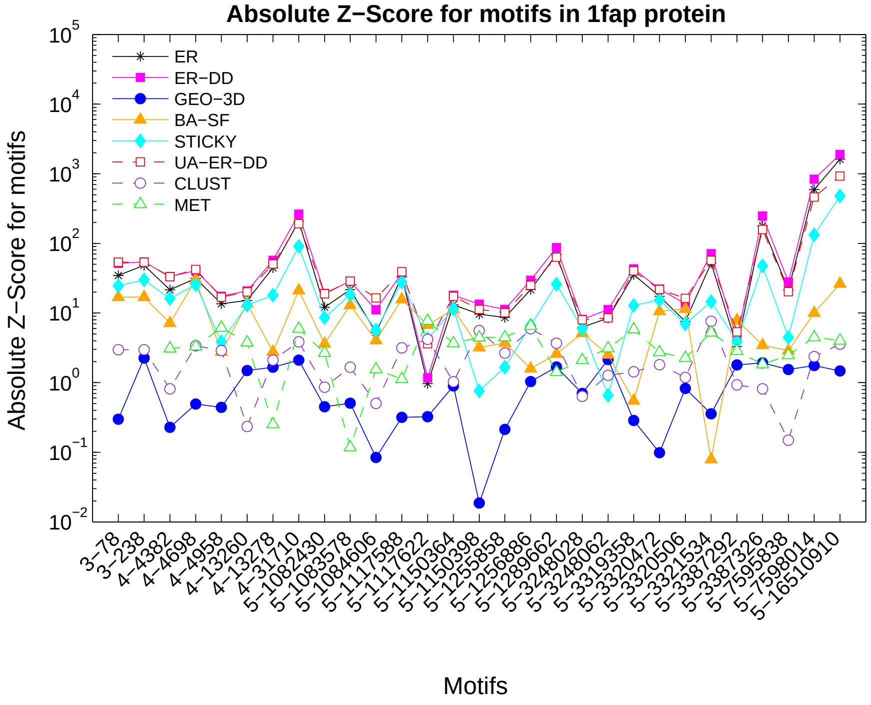

(A)

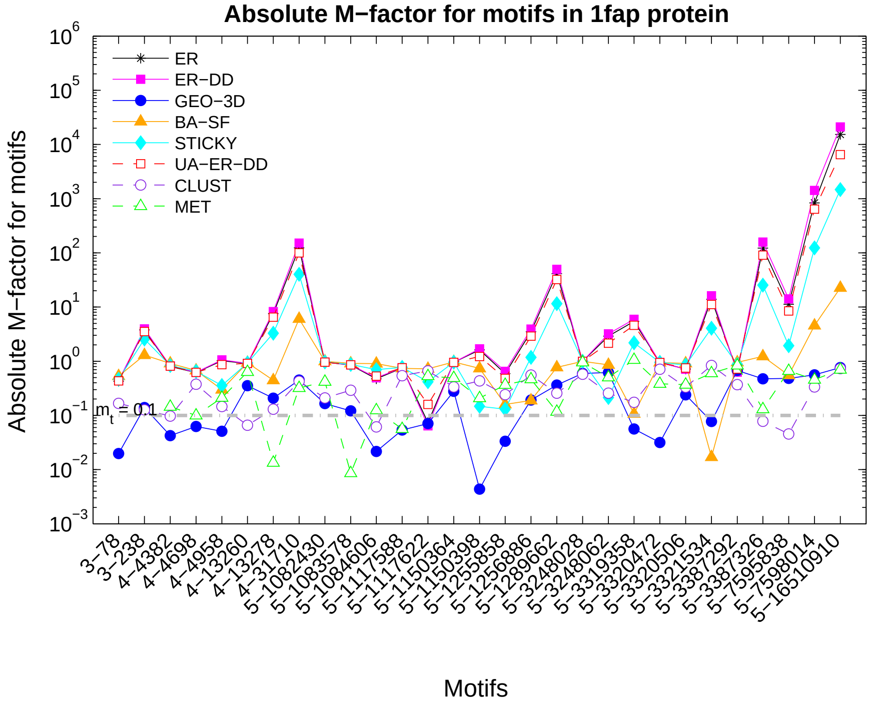

(B)

Figure S1.50 Absolute (A) Z-scores and (B) M-factors for all 3- to 5-node subgraphs in the RIG corresponding to 1fap protein, constructed with "ALL" contact type and distance cut-off of 5.0 Å . These statistics were computed with respect to eight network models (ER, ER-DD, GEO-3D, BA-SF, STICKY, UA-ER-DD, CLUST, and MET). Y-axis is shown in a logarithmic scale to facilitate the comparison of different models. The threshold value used for motif selection (M-factor greater than 0.1) is displayed as the grey dash-dot line.

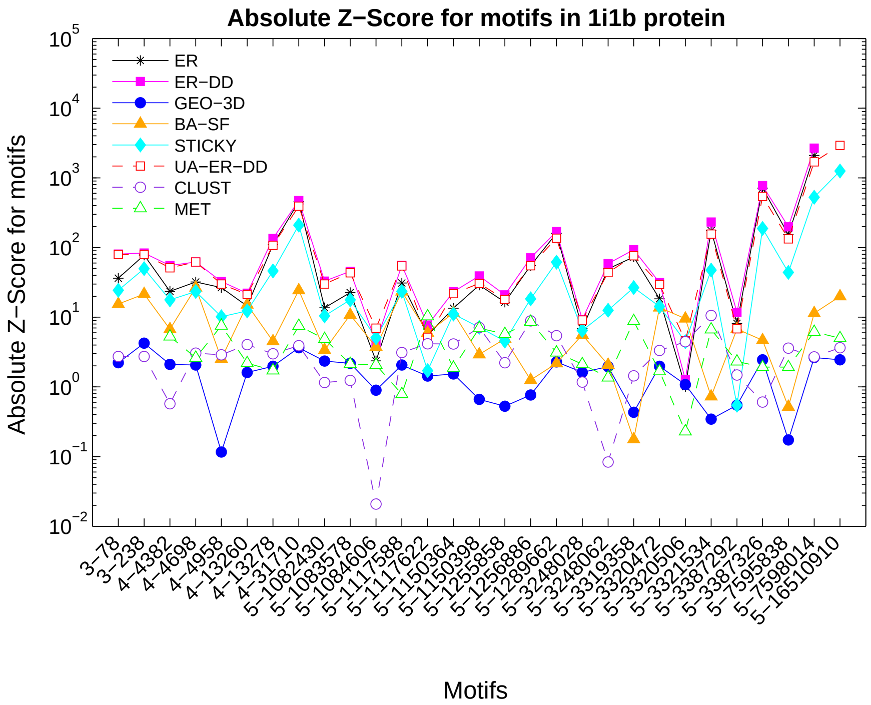

(A)

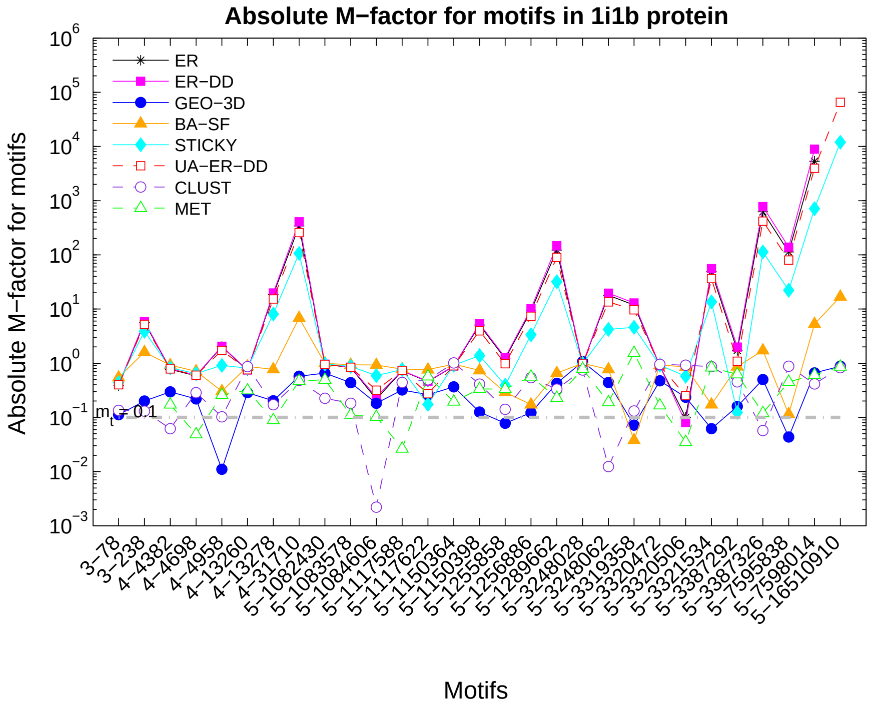

(B)

Figure S1.51 Absolute (A) Z-scores and (B) M-factors for all 3- to 5-node subgraphs in the RIG corresponding to 1i1b protein, constructed with "ALL" contact type and distance cut-off of 5.0 Å . These statistics were computed with respect to eight network models (ER, ER-DD, GEO-3D, BA-SF, STICKY, UA-ER-DD, CLUST, and MET). Y-axis is shown in a logarithmic scale to facilitate the comparison of different models. The threshold value used for motif selection (M-factor greater than 0.1) is displayed as the grey dash-dot line.

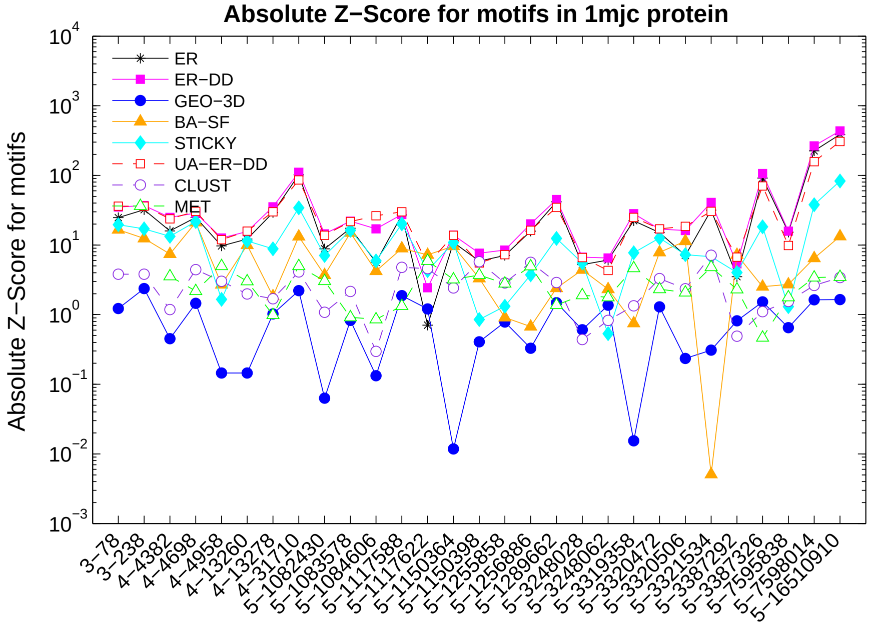

Motifs

(A)

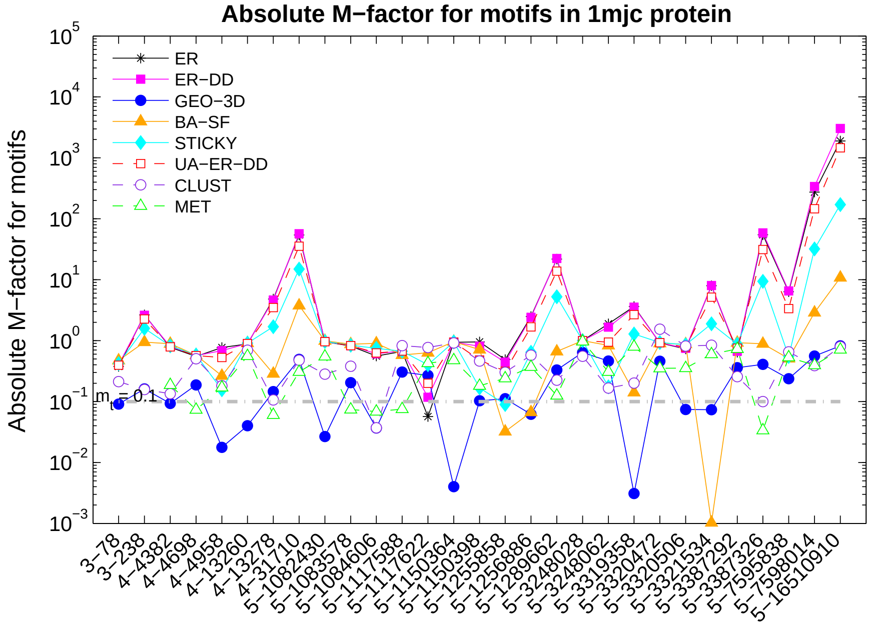

Motifs

(B)

Figure S1.52 Absolute (A) Z-scores and (B) M-factors for all 3- to 5-node subgraphs in the RIG corresponding to 1mjc protein, constructed with "ALL" contact type and distance cut-off of 5.0 Å . These statistics were computed with respect to eight network models (ER, ER-DD, GEO-3D, BA-SF, STICKY, UA-ER-DD, CLUST, and MET). Y-axis is shown in a logarithmic scale to facilitate the comparison of different models. The threshold value used for motif selection (M-factor greater than 0.1) is displayed as the grey dash-dot line.

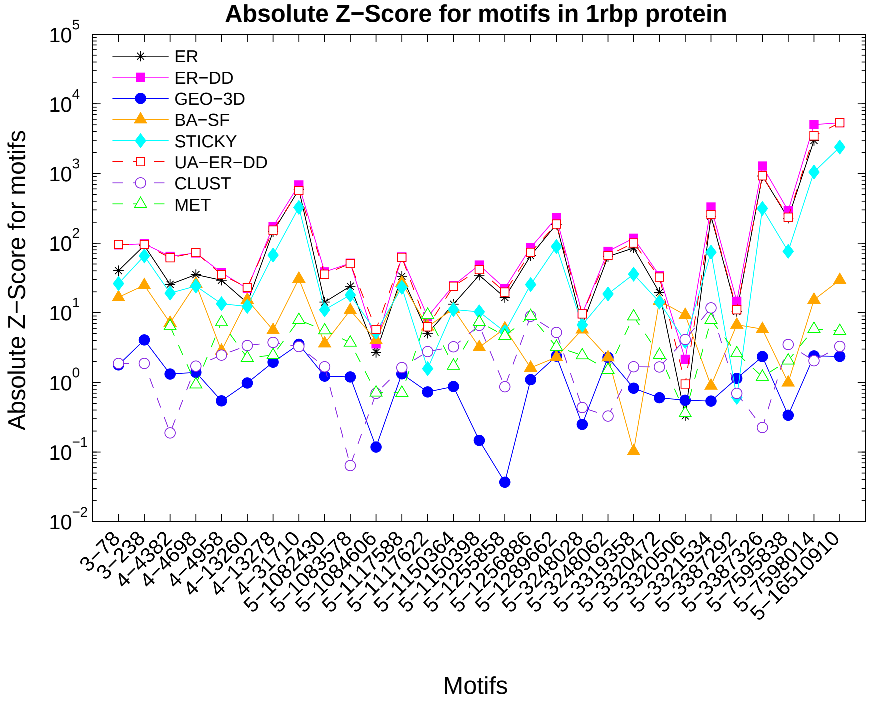

(A)

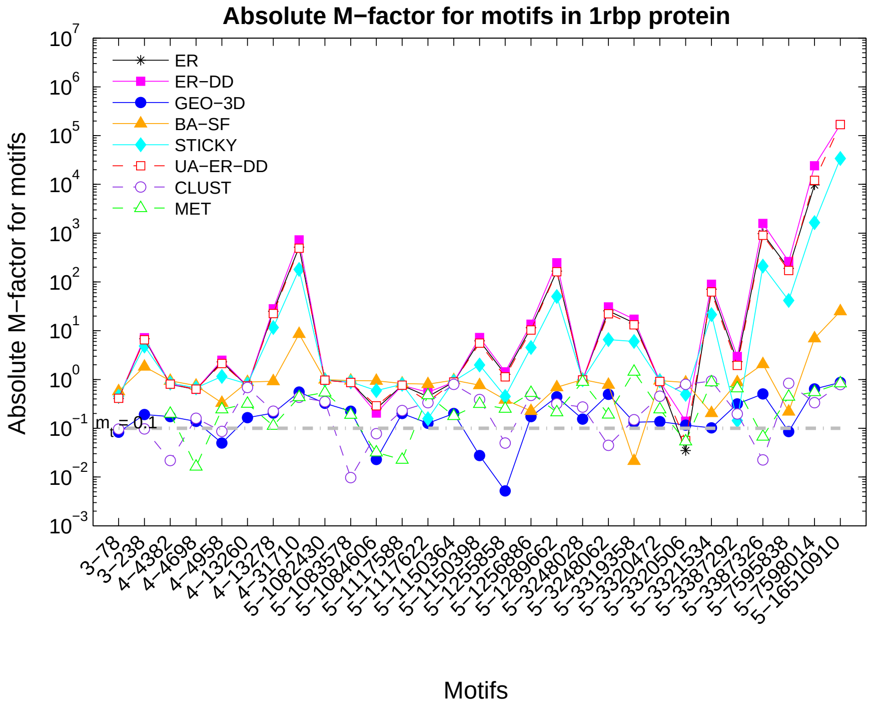

(B)

Figure S1.53 Absolute (A) Z-scores and (B) M-factors for all 3- to 5-node subgraphs in the RIG corresponding to 1rbp protein, constructed with "ALL" contact type and distance cut-off of 5.0 Å . These statistics were computed with respect to eight network models (ER, ER-DD, GEO-3D, BA-SF, STICKY, UA-ER-DD, CLUST, and MET). Y-axis is shown in a logarithmic scale to facilitate the comparison of different models. The threshold value used for motif selection (M-factor greater than 0.1) is displayed as grey the dash-dot line.

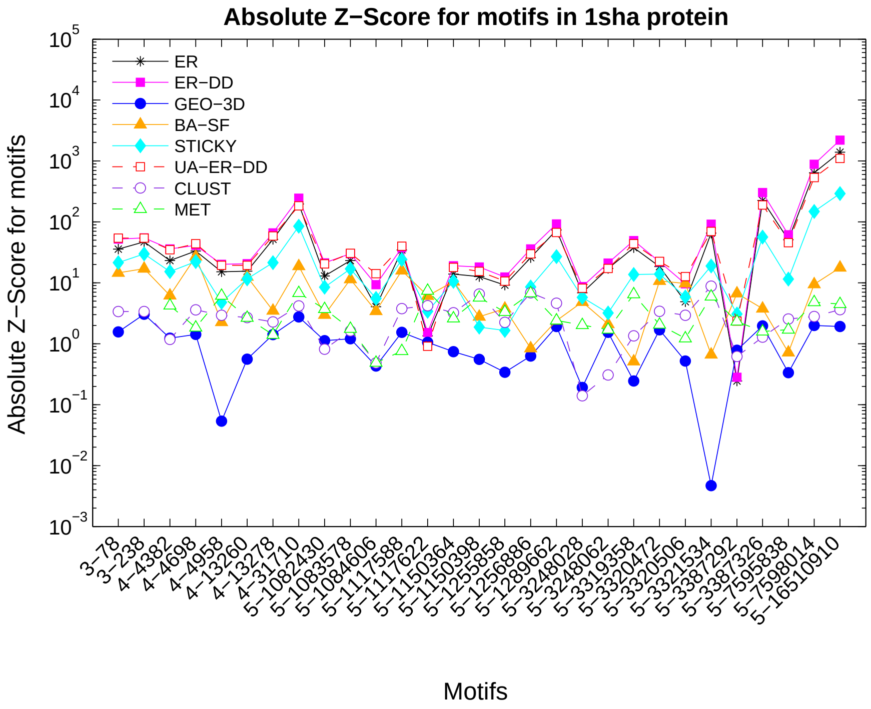

(A)

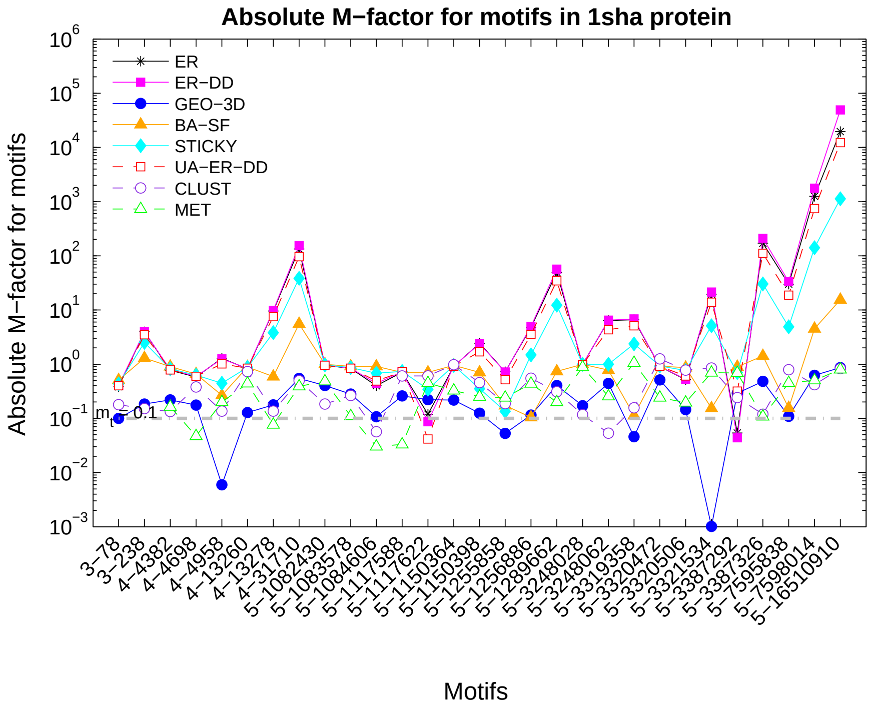

(B)

Figure S1.54 Absolute (A) Z-scores and (B) M-factors for all 3- to 5-node subgraphs in the RIG corresponding to 1sha protein, constructed with "ALL" contact type and distance cut-off of 5.0 Å . These statistics were computed with respect to eight network models (ER, ER-DD, GEO-3D, BA-SF, STICKY, UA-ER-DD, CLUST, and MET). Y-axis is shown in a logarithmic scale to facilitate the comparison of different models. The threshold value used for motif selection (M-factor greater than 0.1) is displayed as the grey dash-dot line.

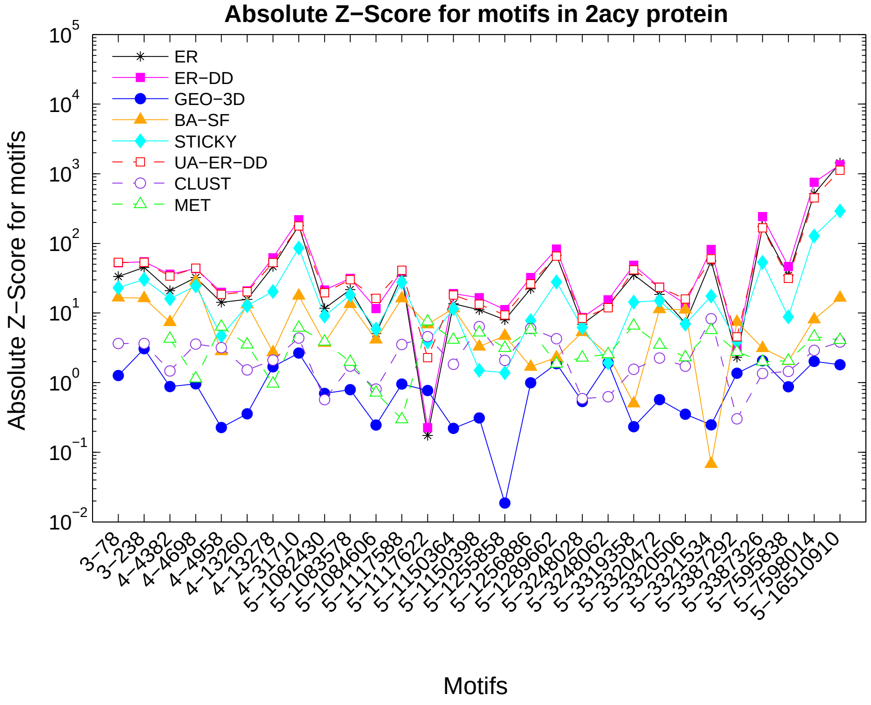

(A)

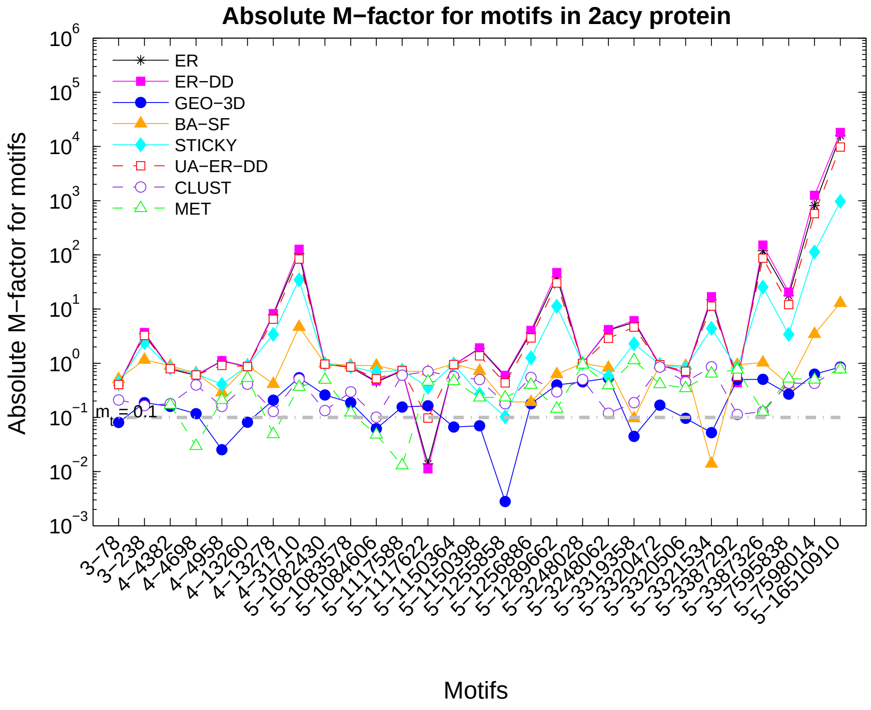

(B)

Figure S1.55 Absolute (A) Z-scores and (B) M-factors for all 3- to 5-node subgraphs in the RIG corresponding to 2acy protein, constructed with “ALL” contact type and distance cut-off of 5.0 Å . These statistics were computed with respect to eight network models (ER, ER-DD, GEO-3D, BA-SF, STICKY, UA-ER-DD, CLUST, and MET). Y-axis is shown in a logarithmic scale to facilitate the comparison of different models. The threshold value used for motif selection (M-factor greater than 0.1) is displayed as the grey dash-dot line.

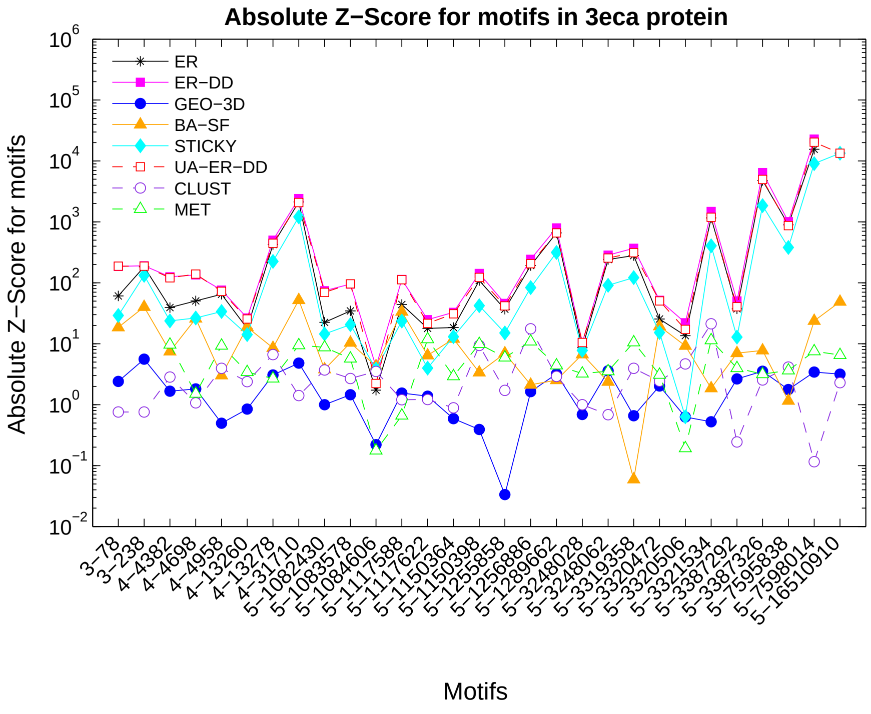

(A)

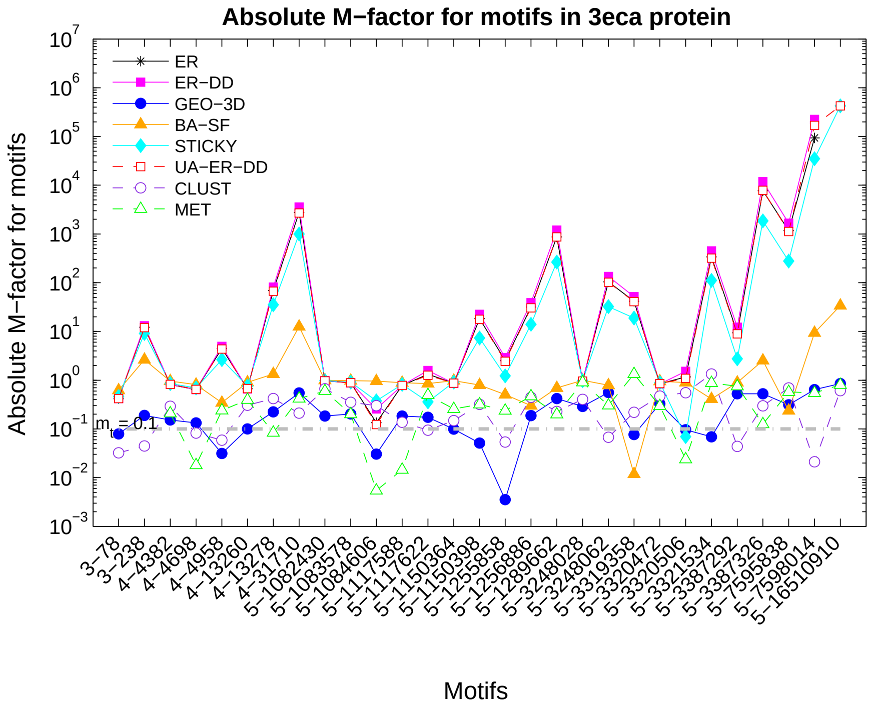

(B)

Figure S1.56 Absolute (A) Z-scores and (B) M-factors for all 3- to 5-node subgraphs in the RIG corresponding to 3eca protein, constructed with "ALL" contact type and distance cut-off of 5.0 Å . These statistics were computed with respect to eight network models (ER, ER-DD, GEO-3D, BA-SF, STICKY, UA-ER-DD, CLUST, and MET). Y-axis is shown in a logarithmic scale to facilitate the comparison of different models. The threshold value used for motif selection (M-factor greater than 0.1) is displayed as the grey dash-dot line.

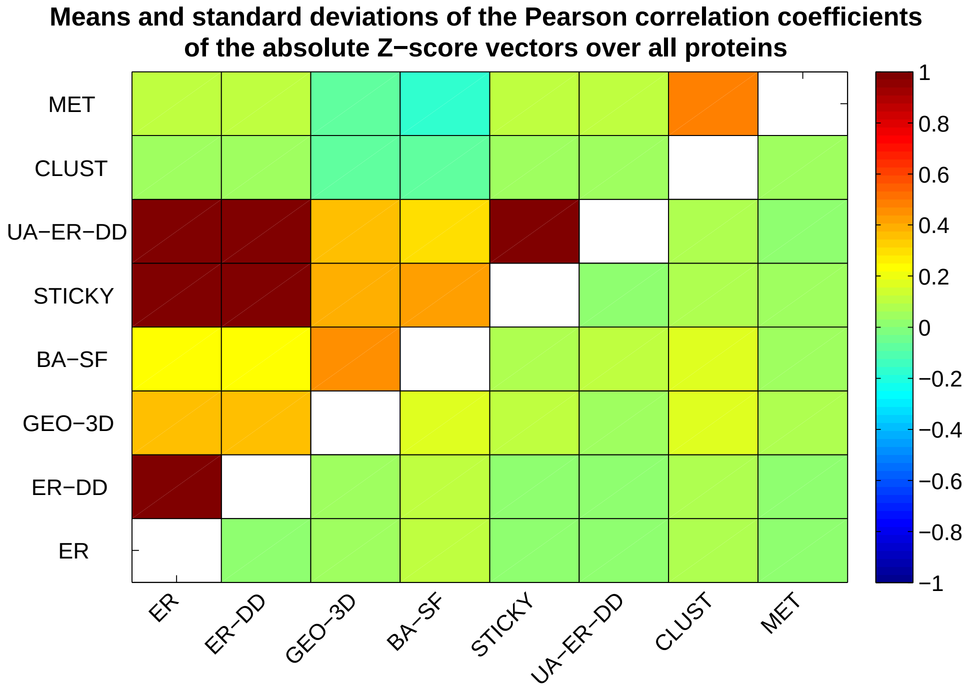

Figure S1.57 Means (upper triangle) and standard deviations (lower triangle) of the Pearson correlation coefficients of the vector of absolute Z-scores of all subgraphs for all possible pairwise combinations of network models over all proteins.
