# Supplementary material for: Optimized Null Model for Protein Structure Networks
Source: PLoS One. 2009 Jun 26;4(6):e5967. doi: 10.1371/journal.pone.0005967 (PMC2699654; doi:10.1371/journal.pone.0005967)
Supplement: Section S1 — Supplementary Text (0.10 MB PDF) [file pone.0005967.s002.pdf]

Section S1.1 Data Sets

Data Set 1 consists of single chain RIGs for nine proteins with the following Protein Data Bank (PDB) [1] [1] codes, followed by the chain identifier whenever applicable: 1agd:B, 1fap:B, 1ho4:A, 1i1b, 1mjc, 1rbp, 1sha:A, 2acy and 3eca:A. Atomic coordinates were taken from the Macromolecular Structure Data Search Database (MSDSD) [2]. All structures are solved by X-ray crystallography and their resolution lies in the range [1.5, 2.7] Å. These proteins are a subset of the non-redundant dataset that Greene and Higman examined [3]. Specifically, they analyzed 65 proteins that cover nine different protein folds, all structural classes and the three kingdoms of life. Moreover, these proteins are diverse in terms of protein sequence and function as well. Since our main concern is our results to be applicable for structurally diverse proteins, we selected one structure from each protein fold randomly for further analysis.

We construct multiple RIGs as undirected, unweighted graphs for each of these proteins. Residues i and j interact if any heavy atom of residue i is within the specified distance cut-off of any heavy atom of residue j. To our knowledge, there is no study that utilizes distance cut-offs less than 4.0 Å. However, we examined the RIGs definition for lower distance cut-offs of 2 Å, 2.5 Å, 3 Å, and 3.5 Å . For these low cut-offs, we noticed that networks that contain backbone-backbone interactions reproduce the polypeptide chain connectivity while "SC" networks become highly disconnected and sparse. To ensure that in all networks at least 80% of the residues have non-covalent contacts, we excluded from our analysis all RIGs defined with distance cut-off less than 4.0 Å. We set distance cut-offs to range from 4.0 to 9.0 Å in increments of 0.5 Å. Most of the studies that utilize contact maps with multiple-atom residue representation use distance cut-offs that lie in the range [4.0, 5.0] Å [3–7]. Therefore, in this range, we choose a finer increment of 0.1 Å. Moreover, we examine various representations of residues, hereafter referred to as *contact types*. We denote by "BB" ("SC") the RIGs that contain as edges only the residue pairs that have heavy backbone (side-chain) atoms within the given distance cut-off. In the case of a "SC" RIG, glycine residues are represented by their Ca atoms. We denote by "ALL" the most commonly used RIG model, in which all heavy atoms of every residue are taken into account when determining residue interactions. Thus, in this data set, we analyze $9 \times 19 \times 3 = 513$ RIGs for the nine proteins constructed for 19 distance cut-offs and the three contact types of "BB", "SC", and "ALL".

Data Set 2 consists of 1,272 RIGs corresponding to 1,272 proteins, constructed with the most commonly used "ALL" contact type and distance cut-off of 5 Å. This non-redundant, representative set of X-ray structures from PDB was pre-compiled by the PISCES server [8]. All proteins have resolution better than 1.8 Å, reliability factor (R-factor) less than or equal to 0.25, and their pairwise sequence similarity does not exceed 20%. Here, we examine whether the strength of the fit of GEO-3D to RIGs changes with respect to protein size and protein structural classes defined by the Structural Classification of Proteins (SCOP) classification, a classification of protein structural domains based on similarities of their amino acid sequences and three-dimensional structures [9]. We analyze 744 out of 1,272 proteins in Data Set 2 that consist of domains with identical structural class and for which SCOP annotation covers more

[1] http://www.pdb.org/

than 90% of the residues. These 744 proteins belong to one of the four different structural classes [9]. An *all-α proteins* (denoted by "A") is a class of structural domains in which the secondary structure is composed entirely of α-helices, with the possible exception of a few isolated β-sheets on the periphery. An *all-β proteins* (denoted by "B") is a class of structural domains in which the secondary structure is composed entirely of β-sheets, with the possible exception of a few isolated α-helices on the periphery. An *α / β proteins* (denoted by "C") is a class of structural domains in which the secondary structure is composed of alternating α-helices and β-strands along the backbone. The β-strands are therefore mostly *parallel*. An *$\alpha + \beta$ proteins* (denoted by "D") is a class of structural domains in which the secondary structure is composed of α-helices and β-strands that occur separately along the backbone. The β-strands are therefore mostly *antiparallel*. Out of 744 proteins, 141 are $all-\alpha$, 161 are $all-\beta$, 221 are $\alpha\,/\,\beta$, and 221 are $\alpha+\beta$. The distribution of protein size for the analyzed proteins with respect to their structural classes is presented in Figure S1.44 A. Only 47 out of the 744 proteins are multi-domain ones and thus are unlikely to bias our analysis. Furthermore, we analyze the relationship between the strength of the fit of GEO-3D to RIGs and the quaternary structure of the corresponding proteins. Out of these 744 proteins, we examine 75 pairs of monodomain monomeric and monodomain multimeric proteins, where poteins within a pair are of equal size and belong to the same structural class, while proteins across pairs may differ in size and class. Proteins in different pairs have from 64 to 390 residues, with average size of 157 ± 77 residues. 13 protein pairs are $all-\alpha$, 12 are $all-\beta$, 21 are $\alpha\,/\,\beta$, and 29 are $\alpha+\beta$.

Data Set 3 consists of 94 pairs of thermophilic *T. maritima* proteins and their mesophilic homologs [10]. Although these mesophilic homologs are distinguished to 62 orthologs and 32 paralogs, the statistically significant differences for structural features responsible for thermostability are consistent in both cases [10]. Therefore, we analyze all 94 pairs as a single data set. We construct RIGs using "ALL" contact type and distance cut-off of 4.5 Å . Although the same criteria was originally used by Robinson-Rechavi et al. [10], our RIG definition is different, since we do not filter out interactions between residues that are less than four residues apart in the primary sequence.

Section S1.2 The quality of the fit of geometric random graph model

Section S1.2.1 Correlation between the fit of GEO-3D and protein size

To examine whether the fit of GEO-3D to RIGs changes with RIG size, we consider data points that are network property values describing the agreement of a RIG of a given size and the GEO-3D model. If there exist more than one RIG of a given size, we average the network property value over all such RIGs. We find that the fit of GEO-3D is strongly correlated with RIG size and that this correlation can be expressed as a power-law function $f = a * x^b + c$. We find such function that fits the data in the least-squares sense, minimizing the sum of squares due to error (also called the summed square of residual), for each of the network properties (Figure S1.39 and Figure S1.40). We quantify the goodness of fit of each of the power-law functions to the observed correlation data with R-Square (RS) measure (Figure S1.40 and Figure S1.41). R-Square illustrates how successful the fit is in explaining the variation of the data; it takes values between 0 and 1, with larger values indicating a better fit. We perform the analysis on

the entire data set of 1,272 RIGs, as well as on each individual class (see Section S1.1). The goodness of fit of the power-law functions for individual classes follows the same trends as for the entire Data Set 2 (Figure S1.41). The fit is good for almost all network properties (RS values above 0.76). The only exceptions are RGF-distance (RS of 0.43), the clustering spectrum, and the spectrum of shortest path lengths (RS values of about 0.17). Note that although average diameters of RIGs and GEO-3D graphs increase and their clustering coefficients decrease with protein size, both following the power-law, the fit of GEO-3D to RIGs with respect to these properties is independent of protein size.

Section S1.2.2 Difference in the fit of GEO-3D across structural classes

We evaluate the statistical significance of the difference of the fit of GEO-3D across structural classes with respect to GDD-agreement. We remove any bias that might exist due to differences in the distribution of protein size for different classes in the following way. We compare the power-law functions that were fitted to the four classes with respect to GDD-agreement (Section S1.2.1, Figure S1.40, and Figure S1.41). The functions are evaluated on the RIG size interval that is common to all classes, with protein size ranging from 87 to 501 residues. We assess the statistical significance of the difference between two functions by performing ANOVA statistical test, with p-values close to 0 strongly suggesting that the values of two functions on a given RIG size interval are drawn from different populations. That is, low p-values indicate that the fit of GEO-3D to proteins of a given size belonging to the classes being compared is significantly different. The p-values illustrating the differences in the fit of GEO-3D over all class pairs are presented in Figure S1.42 A.

Section S1.2.3 Difference in the fit of GEO-3D between thermophilic and mesophilic proteins

We examine the statistical significance of the difference of the fit of GEO-3D graphs to thermophilic and mesophilic proteins with respect to all network properties. We use Student's one-sided paired t-test. The alternative hypothesis is that the mean difference (*diff*) between paired samples is positive, in the direction seen in the observed data. P-values (p) close to zero cast doubts on the null hypothesis and strongly support the alternate hypothesis. We find that the fit of GEO-3D is statistically significantly better for mesophilic than for thermophilic proteins with respect to GDD-agreement ($diff = 0.0087$, $p < 10^{-4}$), degree distribution ($diff = 0.0125$, $p = 0.0006$), and clustering coefficient ($diff = 0.5484$, $p = 0.0054$), as illustrated in Table S1. Moreover, although we use different RIG definition (see Section S1.1) than originally used by Robinson-Rechavi et al. [10], and even though we define protein size to be the number of standard amino acids with structural coordinate data and not the length of the protein as defined in [10], we show that thermophilic proteins are shorter and have higher average connectivity compared to mesophilic proteins, which is in agreement with the original study [10].

Section S1.2.4 Difference in the fit of GEO-3D to monomers and multimers

We determine the effect of the quaternary structure to the fit of GEO-3D to RIGs. We analyze 75 pairs of monomeric and multimeric proteins from Data Set 2. We compare the strength of

the fit of GEO-3D to monomers with its fit to the corresponding multimers using Student's pairwise t-test over all pairs. We do this with respect to each of the network properties. Additionally, we compare clustering coefficients and average diameters of monomers with those of multimers using the same test (Table S2). Although monomers have significantly higher number of contacts per residue and lower average diameter compared to multimers, we observe no significant difference in the fit of GEO-3D between monomers and multimers, with respect of any of the network properties.

Section S1.2.5 Methodology

The accessible surface area and the volume are calculated using the programs calc-surface and calc-volume [12]. Secondary structure is primarily assigned using the program DSSP [13]. Then, the 8-states of DSSP are converted to three secondary structure states according to EVA conversion scheme [14]. Structural class assignment is based on SCOP release 1.73 [9]. Quaternary structure is predicted by PISA server [15], version 1.14.

Section S1.3 Motif Detection

The criteria used to determine the statistical significance of a specific subgraph i are:

- P-value, defined as:

$$P_i = Prob[(Nreal_i \leq Nrand_i) \cup (Nreal_i > Nrand_i)] < 0.01, \tag{1}$$

where $Nrand_i$ is the number of appearances of the pattern i in a randomized network, and $Nreal_i$ is the number of its appearances in the real network. If the subgraph i is over-represented (under-represented) in the real network with respect to randomized networks with probability lower than 0.01, then the subgraph is a motif (anti-motif). To estimate the empirical p-value, we generate 1,000 networks per random graph model.

For plotting purposes (Figure S1.48 to Figure S1.56), as well as for the significance profiles, we use Z-scores instead of P-values. Z-score is defined as:

$$|Z_i| = \frac{|Nreal_i - \bar{N}rand_i|}{sd(Nrand_i)}, \tag{2}$$

where $\bar{N}rand_i$ is the mean number of appearances of the pattern i in the randomized networks, and $sd(Nrand_i)$ is their standard deviation.

- M-factor, defined as:

$$|M_i| = \frac{|Nreal_i - \bar{N}rand_i|}{\bar{N}rand_i} > 0.1 \tag{3}$$

The percentage difference between the number of appearances in the real and the randomized networks must be higher than 0.1. M-factor ensures that subgraphs with just small standard deviation will not be considered misleadingly as significant.

We do not consider the third criteria for network motif selection – uniqueness. Uniqueness is the number of times a subgraph appears in the real network with disjoint set of nodes. Even if uniqueness is less than 4, which is the default threshold in mfinder, there is no reason to reject such subgraphs as non-significant. On the contrary, in RIGs we do not expect motifs that are biologically important to occur many times with completely different set of residues. Also, when a subgraph does not appear in randomized networks of a specific network model, we exclude that subgraph from further motif analysis for that network model.

We use mfinder [16] to search for all undirected subgraphs of 3, 4, and 5 nodes (presented in Figure S1.46) in nine RIGs of "ALL" contact type and 5 Å distance cut-off corresponding to the nine proteins of Data Set 1. In addition to ER, ER-DD, GEO-3D, SF-BA, and STICKY models, we used the three standard models supported by mfinder. We denote these three models as follows: "UA-ER-DD" is the random graph model that preserves the degree distribution of a real-world network, while "CLUST" and "MET" network models, in addition to the degree distribution, preserve the clustering coefficient of all nodes and the number of appearances of all 3-node subgraphs of a real network, respectively. We detect the statistically significant subgraphs according to their P-values, absolute Z-scores, and absolute M-factors, the motif selection criteria proposed by Milo et al. [17] and Kashtan et al. [16], as explained above.

The absolute Z-scores and absolute M-factors of all 3- to 5-node subgraphs in each of the nine RIGs, with respect to each of the eight network models, are presented in Figure S1.48 to Figure S1.56. Since we have already shown that GEO-3D networks provide the best fit to RIGs with respect to graphlet-based measures, subgraphs exhibit low Z-scores and low M-factors when RIGs are compared against geometric random graphs, as expected. On the contrary, with all other network models, a large number of subgraphs have exceptionally high Z-scores and M-factors. Therefore, GEO-3D model exhibits the highest "specificity" in the selection of network motifs. The number of motifs and anti-motifs identified in the nine RIGs with respect to the eight network models are presented in Figure S1.47. We used the same P-value and M-factor thresholds for detection of all (anti-)motifs in all RIGs and with respect to all network models. In all nine RIGs, the fewest number of subgraphs are identified as (anti-)motifs when GEO-3D graphs are used as the null model for (anti-)motif detection.

Also, we address the question of whether different random graph models attribute similar significance to the subgraphs, independent of the magnitude of the significance itself. Similar to the significance profile method [18], we construct 29-dimensional vectors of absolute Z-scores corresponding to 29 3- to 5-node subgraphs where each coordinate represents the Z-score for a given subgraph. For each RIG, we define these vectors with respect to each of the eight network models. Thus, we construct eight vectors of Z-scores for each RIG. Then, we compute Pearson correlation coefficients between all pairs of Z-score vectors for a given RIG. Since the network size is constant in each comparison, there is no need to normalize the Z scores [18]. High Pearson correlation coefficients between Z-score vectors that correspond to two different network models for the same RIG would indicate that both network models assign similar significance, independent of the magnitude of the significance, and thus, by adjusting the Z-score threshold, the same (anti-)motifs would be identified. However, we observed Pearson correlation coefficients lower than 0.5 between vectors corresponding to GEO-3D model and

vectors corresponding to all other network models (see Figure S1.57). Therefore, the results obtained by using geometric network model can not be reproduced with other network models by simple adjustment of the motif selection criteria.

References

[1] Berman, H., Westbrook, J., Feng, Z., Gilliland, G., Bhat, T., Weissig, H., Shindyalov, I., and Bourne, P. (2000) The protein data bank. *Nucleic Acids Research,* **28**, 235–242.

[2] Boutselakis, H., Dimitropoulos, D., Fillon, J., Golovin, A., Henrick, K., Hussain, A., Ionides, J., John, M., Keller, P. A., Krissinel, E., McNeil, P., Naim, A., Newman, R., Oldfield, T., Pineda, J., Rachedi, A., Copeland, J., Sitnov, A., Sobhany, S., Suarez-Uruena, A., Swaminathan, J., Tagari, M., Tate, J., Tromm, S., Velankar, S., and Vranken, W. (Jan, 2003) E-MSD: the European Bioinformatics Institute Macromolecular Structure Database. *Nucleic Acids Res,* **31**(1), 458–462.

[3] Greene, L. H. and Higman, V. A. (Dec, 2003) Uncovering network systems within protein structures. *J Mol Biol,* **334**(4), 781–791.

[4] Heringa, J., Argos, P., Egmond, M. R., and deVlieg, J. (Jan, 1995) Increasing thermal stability of subtilisin from mutations suggested by strongly interacting side-chain clusters. *Protein Eng,* **8**(1), 21–30.

[5] Mirny, L. and Domany, E. (Dec, 1996) Protein fold recognition and dynamics in the space of contact maps. *Proteins,* **26**(4), 391–410.

[6] Singer, M. S., Vriend, G., and Bywater, R. P. (Sep, 2002) Prediction of protein residue contacts with a PDB-derived likelihood matrix. *Protein Eng,* **15**(9), 721–725.

[7] Paszkiewicz, K. H., Sternberg, M. J. E., and Lappe, M. (Jun, 2006) Prediction of viable circular permutants using a graph theoretic approach. *Bioinformatics,* **22**(11), 1353–1358.

[8] Wang, G. and Dunbrack, R. L. J. (Aug, 2003) PISCES: a protein sequence culling server. *Bioinformatics,* **19**(12), 1589–1591 Comparative Study.

[9] Murzin, A. G., Brenner, S. E., Hubbard, T., and Chothia, C. (1995) Scop: a structural classification of proteins database for the investigation of sequences and structures. *Journal of molecular biology,* **247**(4), 536–540.

[10] Robinson-Rechavi, M., Alibes, A., and Godzik, A. (2006) Contribution of Electrostatic Interactions, Compactness and Quaternary Structure to Protein Thermostability: Lessons from Structural Genomics of Thermotoga maritima. *J. Mol. Biol.,* **356**, 547–557.

[11] Jones, S. and Thornton, J. M. (1996) Principles of protein-protein interactions. *Proceedings of the National Academy of Sciences of the United States of America,* **93**(1), 13–20.

[12] Voss, N. R. and Gerstein, M. (2005) Calculation of standard atomic volumes for rna and comparison with proteins: Rna is packed more tightly. *Journal of molecular biology,* **346**(2), 477–492.

[13] Kabsch, W. and Sander, C. (1983) Dictionary of protein secondary structure: pattern recognition of hydrogen-bonded and geometrical features. *Biopolymers,* **22**(12), 2577–2637.

[14] Rost, B. and Eyrich, V. A. (2001) Eva: large-scale analysis of secondary structure prediction. *Proteins,* **Suppl 5**, 192–199.

[15] Krissinel, E. and Henrick, K. (2007) Inference of macromolecular assemblies from crystalline state. *Journal of molecular biology,* **372**(3), 774–797.

[16] Kashtan, N., Itzkovitz, S., Milo, R., and Alon, U. (2004) Efficient sampling algorithm for estimating subgraph concentrations and detecting network motifs. *Bioinformatics,* **20**, 1746–1758.

[17] Milo, R., Shen-Orr, S. S., Itzkovitz, S., Kashtan, N., Chklovskii, D., and Alon, U. (2002) Network motifs: simple building blocks of complex networks. *Science,* **298**, 824–827.

[18] Milo, R., Itzkovitz, S., Kashtan, N., Levitt, R., Shen-Orr, S., Ayzenshtat, I., Sheffer, M., and Alon, U. (2004) Superfamilies of evolved and designed networks. *Science,* **303**, 1538–1542.
