# Supplementary material for: Optimized Null Model for Protein Structure Networks
Source: PLoS One. 2009 Jun 26;4(6):e5967. doi: 10.1371/journal.pone.0005967 (PMC2699654; doi:10.1371/journal.pone.0005967)
Supplement: Table S1 — (0.02 MB PDF) [file pone.0005967.s003.pdf]

Table S1. Pairwise comparison of the fit of GEO-3D to thermophilic proteins and to their mesophilic homologs with respect to different network properties, and comparison of their size and average degrees. The column denoted by “Property” contains the evaluated network property. The column denoted by “Tma” contains the mean value of the corresponding network property for thermophilic proteins. The column denoted by “Meso” contains the mean value of the corresponding network property for their mesophilic homologs. The column denoted by “Difference” contains the mean paired difference between values for thermophilic proteins and values for mesophilic proteins, for a given network property (top number). Also, this column contains p-value (“p”) of the difference in the fit of GEO-3D to Tma and Meso proteins, obtained by Student’s paired t-test (bottom number); statistically significant differences (bolded) are determined with the threshold of p=0.05/9=0.0055, including the Bonferroni correction over 9 tests for 5% significance threshold.

Property	Tma	Meso	Difference
GDD-agreement	0.7586	0.7673	**-0.0087**
			$\boldsymbol{p < 10^{-4}}$
RGF-distance	12.1262	12.1483	-0.0221
			$p = 0.4379$
Pearson correlation between	0.8244	0.8369	**-0.0125**
degree distributions			$\boldsymbol{p = 0.0006}$
Percentage difference of	9.0433	8.4949	**0.5484**
clustering coefficients			$\boldsymbol{p = 0.0054}$
Pearson correlation between	0.4966	0.5282	-0.0316
clustering spectra			$p = 0.0176$
Percentage difference of	6.1568	6.5928	-0.436
average diameters			$p = 0.1183$
Pearson correlation between	0.964	0.9628	0.0013
shortest path lengths spectra			$p = 0.2625$
Protein (RIG) size	252.6702	261.8936	**-9.2234**
			$\boldsymbol{p = 0.0029}$
Average degree	4.6679	4.6029	**0.065**
			$\boldsymbol{p < 10^{-4}}$
