# Supplementary material for: Optimized Null Model for Protein Structure Networks
Source: PLoS One. 2009 Jun 26;4(6):e5967. doi: 10.1371/journal.pone.0005967 (PMC2699654; doi:10.1371/journal.pone.0005967)
Supplement: Table S2 — (0.02 MB PDF) [file pone.0005967.s004.pdf]

Table S2. Pairwise comparison of the fit of GEO-3D to monomeric and to multimeric proteins with respect to different network properties, and comparison of their size, clustering coefficients, and average diameters. The column denoted by "Property" contains the evaluated network property. The column denoted by "Mono" contains the mean value of the corresponding network property for monomeric proteins. The column denoted by "Multi" contains the mean value of the corresponding network property for multimeric proteins. The column denoted by "Difference" contains the mean paired difference between values for monomeric proteins and values for multimeric proteins, for a given network property (top number). Also, this column contains p-value ("p") of the difference in the fit of GEO-3D to Mono and Multi proteins, obtained by Student's paired t-test (bottom number); statistically significant differences (bolded) are determined with the threshold of p=0.05/10=0.005, including the Bonferroni correction over 10 tests for 5% significance threshold.

Property	Mono	Multi	Difference
GDD-agreement	0.7171	0.7153	0.0018
			$p = 0.2584$
RGF-distance	11.2578	11.2188	0.039
			$p = 0.4377$
Pearson correlation between	0.7285	0.73	-0.0014
degree distributions			$p = 0.3964$
Percentage difference of	7.4296	7.323	0.1067
clustering coefficients			$p = 0.3865$
Pearson correlation between	0.3736	0.4062	-0.0325
clustering spectra			$p = 0.0288$
Percentage difference of	5.0247	5.8833	-0.8586
average diameters			$p = 0.1416$
Pearson correlation between	0.9718	0.967	0.0048
shortest path lengths spectra			$p = 0.1081$
Number of edges	813.2733	798.9467	**14.3267**
			$\boldsymbol{p = 0.0038}$
Clustering coefficient	0.5317	0.5364	-0.0048
			$p = 0.0226$
Average diameter	3.4331	3.6439	**-0.2108**
			$\boldsymbol{p = 0.0002}$
